# Phosphorylation and stabilization of EZH2 by DCAF1/VprBP trigger aberrant gene silencing in colon cancer

**DOI:** 10.1038/s41467-023-37883-1

**Published:** 2023-04-17

**Authors:** Nikhil B. Ghate, Sungmin Kim, Yonghwan Shin, Jinman Kim, Michael Doche, Scott Valena, Alan Situ, Sangnam Kim, Suhn K. Rhie, Heinz-Josef Lenz, Tobias S. Ulmer, Shannon M. Mumenthaler, Woojin An

**Affiliations:** 1 Department of Biochemistry and Molecular Medicine, Norris Comprehensive Cancer Center, University of Southern California, Los Angeles, CA 90033 USA; 2 Lawrence J. Ellison Institute for Transformative Medicine, University of Southern California, Los Angeles, CA 90064 USA; 3 Department of Biochemistry and Molecular Medicine, Zilkha Neurogenetic Institute, University of Southern California, Los Angeles, CA 90033 USA; 4 Division of Medical Oncology, Norris Comprehensive Cancer Center, University of Southern California, Los Angeles, CA 90033 USA

**Keywords:** Molecular medicine, Cancer

## Abstract

Our recent work has shown that DCAF1 (also known as VprBP) is overexpressed in colon cancer and phosphorylates histone H2AT120 to drive epigenetic gene inactivation and oncogenic transformation. We have extended these observations by investigating whether DCAF1 also phosphorylates non-histone proteins as an additional mechanism linking its kinase activity to colon cancer development. We now demonstrate that DCAF1 phosphorylates EZH2 at T367 to augment its nuclear stabilization and enzymatic activity in colon cancer cells. Consistent with this mechanistic role, DCAF1-mediated EZH2 phosphorylation leads to elevated levels of H3K27me3 and altered expression of growth regulatory genes in cancer cells. Furthermore, our preclinical studies using organoid and xenograft models revealed that EZH2 requires phosphorylation for its oncogenic function, which may have therapeutic implications for gene reactivation in colon cancer cells. Together, our data define a mechanism underlying DCAF1-driven colonic tumorigenesis by linking DCAF1-mediated EZH2 phosphorylation to EZH2 stability that is crucial for establishing H3K27me3 and gene silencing program.

## Abstract (web summary)

DCAF1/VprBP contributes to epigenetic gene inactivation and cancer development. Here the authors show that DCAF1/VprBP phosphorylates and stabilises EZH2 to induce oncogenic gene silencing and colon cancer growth.

## Introduction

The DDB1 and CUL4 Associated Factor 1 (DCAF1), also known as VprBP (HIV-1 Vpr Binding Protein), was initially identified as a protein interacting with HIV-1 Vpr and has been implicated as a regulator of cell cycle and cell proliferation^1–4^. While most studies on DCAF1 have focused on its adaptor function in the Cullin 4 A E3 ubiquitin ligase complex, our recent study uncovered the presence of intrinsic kinase activity in DCAF1 and identified H2AT120 as its first phosphorylation target^5^. Regarding DCAF1 functions, our gene expression profiling established gene-selective corepressor function of DCAF1 specifically targeting and silencing growth regulatory genes in cancer cells. DCAF1 inactivates growth regulatory genes in a manner dependent on H2AT120 phosphorylation (H2AT120p), as a point mutation of T120 in H2A eliminates the ability of DCAF1 to repress transcription in the chromatin context. Also, kinase-dead mutations almost completely abolished the transrepression potential of DCAF1 in cancer cells, implying H2AT120p-dependent mechanism for DCAF1 function in maintaining inactive chromatin states and inducing oncogenic transformation^5,6^. A role for DCAF1-mediated H2AT120p in cancer development is further supported by the observation that DCAF1 expression and H2AT120p levels are higher in several cancer types, especially colon cancer^6^. Given the importance of DCAF1 kinase activity in tumorigenesis and the significance of DCAF1-mediated H2AT120p in inactivating growth regulatory genes, we also identified a small molecule inhibitor, named B32B3, capable of inhibiting DCAF1 kinase activity and tumor growth in organoid and xenograft models^5,6^. Although these data clearly linked DCAF1-mediated H2AT120p to epigenetic gene inactivation in cancer cells, one of the remaining questions is whether phosphorylation of non-histone proteins is also critical for DCAF1-promoted oncogenic events. This question is important since DCAF1-mediated phosphorylation of other proteins might generate distinct properties of gene regulatory factors at particular loci, creating posttranslational mechanisms that drive oncogenic cell signaling.

Enhancer of Zeste Homolog 2 (EZH2) is a highly conserved histone lysine methyltransferase that catalyzes the trimethylation of nucleosomal histone H3 at lysine 27 (H3K27me3)^7,8^. EZH2 partners with embryonic ectoderm development (EED) and Suppressor of Zeste 12 (SUZ12) to form the Polycomb Repressive Complex (PRC2) and carry out its optimal histone methyltransferase activity^9,10^. EZH2 is overexpressed or mutated in a wide spectrum of cancers and associates with tumor initiation and progression as well as poor clinical prognosis and outcomes^11–15^. EZH2-mediated H3K27me3 is thought to initiate tumorigenesis through a variety of mechanisms, which ultimately establish the silenced chromatin environment and prevent the expression of tumor suppressor genes^16–18^. Of special relevance to the current study, a series of reports also showed that the stability and enzymatic activity of EZH2 are controlled by several types of posttranslational modifications. Phosphorylation is probably the most well-known process which modulates the molecular property and function of EZH2. For instance, AKT1 and CDK1 phosphorylate EZH2 at serine 21 (S21) and threonine 487 (T487), respectively, and dissociate EZH2 from the PRC2 complex^19,20^. In breast cancer cells, AMPK-mediated phosphorylation at T311 attenuates EZH2 enzymatic activity toward H3K27^21^, and p38-mediated phosphorylation at T367 stimulates EZH2 cytoplasmic localization and metastasis^22^. In addition to these observations, EZH2 phosphorylation has also been implicated in the process of switching on its enzymatic activity and repressing tumor suppressor genes, stressing the importance of postsynthetic regulation of EZH2 function in oncogenesis^23,24^. While all these data support phosphorylation-induced dysregulation of EZH2 activities in certain cancer contexts, further investigation in this aspect is still needed and exactly how EZH2 phosphorylation modulates its function in the process of tumorigenesis remains unclear.

In the present study, we show that DCAF1 is overexpressed and phosphorylates EZH2 in colon cancer cells. Mass spectrometry analysis identified T367 of EZH2 as the major phosphorylation site for DCAF1, which was further confirmed by using EZH2T367 phosphorylation (EZH2T367p)-specific antibody that we developed. DCAF1-mediated EZH2T367p is oncogenic because DCAF1 knockdown or inhibition reactivates a large set of tumor suppressor genes and impedes cancer cell proliferation. Furthermore, we provide evidence that pharmacologically targeting DCAF1 and EZH2 in organoid and xenograft models is sufficient to impair their ability to induce oncogenic gene silencing as well as colonic tumor growth.

## Results

### DCAF1 increases EZH2 protein levels in colon cancer cells

**Fig. 1:**
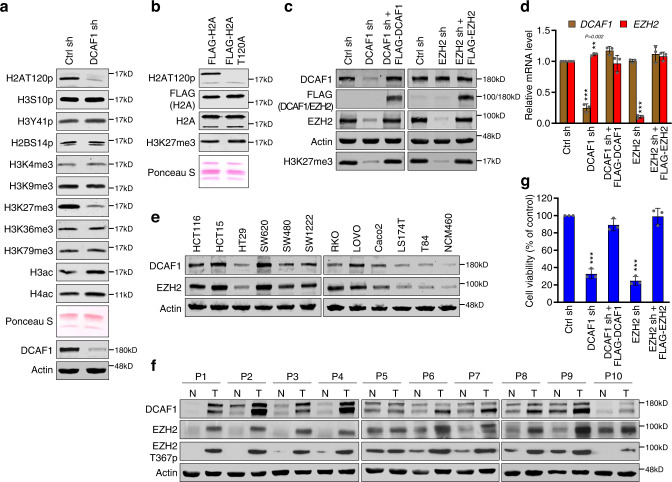
DCAF1-dependent increase in EZH2 protein levels in colon cancer cells. **a** SW620 colon cancer cells were transfected with nontargeting control shRNA (Ctrl) or DCAF1 targeting shRNA, and chromatin fractions were prepared and analyzed by Western blotting with antibodies specific for the indicated histone modifications. The top panel shows a Ponceau S-stained gel of the purified chromatin. Shown on the bottom panel is Western blot analysis confirming DCAF1 knockdown. Actin was used as a loading control. Data are representative of three independent experiments. **b** SW620 cells were infected with lentiviruses expressing H2A wild type or T120A mutant containing an N-terminal 3×FLAG tag, and nucleosomes were prepared from chromatin fractions. Nucleosomes containing ectopic H2A were immunoprecipitated from total nucleosomes with anti-FLAG antibody. The relative levels of H2AT120p and H3K27me3 in the purified nucleosomes were determined by Western blotting. Histone compositions of the purified nucleosomes were also analyzed by 15% SDS-PAGE followed by Ponceau S staining (bottom panel). Data are representative of three independent experiments. **c** Cell lysates and chromatin fractions were prepared from SW620 cells depleted of DCAF1 or EZH2 and analyzed by Western blotting with the antibodies indicated on the left. To rescue the knockdown effects, DCAF1- or EZH2-depleted cells were infected with lentiviruses expressing shRNA-resistant 3×FLAG-DCAF1 or 3×FLAG-EZH2. Data are representative of three independent experiments. (See also Supplementary Fig. 1a). **d** Total RNA was isolated from DCAF1- or EZH2-depleted SW620 cells complemented with 3×FLAG-DCAF1 or 3×FLAG-EZH2 and subjected to RT-qPCR analysis with DCAF1 and EZH2 specific primers listed in Supplementary Table 4. Data are represented as mean ± SEM of three independent experiments. P values were calculated using two-way analysis of variance (ANOVA) with post-hoc Tukey’s test for multiple comparisons. **P < 0.01 and ***P < 0.001 versus Ctrl sh. (See also Supplementary Fig. 1b). **e** Cell lysates were prepared from normal colon (NCM460) and colon cancer (HCT116, HCT15, HT29, SW620, SW480, SW1222, RKO, LOVO, Caco2, LS174T, and T84) cell lines and analyzed by Western blotting as in (**c**). Data are representative of three independent experiments. (See also Supplementary Fig. 2). **f** Western blot analyses of DCAF1 and EZH2 expression levels as well as EZH2T367p states in human colon tumor and their adjacent normal tissue samples. Actin was used as a loading control. Data are representative of three independent experiments. **g** DCAF1- or EZH2-depleted SW620 colon cancer cells were complemented with 3×FLAG-DCAF1 or 3×FLAG-EZH2, and their growth was assessed after 72 h culture using the cell proliferation reagent WST-1. Data are represented as mean ± SEM of three independent experiments. *P* values were calculated using one-way ANOVA with post-hoc Tukey’s test for multiple comparisons. ***P < 0.001 versus Ctrl sh. (See also Supplementary Fig. 1c) Source data are provided as a Source Data file.

We recently reported that DCAF1 is overexpressed and represses gene transcription through H2AT120p in colon cancer cells^6^. In extending these studies, we first examined whether other histone modifications are also subject to regulation by DCAF1 in colon cancer cells. In agreement with our published data, depletion of endogenous DCAF1 in SW620 colon cancer cells drastically reduced the levels of H2AT120p in chromatin fractions. Under these conditions, our Western blot analysis also detected an apparent decrease in H3K27me3 after DCAF1 knockdown (Fig. 1a). This led us to speculate that DCAF1-mediated H2AT120p may indirectly influence H3K27me3 in colon cancer cells, because H2AT120 lies close to the H3 N-terminal tail. To check this possibility, we expressed FLAG-H2A wild type or T120A mutant and prepared nucleosomes containing the ectopic H2A. In checking H3K27me3 levels by Western blotting, comparable levels of H3K27me3 were detected with nucleosomes carrying ectopic H2A wild type or T120A mutant (Fig. 1b). These results illustrate that DCAF1 exerts its stimulatory effects on H3K27me3 in an H2AT120p-independent manner. As EZH2 is mainly responsible for H3K27me3 in colon cancer^25^, the observed dependency of H3K27me3 on DCAF1 in colon cancer cells also encourages the possibility that DCAF1 may be a critical upstream regulator of EZH2 activity. To test this, we depleted DCAF1 and EZH2 in SW620 cancer cells overexpressing DCAF1 and EZH2 by using a lentiviral shRNA infection system. After confirming high knockdown efficiencies for DCAF1 and EZH2 shRNAs, cell lysates and chromatin fractions were prepared and analyzed by Western blotting. A noteworthy observation emerged from our analysis was that DCAF1 depletion remarkably decreased EZH2 protein levels and thus attenuated H3K27me3 in SW620 cells (Fig. 1c, left panel). Expression of ectopic DCAF1 in DCAF1-depleted SW620 cells increased EZH2 protein levels quantitatively similar to those observed for mock-depleted control cells (Fig. 1c, left panel). On the contrary, EZH2 knockdown and rescuing expression had no effect on DCAF1 protein levels (Fig. 1c, right panel). Similar results were obtained with another colon cancer cell line Caco2 (Supplementary Fig. 1a). Since we detected equivalent EZH2 mRNA levels in control and DCAF1-depleted SW620 and Caco2 cells (Fig. 1d and Supplementary Fig. 1b), these results indicate a role for DCAF1 in mediating the stabilization and accumulation of EZH2 protein in colon cancer cells. These findings were corroborated by additional Western blot and band intensity analyses showing that DCAF1 protein levels were high and quantitatively well correlates with increased EZH2 protein levels in other colon cancer cell lines (Fig. 1e and Supplementary Fig. 2). Moreover, much higher levels of DCAF1 protein in human colon tumor samples and a strong correlation between protein levels for DCAF1 and EZH2 (Fig. 1f) are further indicative of a DCAF1 role in enhancing EZH2 protein stability in colon cancer cells. In line with these observations and consistent with our recent study implicating DCAF1 in colon cancer^6^, cell viability assays over a period of 3 days reproducibly showed that SW620 and Caco2 cells grow much more slowly following the depletion of endogenous DCAF1 (Fig. 1g and Supplementary Fig. 1c). Additional analysis after expressing FLAG-DCAF1 in DCAF1-depleted colon cancer cells clearly showed almost complete restoration of cell growth rates. Similarly, EZH2 knockdown also led to a significant decrease in cell growth compared with mock-depleted control cells, and ectopic expression of EZH2 rescued the growth potential of cancer cells (Fig. 1g and Supplementary Fig. 1c).

### DCAF1 phosphorylates EZH2 in colon cancer cells

**Fig. 2:**
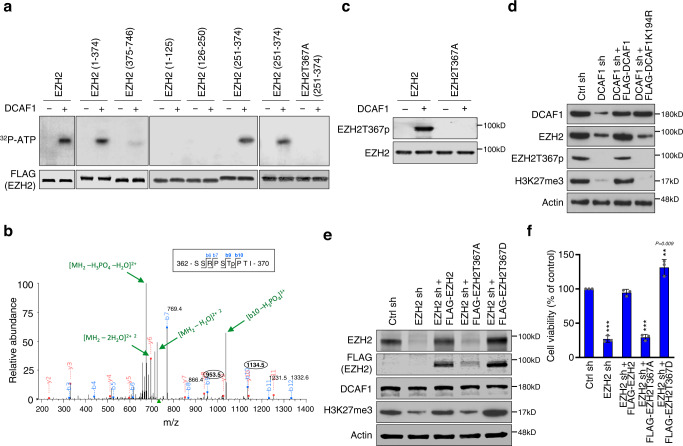
Phosphorylation of EZH2 at T367 by DCAF1. **a** The indicated recombinant EZH2 proteins were incubated with DCAF1 in the presence of [γ-^32^P] ATP for 30 min. The reactions were then resolved on 5–20% SDS-PAGE and analyzed by autoradiography (upper panel) and Western blot (lower panel). Data are representative of three independent experiments. **b** EZH2 251-374 was phosphorylated by DCAF1, digested with trypsin, and analyzed by liquid chromatography-tandem mass spectrometry (LC-MS/MS) identifying the presence of phosphorylated T367. **c** EZH2 wild type or T367A mutant proteins were modified by DCAF1 and analyzed by Western blot using the antibody raised against EZH2T367p. Data are representative of three independent experiments. (See also Supplementary Fig. 3). **d** DCAF1-depleted SW620 cells were infected with lentiviruses expressing 3×FLAG-DCAF1 wild type and K194R kinase-dead mutant as indicated on the top. Cell lysates were prepared and subjected to Western blot analysis with indicated antibodies. Data are representative of three independent experiments. **e** EZH2-depleted SW620 cells were infected with lentiviruses expressing 3×FLAG-EZH2 wild type, T367A phospho-blocking mutant, or T367D phospho-mimicking mutant as indicated on the top. Aliquots of cell lysates were analyzed by Western blot. Data are representative of three independent experiments. **f** EZH2-depleted SW620 cells were rescued as in (**e**), and WST-1 assays were carried out after 3 days of culture. Data are represented as mean ± SEM of three independent experiments. P values were calculated using one-way ANOVA with post-hoc Tukey’s test for multiple comparisons. **P < 0.01 and ***P < 0.001 versus Ctrl sh. (**a**), (**c**–**f**) Source data are provided as a Source Data file.

The above-described studies demonstrated a role for DCAF1 in mediating the stabilization of EZH2 protein in colon cancer cells. One possible mechanism for the observed influence could involve a direct phosphorylation of EZH2 by DCAF1. To examine this possibility, we incubated full length EZH2 protein with recombinant DCAF1 in the presence of [γ-^32^P]-ATP. As shown in Fig. 2a, DCAF1 generated a clear radiolabeling of EZH2 after autoradiography. In similar experiments with truncated versions of EZH2, autoradiograph of the kinase reaction products showed a robust phosphorylation of the central region (residues 251-374) of EZH2. As a more direct approach toward delineating the exact location of phosphorylation, the phosphorylated EZH2 251-374 fragment was sequenced by tandem mass spectrometry (MS/MS). This analysis identified only a singly phosphorylated peptide that could have arisen from phosphorylation of threonine 367 (T367) (Fig. 2b). To further investigate the predicted role for EZH2T367 phosphorylation (EZH2T367p), we raised a rabbit polyclonal antibody that recognizes EZH2T367p. The specificity of the purified antibody was verified by dot blot and peptide competition assays using the unmodified and phosphorylated peptides (Supplementary Fig. 3). In analyzing in vitro phosphorylation reactions with this antibody, we could confirm that DCAF1 efficiently catalyzes EZH2T367p and that alanine mutation of T367 (T367A) completely abolishes DCAF1-mediated EZH2T367p (Fig. 2c). More intriguingly, much lower levels of EZH2 were detected in DCAF1-depleted SW620 cancer cells compared to mock-depleted control cells, and the observed reduction of EZH2 correlated well with the disappearance of EZH2T367p (Fig. 2d). Ectopic expression of DCAF1 wild type, but not DCAF1K194R kinase-dead mutant, in DCAF1-depleted cells restored the level of EZH2T367p to ~80% of that observed in mock-depleted control cells, a finding in line with the notion that DCAF1 is essential for EZH2T367p process in colon cancer cells (Fig. 2d). Given that DCAF1 and EZH2 levels are significantly correlated among colon tumor samples, we also expected the involvement of DCAF1 in catalyzing EZH2T367p during colonic tumorigenesis. In this regard, we observed that the levels of DCAF1 and EZH2T367p are well correlated with each other in the majority of tumor samples (Fig. 1f).

For the purpose of further validating the positive impact of DCAF1-mediated EZH2T367p on EZH2 stability and function, we expressed EZH2 mutants that harbor alanine substitution (T367A) to block or aspartic acid substitution (T367D) to mimic T367 phosphorylation in EZH2-depleted SW620 colon cancer cells. Western blot analysis of cell lysates clearly indicated that a decrease in EZH2 protein levels and cancer cell growth rate after EZH2 knockdown cannot be restored by ectopically expressing EZH2T367A (Fig. 2e). Conversely, when EZH2T367A was replaced by EZH2T367D in our rescue experiments, EZH2-depleted SW620 cancer cells showed a considerable rise in EZH2 protein and H3K27me3 levels, to even higher levels compared with EZH2 wild type (Fig. 2e). Consistent with these data, the expression of EZH2T367D, but not EZH2T367A, markedly boosts the capacity of EZH2-depleted SW620 colon cancer cells to growth (Fig. 2f). We next wished to investigate to what extent the enzymatic activity and protein stability of EZH2 are regulated by T367p per se. In DCAF1-depleted SW620 cancer cells, ectopic EZH2 wild type and EZH2T367A phospho-blocking mutant showed low stead-state protein levels and minimal growth stimulatory effects (Supplementary Figs. 4a, b). However, expressing phospho-mimicking EZH2T367D mutant in DCAF1-depleted cells caused a robust increase in EZH2 protein and H3K27me3 levels as well as cell growth rates (Supplementary Figs. 4a, b), echoing the contribution of DCAF1 to EZH2 protein stability and function in a way that depends on T367p. To determine whether DCAF1-mediated EZH2T367p happens in the nucleus or cytoplasm, subcellular fractions were also prepared from SW620 colon cancer cells and analyzed by Western blotting. Endogenous DCAF1 and EZH2 were mainly detected in the nuclear fraction of SW620 cells, and stable depletion of DCAF1 significantly decreased EZH2T367p with parallel reduction in EZH2 protein and H3K27me3 levels (Supplementary Fig. 5a). Supporting these observations, both EZH2T367D phospho-mimicking and EZH2T367A phospho-blocking mutants also showed nuclear localization despite more efficient nuclear accumulation of EZH2T367D compared to EZH2T367A (Supplementary Fig. 5b). These results emphasize that DCAF1 is mainly responsible for catalyzing EZH2T367p and increasing EZH2 concentration in the nucleus of colon cancer cells.

### DCAF1-mediated phosphorylation stabilizes EZH2

**Fig. 3:**
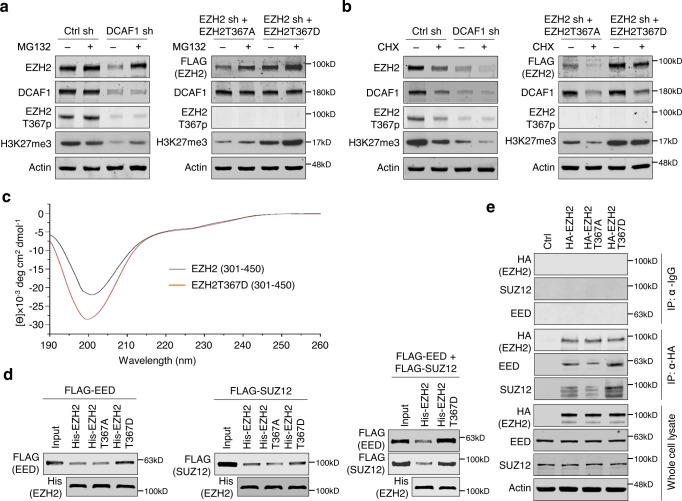
Regulation of EZH2 degradation by DCAF1-mediated phosphorylation. **a** Control (Ctrl) and DCAF1-depleted SW620 colon cancer cells were treated with MG132 (10 µM) for 12 h, and cell lysates were collected and immunoblotted with antibodies against EZH2, DCAF1, EZH2T367p, and H3K27me3 (left panel). EZH2-depleted SW620 cells were infected with lentiviruses expressing 3×FLAG-EZH2T367A phospho-blocking mutant and T367D phospho-mimicking mutant followed by treatment with MG132 (10 µM) for 12 h and analyzed (right panel). Actin was used as a loading control. Data are representative of three independent experiments. **b** SW620 cells used in (A) were treated with CHX (50 µg/ml) for 6 h, and cell lysates were prepared and analyzed by Western blotting. Data are representative of three independent experiments. **c** Circular dichroism spectra of wild type EZH2 (300-450) and EZH2T367D (300-450) were recorded at protein concentrations of 5 µM in spectrum buffer (20 mM K_2_HPO_4_/KH_2_PO_4_, pH 7.4, 25 mM KCl, and 100 µM TCEP) at 25 °C using a JASCO J-810 spectropolarimeter. The observed ellipticity in millidegrees, θ, was converted into the mean residue ellipticity, θ_MRW_. **d** His-tagged EZH2 wild type, T367A mutant, and T367D mutant were immobilized on Ni-NTA-agarose and incubated with FLAG-SUZ12 and FLAG-EED. After extensive washing, bound SUZ12 and EED proteins were fractionated by 10% SDS-PAGE, and probed with anti-FLAG antibody. Input lanes represent 20% of SUZ12 and EED used in the binding reactions. Data are representative of three independent experiments. **e** HA-EZH2 wild type, T367A mutant, and T367D mutant were expressed in SW620 cells and immunoprecipitated using anti-HA antibody. The binding of endogenous EED and SUZ12 to HA-EZH2 was analyzed by Western blotting. Data are representative of three independent experiments. (**a**, **b**, **d**, **e**) Source data are provided as a Source Data file.

As phosphorylation often acts as a signal to target the proteins to the ubiquitin-proteasome pathway^26^, we next wanted to examine whether the above-described effects of DCAF1-mediated EZH2T367p on EZH2 reflects an alteration of proteomic degradation processes. Toward this end, we treated SW620 colon cancer cells with the proteasome inhibitor MG132 for 12 h, and evaluated its impact on EZH2 protein levels by Western blotting. Because DCAF1 efficiently stabilizes EZH2 protein in SW620 cells, MG132 treatment generated only a modest increase in the levels of endogenous EZH2 protein and thus T367p as compared with mock-treated control cells (Fig. 3a, left panel). Meanwhile, when DCAF1-depleted SW620 cells were treated with MG132, EZH2 protein levels were increased to those comparable with mock-depleted control cells, but there were no detectable changes in EZH2T367p (Fig. 3a, left panel). In analogous experiments using EZH2-depleted cells rescued with EZH2T367A and EZH2T367D, MG132 treatment generated an apparent increase in the stability of those ectopic EZH2 mutants (Fig. 3a, right panel). That DCAF1 protein levels were unchanged following MG132 treatment argues strongly that the observed alterations in EZH2 protein levels are specific (Fig. 3a, right panel). Additionally, treating control and DCAF1-depleted SW620 cells with the protein synthesis inhibitor cycloheximide (CHX) led to a sharp decline in the levels of EZH2 and DCAF1 proteins (Fig. 3b, left panel). CHX treatments of EZH2T367A-transfected, EZH2-depleted SW620 cells also showed obvious changes with respect to the stabilities of EZH2 and DCAF1 proteins. However, the inability of CHX to decrease EZH2 protein levels in EZH2T367D-transfected, EZH2-depleted SW620 cells supported the view that DCAF1-mediated T367p is sufficient to generate the higher stability of EZH2 in colon cancer cells (Fig. 3b, right panel).

Although our data clearly implicate DCAF1-mediated EZH2T367p in EZH2 stabilization in colon cancer cells, they left open the molecular basis for the observed T367p effects. EED and SUZ12 are known to interact with EZH2 and form the trimeric PRC2 complex. Considering that EED and SUZ12 stabilize EZH2^27–29^ but are not in direct contact with EZH2T367 in the complex (Supplementary Fig. 6), we suspected that DCAF1-mediated T367p alters the conformational state of EZH2, causing it to become more favorable for the physical association with SUZ12 and EED. To study this possibility, we first used circular dichroism spectroscopy and compared the structural characteristics of EZH2 wild type and T367D phospho-mimicking mutant. A noteworthy observation emerged from this analysis is a change in the overall secondary structure content of EZH2 when T367D mutation is introduced, and these results support an involvement of DCAF1-mediated T367p in altering EZH2 structural conformation (Fig. 3c). In order to gain further insight into how EZH2T367p antagonizes EZH2 degradation, we then examined to which extent EZH2T367p is involved in regulating the interaction between EZH2 and EED/SUZ12. When each of EED and SUZ12 was checked for binding to EZH2 by Western blotting, their direct interaction with EZH2 was detected (Fig. 3d, left and middle panels). In similar binding experiments with EZH2T367D, a higher affinity binding of EED/SUZ12 was seen (Fig. 3d, left and middle panels). When EED and SUZ12 were used together, they showed much more efficient interaction with EZH2T367D, further indicating that EZH2T367p is essential for their continuous association (Fig. 3d, right panel). As another experiment to prove the importance of EZH2T367p, cell lysates were prepared from SW620 cells transfected with HA-EZH2 wild type and mutants, and EZH2-EED/SUZ12 interaction was examined by Western blotting of immunoprecipitated materials. As shown in Fig. 3e, endogenous EED and SUZ12 efficiently co-precipitated with ectopic EZH2 wild type, but minimally did so with EZH2T367A phospho-blocking mutant. These observations argue that EZH2T367p is likely the primary regulator of EZH2-EED/SUZ12 interaction in colon cancer cells. In fact, there was about 3-4 fold increase in EZH2 interaction with EED/SUZ12 when EZH2 wild type was replaced by EZH2T367D phospho-mimicking mutant (Fig. 3e). The importance of DCAF1 with respect to the observed interaction was further confirmed by the finding that DCAF1 knockdown almost completely abrogates the ability of EZH2 to interact with EED and SUZ12 in colon cancer cells (Supplementary Fig. 7). Taken together, these results strongly argue that DCAF1-mediated T367p causes long-range conformational changes in EZH2 through allosteric mechanisms and stabilizes its interaction with EED and SUZ12, thereby stimulating its H3K27me3 catalyzing activity.

### DCAF1 and EZH2 regulate shared target genes in colon cancer cells

**Fig. 4:**
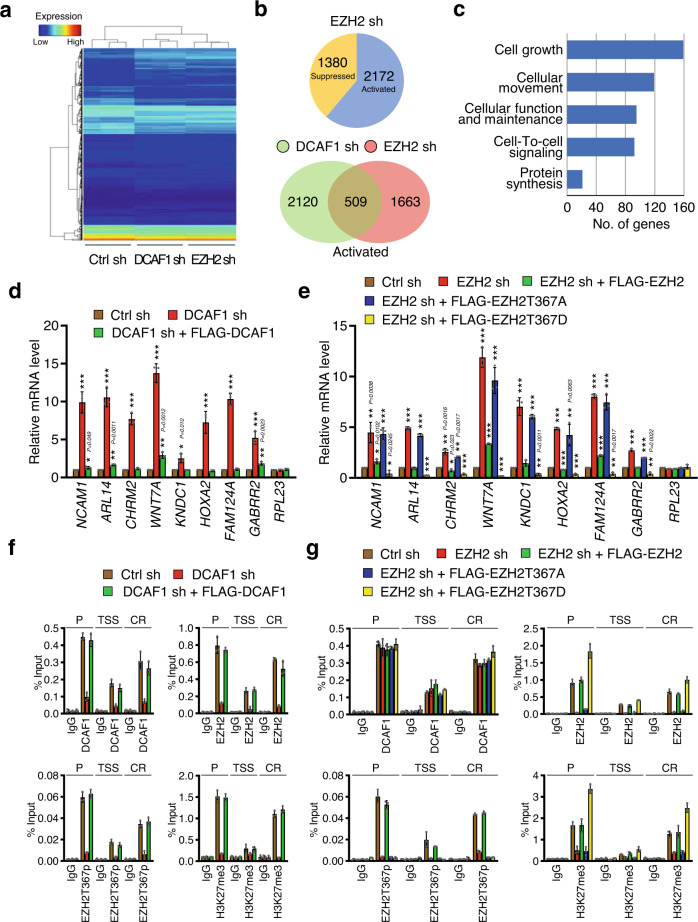
A significant fraction of target genes shared by DCAF1 and EZH2. **a** Heat map of genes differentially regulated in control, DCAF1-depleted, or EZH2-depleted SW620 colon cancer cells. Data show three biological replicates. (See also Supplementary Fig. 8). **b** Venn diagram showing genes that were upregulated or downregulated (>1.5 fold; FDR < 0.05) in DCAF1-depleted or EZH2-depleted SW620 cells compared to mock-depleted control cells. **c** Gene ontology analysis of genes that were commonly activated in DCAF1-depleted and EZH2-depleted SW620 cells using Qiagen Ingenuity Pathway Analysis software. **d** RNA-seq data were validated by RT-qPCR using primers specific for the 8 genes that were upregulated and the 1 gene that was unaffected following DCAF1 knockdown. Also included in this analysis was mRNA extracted from DCAF1-rescued SW620 cells. The values are expressed as fold changes from the mRNA levels in mock-depleted control cells. Primer sequences are listed in Supplementary Table 4. Data represent the mean ± SEM of three independent experiments. *P* values were calculated using two-way ANOVA with post-hoc Tukey’s test for multiple comparisons. *P < 0.05, **P < 0.01 and ***P < 0.001 versus Ctrl sh. (See also Supplementary Fig. 11a). **e** RT-qPCR assays were carried out as in (**d**), but using EZH2-depleted SW620 cells. For rescue experiments, EZH2-depleted cells were transfected with shRNA-resistant EZH2 wild type, T367A mutant, or T367D mutant. Data are represented as mean ± SEM of three independent experiments. *P* values were calculated using two-way ANOVA with post-hoc Tukey’s test for multiple comparisons. **P < 0.01 and ***P < 0.001 versus Ctrl sh. (See also Supplementary Fig. 11b). **f** ChIP assays were performed in mock-depleted (Ctrl sh) and DCAF1-depleted (DCAF1 sh) SW620 cells using antibodies against DCAF1, EZH2, EZH2T367p and H3K27me3 as indicated. To rescue the effects of DCAF1 knockdown, shRNA-resistant DCAF1 was expressed. Precipitation efficiencies relative to non-enriched input samples were determined for the three locations across the *ARL14* locus by qPCR with primers listed in Supplementary Table 5. Percent input is determined as the amount of immunoprecipitated DNA relative to input DNA. Data are represented as mean ± SEM of three independent experiments. (See also Supplementary Figs. 12a, c). **g** ChIP assays were as described in (F), but using EZH2-depleted SW620 cells transfected with shRNA-resistant EZH2 wild type, T367A mutant, or T367D mutant. Data are represented as mean ± SEM of three independent experiments. (See also Supplementary Figs. 12b, d) (**c**–**g**) Source data are provided as a Source Data file.

Recently, our genome-wide analysis revealed that DCAF1 plays a major role in negatively regulating growth regulatory genes in SW620 colon cancer cells (GEO: GSE180282)^6^. To assess the functional contributions made by DCAF1-mediated EZH2T367p in these results, we next investigated whether there are any correlations between DCAF1- and EZH2-regulated gene expression in SW620 cells. As summarized by Venn diagram in Fig. 4b and indicated by heat maps and volcano plots in Fig. 4a and Supplementary Fig. 8, transcriptome analysis of total RNA isolated from control and EZH2-depleted cells by RNA-seq identified 2172 genes activated and 1380 genes suppressed at least 1.5-fold upon EZH2 knockdown. When these new data sets were compared with our published RNA-seq data from DCAF1-depleted SW620 cells (GEO: GSE180282)^6^ after evaluating their quality control (QC) metrics (Supplementary Fig. 9), we found that 509 out of the 2172 activated genes are also up-regulated over 1.5-fold in response to DCAF1 knockdown. Ingenuity Pathways Analysis (IPA) of the up-regulated genes revealed significant enrichments in pathway related to several cancer-specific categories, such as cell growth, cellular movement, and cell-to-cell signaling, indicating overall reactivation of anti-tumorigenic gene-expression programs in DCAF1- and EZH2-depleted cells (Fig. 4c). A significant enrichment of endoplasmic reticulum (ER) stress response genes was also evident from our Gene Ontology (GO) and Reactome pathway analyses of the 509 genes commonly upregulated upon DCAF1 and EZH2 knockdown (Supplementary Figs. 10a, b and Supplementary Table 1). The role for DCAF1-mediated EZH2T367p in ER stress response was further supported by our functional enrichment analyses using the list of significantly enriched GO terms (Supplementary Fig. 10c and Supplementary Table 2). Given that ER stress response constitutes a key signaling event in cancer development and progression, these findings functionally implicate DCAF1-mediated EZH2T367p in colon cancer. To validate our RNA-seq data, we conducted reverse transcription quantitative PCR (RT-qPCR) analysis of 8 target genes that were downregulated by both DCAF1 and EZH2. The selected genes encode factors controlling cell proliferation/growth and acting as tumor suppressors in several types of cancers including colon cancer^30–36^. Our analysis with total RNA from SW620 and Caco2 cells clearly demonstrated the up- and down-regulation of the eight selected targets after DCAF1 knockdown and rescue expression, respectively (Fig. 4d and Supplementary Fig. 11a). EZH2 knockdown also caused 3-13 fold increases in target gene transcription, and the expression of EZH2 wild type, but not EZH2T367A phospho-blocking mutant, fully restored the inactive states of target genes (Fig. 4e and Supplementary Fig. 11b). Furthermore, if EZH2T367D phospho-mimicking mutant is expressed in EZH2-depleted cells, target genes were expressed at significantly lower levels, even lower than in control cells—underscoring the importance of DCAF1-EZH2T367p for target gene inactivation in colon cancer cells (Fig. 4e and Supplementary Fig. 11b). In all cases, the mRNA levels of the non-DCAF1/EZH2 target gene *RPL23* remain unchanged.

In order to check whether the observed function of DCAF1 and EZH2 in suppressing target gene transcription reflects their localization, we next probed DCAF1 and EZH2 occupancy for the three different regions of two target genes *ARL14* and *HOXA2* by chromatin immunoprecipitation (ChIP) assays. In accordance with our previous study^5,6^, DCAF1 signals were higher at the promoter (P) and proximal coding region (CR) than at transcription start site (TSS) in mock-depleted control SW620 cells (Fig. 4f and Supplementary Fig.12a). We also found that EZH2 occupancy and EZH2T367p patterns across the target genes were similar to those observed for H3K27me3. Additionally, DCAF1 showed a pattern of accumulation indistinguishable from those of EZH2 and EZH2T367p, suggesting a major role for DCAF1 in mediating EZH2 localization and EZH2T367p at target genes. In fact, EZH2T367p ChIP signals were significantly reduced at the target genes after DCAF1 knockdown, and such changes also diminished the levels of EZH2 and H3K27me3 (Fig. 4f and Supplementary Fig. 12a). These results were further validated by rescue experiments demonstrating that ectopic expression of DCAF1 largely overrides EZH2T367p and H3K27me3 defects caused by DCAF1 knockdown (Fig. 4f and Supplementary Fig. 12a). It was also apparent in our parallel ChIP assays that ectopic expression of EZH2 wild type, but not EZH2T367A phosphorylation-blocking mutant, in EZH2-depleted cells restored H3K27me3 to levels quantitatively similar to those observed with mock-depleted control cells (Fig. 4g and Supplementary Fig. 12b). As a control, DCAF1 and EZH2 were minimally detected at the non-DCAF1/EZH2 target gene *RPL23*, and failed to show any changes in response to their knockdown and expression (Supplementary Figs. 12c, d). Collectively, these results support the notion that DCAF1 is required for the initial recruitment and stable localization of EZH2 at the target genes and that this process is tightly regulated by EZH2T367p.

### DCAF1 and EZH2 inhibitors modulate colon cancer cell growth and target gene expression

**Fig. 5:**
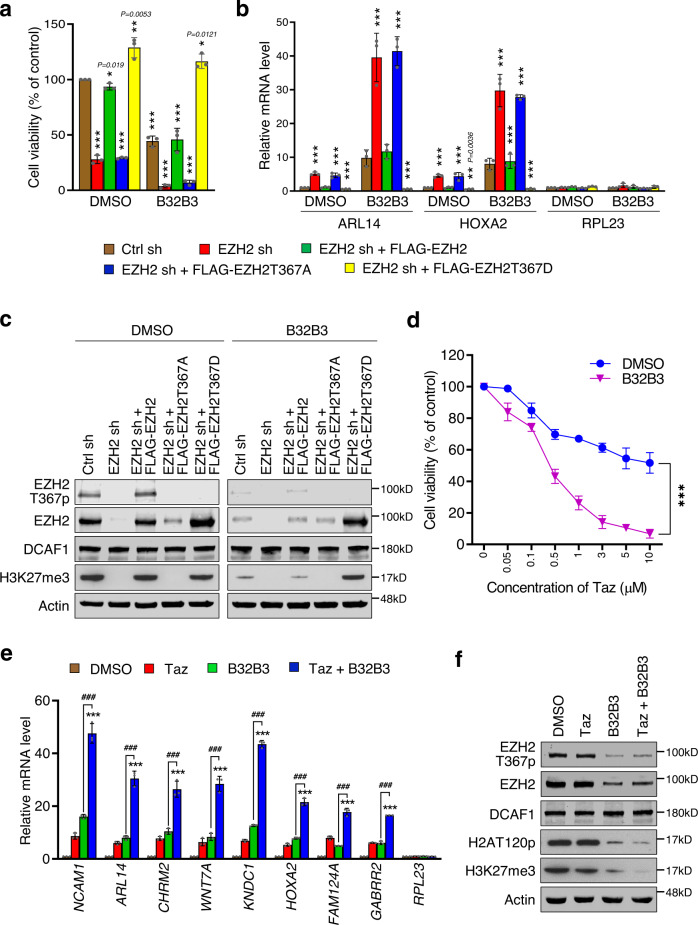
DCAF1 and EZH2 inhibitors as potent modulators of cell growth and target gene expression. **a** EZH2-depleted SW620 cells were complemented with lentiviruses expressing 3×FLAG-EZH2 wild type, T367A phospho-blocking mutant, or T367D phospho-mimicking mutant. The cells were then treated with DMSO or B32B3 (0.9 µM) for 3 days, and WST assays were performed to measure their viability. Data are represented as mean ± SEM of three independent experiments. *P* values were calculated using two-way ANOVA with post-hoc Tukey’s test for multiple comparisons. *P < 0.05, **P < 0.01 and ***P < 0.001 versus Ctrl sh. **b** After treating SW620 cells as in (**a**), total RNA was prepared and analyzed by RT-qPCR using primers listed in Supplementary Table 4. Data are represented as mean ± SEM of three independent experiments. *P* values were calculated using two-way ANOVA with post-hoc Tukey’s test for multiple comparisons. **P < 0.01 and ***P < 0.001 versus Ctrl sh. **c** After treating SW620 cells as in (**a**), whole cell lysates were prepared and subjected to Western blot analysis with the indicated antibodies. Data are representative of three independent experiments. **d** SW620 colon cancer cells were treated with increasing concentrations of Taz (0–10 µM) in the absence or presence of B32B3 (0.9 µM) for 72 h, and their viability was measured by WST assays. Data are represented as mean ± SEM of three independent experiments. *P* values were calculated using two-way ANOVA with post-hoc Tukey’s test for multiple comparisons (***P < 0.001). (See also Supplementary Fig. 13.). **e** SW620 cells were treated with IC50 concentrations of B32B3 (0.9 µM) and/or Taz (15 µM) for 72 h, and total RNA was prepared and analyzed by RT-qPCR using primers listed in Supplementary Table 4. Data are represented as mean ± SEM (*n* = 3 biologically independent experiments). *P* values were calculated using two-way ANOVA with post-hoc Tukey’s test for multiple comparisons. ***P < 0.001 versus DMSO; ###*P* < 0.001 versus B32B3. **f** SW620 cells were treated as in (**e**), and cell lysates were prepared and analyzed by Western blotting with the antibodies indicated on the left. Data are representative of three independent experiments. Source data are provided as a Source Data file.

Having established a role for DCAF1-mediated EZH2T367p in driving oncogenic gene silencing program, we next asked whether chemical inhibition of DCAF1 would affect EZH2T367p and cancer cell growth. As has been previously observed^6^, treatment of SW620 colon cancer cells with the half-maximal inhibitory concentration (IC50) of DCAF1 inhibitor B32B3 efficiently decreased cell growth rates. Interestingly, the knockdown of EZH2 impaired the growth of SW620 cells, but EZH2-depleted cells still showed the sensitivity to B32B3 treatment (Fig. 5a). Given the demonstrated reliance of DCAF1 function on H2AT120p in SW620 cells^6^, a possible explanation for this observation is that H2AT120p becomes the dominant DCAF1 target and generate additional effects of B32B3 treatment in EZH2-depleted cells. In parallel assays, B32B3 treatment of EZH2-depleted SW620 cells expressing EZH2T367A phospho-blocking mutant was not able to augment growth capacity, results indicative of the requirement of EZH2T367p for the observed action of B32B3 (Fig. 5a). Meanwhile, when EZH2-depleted SW620 cells expressing EZH2T367D phospho-mimicking mutant were treated with B32B3, there was no impact on the growth potential of SW620 cells (Fig. 5a). Since H2AT120p was almost completely abolished in B32B3-treated SW620 cells^6^, these results strongly suggest that DCAF1 can function to stimulate the growth of colon cancer cells in an EZH2T367p-dependent but H2AT120p-independent manner. In accordance with these findings, our RT-qPCR analyses demonstrated that DCAF1 inhibition with B32B3 caused a significant increase in the expression of *ARL14* and *HOXA2* genes in both control and EZH2-depleted SW620 cells (Fig. 5b). Likewise, B32B3 treatment also generated more potent activation of the target genes in EZH2-depleted cells transfected with EZH2T367A, but no such enhancement of target gene transcription was observed after treating EZH2T367D-transfected, EZH2-depleted cells with B32B3 (Fig. 5b).

To extend the above observations, we also analyzed the effects of B32B3 on EZH2 stability and EZH2T367p in SW620 colon cancer cells. In agreement with DCAF1 knockdown results, a marked decrease in EZH2T367p and EZH2 protein levels and thus H3K27me3 status was evident after treating SW620 cells with DCAF1 inhibitor B32B3 (Fig. 5c). Although EZH2-depleted SW620 cells were almost completely devoid of EZH2 and thus did not show any effects of B32B3, EZH2 protein and H3K27me3 levels were clearly reduced in EZH2-depleted cells expressing EZH2 wild type and T367A mutant in response to B32B3 treatment. Conversely, B32B3 treatment of EZH2-depleted cells transfected with EZH2T367D failed to trigger a similar decrease of EZH2 protein and H3K27me3 levels (Fig. 5c). The observed effect of B32B3 is suggestive of EZH2-targeted action of DCAF1 in colon cancer cells. We therefore next asked whether colon cancer cells are more responsive to the combination of DCAF1 inhibitor B32B3 and EZH2 inhibitor Tazemetostat (Taz). As summarized in Fig. 5d, treating SW620 colon cancer cells with increasing concentrations of Taz in the presence of B32B3 decreased cell viability more significantly. In these assays, the synergy between Taz and B32B3 was clearly detectable from our statistical calculation of combination index as a function of the fraction affected (Supplementary Fig. 13 and Supplementary Table 3). Moreover, when SW620 cells were treated with B32B3 plus Taz combination, target gene transcription was activated to much higher levels than by treatment of B32B3 or Taz alone (Fig. 5e). Congruent with these observations, H3K27me3 was enriched at the promoter and coding regions of ARL14 and HOXA2 genes in SW620 colon cancer cells but largely disappeared following B32B3 and Taz treatment (Supplementary Fig. 14a). The involvement of DCAF1-mediated T367p in governing EZH2 occupancy at target genes was also confirmed by similar ChIP assays showing a substantial decrease in the levels of EZH2 and EZH2T367p at target genes after treating with a combination of B32B3 and Taz (Supplementary Fig. 14b). Since Taz treatment alone did not appreciably alter EZH2 and EZH2T367p levels, these results support the dominant effect of B32B3 over Taz and point to DCAF1-mediated EZH2T367p as a key determinant for H3K27me3-induced oncogenic gene silencing in colon cancer. The observed effects of B32B3 and Taz were also verified by monitoring changes in EZH2 protein, EZH2T367p, and H3K27me3 levels (Fig. 5f).

### DCAF1 and EZH2 inhibitors have antitumor activity in colon cancer models

**Fig. 6:**
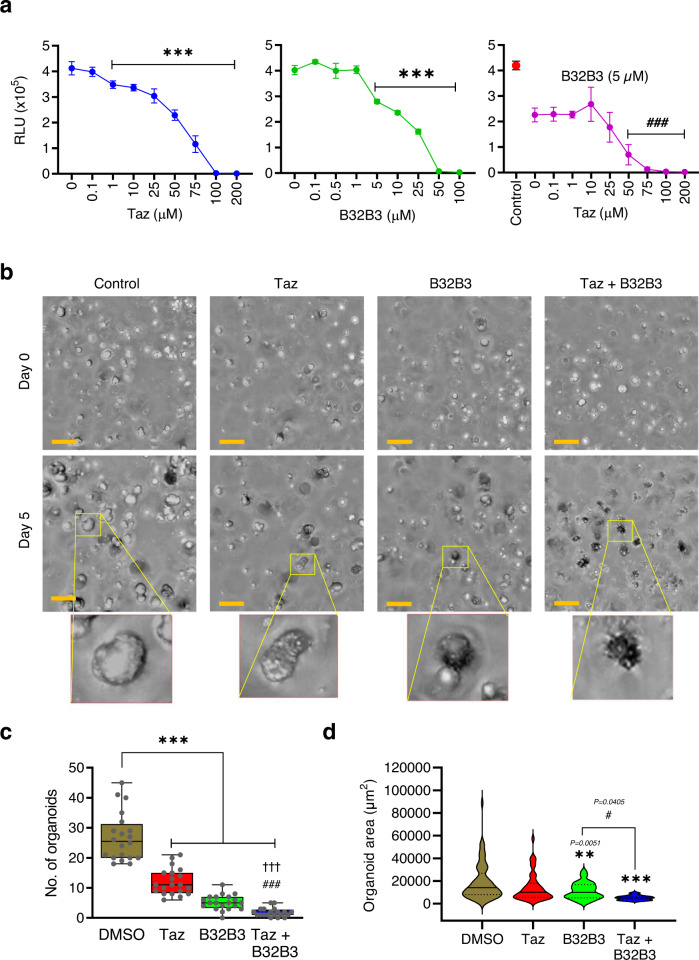
Suppressive effects of DCAF1 and EZH2 inhibitors against patient-derived tumor organoids. **a** PDOs were treated with B32B3 and/or Taz at indicated concentrations and analyzed by CellTiter-Glo (CTG) 3D luminescent cell viability assay as recently described^6^. Shown are the dose-response curves depicting the relative luminescence units for each treatment condition represented with mean ± SEM (*n* = 3) from one representative CTG assay on PDO-000V8 with *n* = 6 technical replicates. *P* values were calculated using student one-sided *t*-tests. ***P < 0.001 versus DMSO; ###*P* < 0.001 versus B32B3 (5 µM). **b** Brightfield images of PDOs were captured at day 0 and day 5 after treatment with B32B3 (5 µM) and/or Taz (50 µM) as indicated, and processed with a high-content analysis system. A representative organoid from day 5 post-treatment has been magnified within each field. Orange scale bars = 200 µm. **c** Box plot displays the counts of PDOs in (**b**). The central line in the box represents the median, and the edges of the boxes are the 25% and 75% percentile, respectively. The whiskers represent the extent of the data within 1.5 times the interquartile range. *P* values were calculated using one-way ANOVA with post-hoc Tukey’s test for multiple comparisons. ***P < 0.001 versus DMSO; †††*P* < 0.001 versus Taz; ###*P* < 0.001 versus B32B3. **d** Violin plot represents the distribution of the size of a single PDOs in (B). *P* values were calculated using one-way ANOVA with post-hoc Tukey’s test for multiple comparisons. **p < 0.01and ***P < 0.001 versus DMSO; #*P* < 0.05. (**a**, **c** and **d**) Source data are provided as a Source Data file.

As an extension of the above-described studies that established growth inhibitory effects of B32B3 and Taz on colon cancer cell lines, it was also important to evaluate B32B3 versus Taz in a more physiological context. For this objective, we chose to use colonic cancer patient-derived organoid (PDO) models that have been successfully employed in our recent study^6^. PDOs recapitulate the intra- and inter-tumor heterogeneity seen in human cancers and allow to study sub-clonal dynamics within individual tumors during progression and therapy resistance, thus being a valuable preclinical model for more accurate predictions of clinical outcome. PDOs were first generated using cell suspensions derived from digested colonic tumor specimens following the procedure described^37^. Then, PDOs were treated with increasing concentrations of B32B3 and Taz for 5 days, and their response to the treatments was determined by measuring luminescence intensity. In initial assays, treating PDOs with B32B3 and Taz individually reduced PDO growth in a dose-dependent manner (Fig. 6a, left and middle panels). Given the demonstrated reliance of EZH2 stability on DCAF1-mediated T367p and EZH2 function dependent of H3K27me3 in colon cancer cells, it was reasonable to expect greater inhibitory effects of Taz plus B32B3 combination in our assays. In fact, our luminescent cell viability analysis clearly demonstrated that Taz treatment over the concentration range of 0–200 µM in the presence of 5 µM B32B3 inhibited PDO growth more efficiently than either single inhibitor alone (Fig. 6a, right panel). Consistent with these observations, phase contrast images taken from PDO samples showed more significant changes in total number and size after Taz plus B32B3 treatment, compared with those generated by their individual treatment (Fig. 6b–d).

**Fig. 7:**
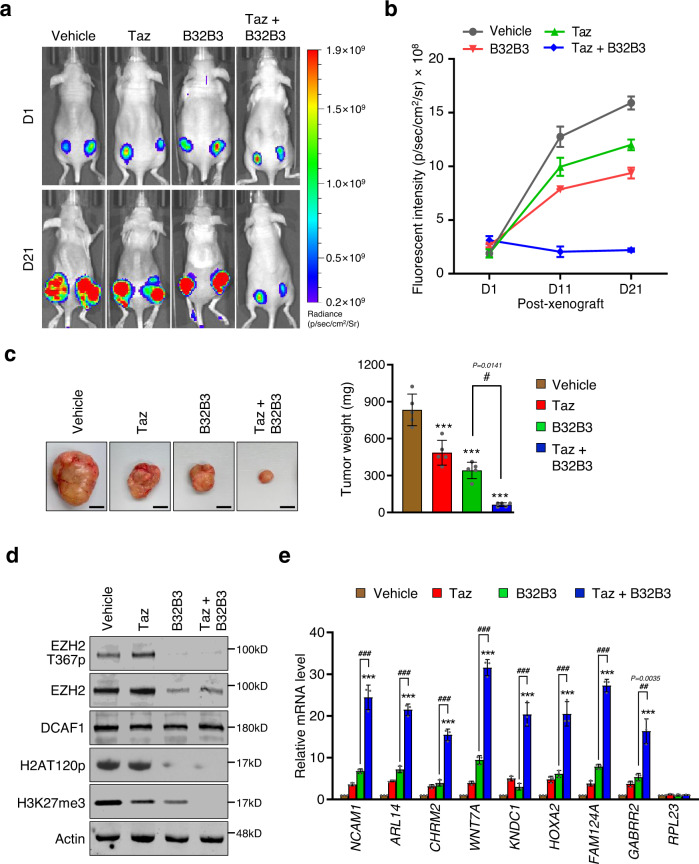
Impaired tumor growth in mice treated with DCAF1 and EZH2 inhibitors. **a** SW620 colon cancer cells were stably transfected with EGFP expression construct and subcutaneously injected into both right and left hind legs of mice. After initial tumor growth for 4 days, mice were randomized into four cohorts and treated for 21 days with intraperitoneal injection of vehicle daily, oral administration of Taz (200 mg/kg) twice daily, intraperitoneal injection of B32B3 (5 mg/kg) every three days or Taz administration plus B32B3 injection. Tumor growth was monitored by checking fluorescent signals detected at wavelengths of 581 nm with an in vivo imaging system (IVIS) after 1, 11 and 21 days of treatments. (See also Supplementary Fig. 18). **b** Fluorescence intensities were quantified using regions of interest (ROIs) of equivalent-sized areas from lower abdominal regions at the indicated time points. Data are presented as mean ± SEM (*n* = 8). **c** Mice were sacrificed at the end of 21-day treatments with Taz and/or B32B3, and subcutaneous tumors were surgically excised and photographed (left panel, scale-1 cm). The weight of tumor xenografts was also measured and expressed in milligrams (right panel). Data are presented as mean ± SEM (*n* = 8 biologically independent experiment). *P* values were calculated using one-way ANOVA with post-hoc Tukey’s test for multiple comparisons. ***P < 0.001 versus DMSO; #*P* < 0.05. **d** After 21-day treatments with Taz and/or B32B3, mice were sacrificed, and tumors were extracted and analyzed by Western blotting with the indicated antibodies. Data are representative of three independent experiments. (See also Supplementary Figs. 16a, b, left panels.) **e** Relative mRNA levels of DCAF1/EZH2 target genes in tumors extracted from Taz/B32B3-treated mice were determined by RT-qPCR. Data were represented as mean ± SEM of three independent experiments. *P* values were calculated using two-way ANOVA with post-hoc Tukey’s test for multiple comparisons. **P < 0.01 and ***P < 0.001 versus DMSO; ##*P* < 0.01 and ###*P* < 0.001 versus B32B3. (See also Supplementary Figs. 16a, b, right panels) (**b**–**e**) Source data are provided as a Source data file.

To further evaluate the effects of Taz and B32B3 in vivo, we generated xenograft models by subcutaneously injecting SW620 cells expressing enhanced green fluorescent protein (eGFP) into both flanks of nude mice. When Taz and B32B3 were individually administered to SW620 xenograft mice over 21 days, they were able to impair the proliferative ability of SW620 xenograft tumors (Fig. 7a, b). We then tested Taz and B32B3 combination by administering Taz plus B32B3 to mice according to the protocol in Supplementary Fig. 15. Mirroring the results in PDOs (Fig. 6), Taz plus B32B3 treatment caused a more significant inhibition in the growth of SW620 xenograft tumors up to 70-80% compared to those treated with a single agent, either Taz or B32B3 (Fig. 7c). In addition, the ability of the co-treatments to maintain the similar mechanism of action in vivo was investigated by Western blot analysis of EZH2T367p status in three representative SW620 xenograft tumors. Results showed that Taz plus B32B3 treatment inhibits EZH2T367p by 60–85% compared to vehicle-treated animals (Fig. 7d and Supplementary Fig. 16). Thus, the substantially reduced tumor growth seen in the mice treated with Taz plus B32B3 compared to the mice treated with Taz or B32B3 alone indicates that Taz in the combination treatment impaired the residual tumorigenic activities of EZH2 in the xenograft tumors. Considering the role of EZH2T367p in enhancing EZH2 protein stability, we also examined the effects of Taz and B32B3 on EZH2 protein levels in SW620 xenograft tumors explanted from mice. As shown in Fig. 7d and Supplementary Fig. 16, B32B3 treatment generated about 70% reduction in EZH2 protein levels, which is coupled with a substantial decrease in EZH2T367p. However, unlike treatment with B32B3, we failed to detect any reduction of EZH2T367p in Taz-treated mice (Fig. 7d and Supplementary Fig. 16). Consistent with these findings, immunostaining of xenograft tumors confirmed lower levels of EZH2 in B32B3-treated mice, whereas the levels of DCAF1 were not decreased in Taz-treated mice (Supplementary Fig. 17). As an experiment to elucidate the mechanistic basis of B32B3/Taz effects, our RT-qPCR data also showed that target genes were more highly expressed after treating mice with Taz plus B32B3 compared with Taz or B32B3 alone (Fig. 7e and Supplementary Fig. 16). Importantly, Taz and B32B3 were well tolerated both alone and in combination, with no body weight loss observed in the SW620 xenograft model (Supplementary Fig. 18), suggesting that they may be used for effective colon cancer therapy.

## Discussion

DCAF1 is overexpressed in colon cancer and catalyzes H2AT120p to inactivate genes encoding master regulators of cell growth and proliferation;^6^ however the fact that many histone modifying enzymes also target non-histone proteins raises the possibility of additional DCAF1 functions mediated via non-histone substrates. Thus, investigating the function of DCAF1 in non-histone modifications should provide greater insights into oncogenic signaling pathways and more effective strategies for tackling colon and other cancers. The data presented here now demonstrate the role of DCAF1 in mediating EZH2T367p during the development of colon cancer. DCAF1-mediated EZH2T367p stimulates cancer cell growth via an accumulation of EZH2 protein and an activation of EZH2 enzymatic activity catalyzing H3K27me3. Inactivation of growth regulatory genes by H3K27me3 follows shortly, resulting in uncontrolled cell proliferation and growth. Our observation of DCAF1-dependent phosphorylation and stabilization of EZH2 at growth regulatory genes suggests a concerted mechanism for the placing of EZH2T367p and H3K27me3 marks in colon cancer cells. The requirement of T367p for EZH2 stability clearly allows for very tight regulation of EZH2 protein levels. Given the important role that EZH2 plays in controlling H3K27me3-induced cell growth and cancer development^16–18,38–40^, it is not surprising that such a tightly regulated mechanism has evolved to control the oncogenic function of EZH2. In further support of these data, DCAF1 expression is significantly upregulated in colon cancer patient samples, and this phenomenon correlates well with EZH2T367p levels. Thus, our study not only identifies EZH2T367p as a biomarker for the prediction of colon cancer but also provides the evidence that targeting DCAF1 kinase activity toward EZH2 is a promising strategy for tackling colon cancer development.

Along with our previous discovery of DCAF1-mediated H2AT120p, the findings described here also highlight a tight coupling between EZH2T367p and H3K27me3 in DCAF1-induced epigenetic gene silencing in colon cancer cells. Mechanistically, DCAF1-mediated EZH2T367p modulates the nature and strength of EZH2 interaction with other components of PRC2 complex thereby increasing its stability and HMT activity toward H3K27. Since T367 is located on the unstructured surface loop of EZH2, DCAF1-mediated EZH2T367p may cause a long-range conformational change through allosteric modulation and positively affect the interaction of EZH2 with other PRC2 subunits as shown in our binding assays. These findings indicate another case of coupling histone modification and non-histone modification for the regulation of gene transcription, and the use of two different modifications would make such regulatory system more robust. Our molecular analysis in colon cancer cells strongly supports these findings and further argues that such interactions may be an integral component of oncogenic transcriptional inactivation in colon cancer cells. Related to these results, a recent study showed that p38 phosphorylates EZH2T367 and potentiates EZH2 cytoplasmic localization and action as a mediator of metastatic progression of breast cancer^22^. However, we found that p38 is expressed at very low levels and distributed throughout the nucleus and cytoplasm in the colon cancer cell lines that we used in our study (Supplementary Figs. 5a, 19a). Also, knockdown of p38 and treatment with the p38 specific inhibitor SB202190 failed to generate any detectable changes in the levels of EZH2T367p and EZH2 in colon cancer cells (Supplementary Figs. 5a, 19b). We thus speculate that the role of DCAF1-mediated EZH2T367p in colon cancer is distinct from that of p38-mediated EZH2T367p in breast cancer. Further biological and functional analyses of DCAF1 and p38 would be helpful for understanding the cell type specific roles that these two kinases play.

In our study, we also found that treatment of colon cancer cells with the selective DCAF1 inhibitor B32B3 reactivates growth regulatory genes and inhibits uncontrolled cell growth. We believe that this is not merely due to its inhibitory effects on H2AT120p, as the doses used in this study failed to suppress the growth capacity of EZH2-depleted cells transfected with phospho-mimicking EZH2T367D mutant. Blocking DCAF1-mediated EZH2T367p is another critical process for cancer cell response to the DCAF1 inhibitor B32B3. In an extension of this initial discovery, we also demonstrated that treating colon cancer cells with subtoxic concentrations of EZH2 inhibitor Taz recapitulates the effects of DCAF1 inhibitor albeit somewhat less efficiently (Supplementary Fig. 20). Further, Taz and B32B3, when combined, synergistically stimulate the expression of target genes and prevent the growth of colon cancer cells. These results have important implications of using Taz and B32B3 as a combination therapy strategy against colon tumors harboring high levels of EZH2T367p and H3K27me3. In support of this idea, our study proved in vivo efficacy of Taz plus B32B3 combination superior to either inhibitor alone in SW620 colon tumor xenograft models, without affecting body weight of the animals. The fact that dual DCAF1 and EZH2 inhibition by Taz plus B32B3 combination has minimal effects on the growth of NCM460 normal colon cells is also in line with our observation of DCAF1 and EZH2 expression being largely undetectable in this normal colon cell line (Fig. 1e). This is an important finding as it suggests that DCAF1 and EZH2 inhibitors may be combined to achieve a more favorable therapeutic index for colon cancer patients by establishing distinct epigenetic states at growth regulatory genes.

Although the present study revealed a critical role for DCAF1-mediated EZH2T367p in colonic tumorigenesis, whether the observed function of DCAF1 also requires H2AT120p remains unclear. However, of particular interest in this regard is the observation that EZH2T367D phospho-mimicking mutant is capable of fully restoring the original growth rates of DCAF1-depleted colon cancer cells (Supplementary Fig. 4b). Given that the mimicking mutant alone is also sufficient to inactivate growth regulatory genes in DCAF1-depleted cancer cells (Supplementary Fig. 4b), DCAF1 seems to rely more heavily on EZH2T367p rather than on H2AT120p in driving colonic tumorigenesis. Such interpretation is further supported by our assays showing that B32B3 treatment is ineffective at impairing cancer cell growth and H3K27me3 after ectopic expression of EZH2T367D phospho-mimicking mutant in EZH2-depleted colon cancer cells (Fig. 5a). Currently, we do not know the potential mechanism behind the possible selectivity of EZH2T367p over H2AT120p for DCAF1 function in colon cancer cells, but our observation highlights the importance of EZH2T367p in DCAF1-induced oncogenic gene silencing and colonic tumorigenesis. In the future, it will be interesting to determine what degree DCAF1 oncogenic activity is dependent on EZH2T367p and H2AT120p in different types of cancer, and how those two epigenetic processes may underlie a key mechanism during uncontrolled cell proliferation and rapid cell growth.

Based on our observations, together with findings from our previous studies, we present a working model for how DCAF1 functionally cooperates with EZH2 to inactivate growth regulatory genes (Supplementary Fig. 21). DCAF1 is highly expressed in colon cancer cells and catalyzes EZH2T367p. This modification then participates in creating EZH2 structure that allows a more favorable interaction with EED and SUZ12 to form PRC2 core complex. This enhances the stability and HMT activity of EZH2 and initiates an aberrant epigenetic silencing process involving H3K27me3 to keep target genes in an inactive state. In this scenario, DCAF1-mediated EZH2T367p can be viewed as a critical oncogenic process, such that regulating this process could be a strategy for reactivating growth regulatory genes and suppressing pathological cell growth.

## Methods

### Cell lines, constructs, and antibodies

Five colon cancer cell lines (HCT116, HCT15, HT29, RKO, LOVO), two colon cancer cell lines (SW1222 and LS174T), two colon cancer cell lines (T84 and SW480) and one normal colon cell line (NCM460) were provided from Dr. Gangning Liang, University of Southern California (Los Angeles, CA, USA), Dr. Steven M. Larson, Memorial Sloan Kettering Cancer Center (New York, NY, USA), Dr. Adam E. Snook, Thomas Jefferson University (Philadelphia, PA, USA), and Dr. Charalabos Pothoulakis, UCLA Center for Inflammatory Bowel Diseases (Los Angeles, CA, USA), respectively. 293 T (CRL-3216), SW620 (CCL-227) and Caco2 (HTB-37) were obtained from ATCC (American Type Culture Collection, Manassas, VA, USA). HCT116, HCT15, HT29, SW620, SW480, SW1222, RKO, Caco2, LS174T and T84 cells were cultured in Dulbecco’s modified Eagle’s medium. MDA-MB231, LOVO and NCM460 cells were cultured in RPMI1640 medium. Both media were supplemented with 10% fetal bovine serum in an atmosphere of 5% CO_2_ at 37 °C. For mammalian expression of DCAF1, EZH2 and H2A, the corresponding cDNAs were amplified by PCR and ligated into the lentiviral expression vector pLenti-Hygro (Addgene, Cambridge, MA, USA) containing 5’ 3×FLAG coding sequence. DCAF1 cDNA was amplified by PCR and inserted into the EcoRI and XhoI sites of pFASTBAC vector with an N-terminal 6×His tag to generate DCAF1 baculovirus expression system^5^. For bacterial expression of EZH2, SUZ12 and EED, their cDNAs were subcloned into the pET15b vector in frame with 5’ 6x His tag and pET11d vector with a 5’ FLAG tag. To generate mutant EZH2 and H2A expression vectors, wildtype EZH2 and H2A cDNAs were mutated by using Q5 Site-Directed Mutagenesis Kit (New England Biolabs, Ipswich, MA, USA) after the construction. All constructs were verified by DNA sequencing. Antibodies used in this study are as follows: anti-FLAG antibody (1:1000) from Sigma-Aldrich, St. Louis, MO, USA; anti-actin (1:10000), anti-HA (1:5000), anti-His (1:5000), anti-VprBP (1:1000), anti-EZH2 (1:500), anti-EED (1:500) and anti-SUZ12 (1:1000) antibodies from Proteintech, Rosemont, IL, USA; and anti-H3K27me3 (1:2000), anti-H3S10p (1:5000), anti-H2BS14p (1:5000), anti-H3ac (1:2000), anti-H4ac (1:1000) antibodies from MilliporeSigma, Burlington, MA, USA; and anti-H2AT120p (1:2500) and anti-H3K4me3 (1:500) antibodies from Active Motif, Carlsbad, CA, USA; anti-H3K9me3 (1:1000), H3K36me3 (1:1000), H3Y41p (1:1000) and anti-H3K79me3 (1:1000) antibodies from abcam, Cambridge, MA, USA; anti-rabbit (1:5000), anti-mouse (1:2000) secondary antibodies from Thermo Fisher Scientific, Waltham, MA, USA.

### Generation of anti-EZH2T367p antibody

A polyclonal antibody against EZH2T367p was generated by ABclonal Biocechnology (Wuhan, Hubei, China) with New Zealand rabbits using the synthetic C-SSRPSTpPTIN peptide as an immunogen. After boosting rabbits over 90 days, blood was collected and passed three times through a non-phosphorylated peptide column to remove antibody that recognizes the peptide without phosphorylation. The final flowthrough was loaded on a phosphorylated peptide column, and bound EZH2T367p antibody was eluted with 0.1 M glycine (pH 2.5) and neutralized with 1 M Tris buffer. To validate purified antibodies, serial dilutions of non-phosphorylated or phosphorylated peptides were spotted on a polyvinylidene difluoride (PVDF) membrane and blotted with the EZH2T367p antibody. For the peptide competition, the antibodies were preincubated with non-phosphorylated or phosphorylated peptides at room temperature for 60 min before immunoblotting.

### Tissue samples

10 colon tumor specimens along with 10 normal tissue counterparts were obtained from the Division of Medical Oncology, Norris Comprehensive Cancer Center at University of Southern California, USA. Western blot analyses of DCAF1 and EZH2 expression were performed on fresh frozen specimens. The study was approved by University of Southern California’s Ethics Committee and all patients provided written informed consent according to the committee’s regulations.

### Histone extraction and nucleosome purification

Histones were purified from SW620 cells according to the acid extraction method as detailed previously^41^. To purify nucleosomes containing H2A wild type or T120A mutant, SW620 colon cancer cells were infected with lentiviruses expressing H2A wild type or T120A mutant containing an N-terminal 3×FLAG tag. The cell lines were selected for two weeks in the presence of hygromycin (800 μg/ml). Total nucleosomes were prepared using Nucleosome Preparation Kit (Active Motif, Carlsbad, CA, USA) and ectopic H2A-containing mononucleosomes were isolated by immunoprecipitation using anti-FLAG M2 agarose beads in washing buffer (20 mM HEPES, pH 7.8, 300 mM NaCl, 1.5 mM MgCl_2_, 0.2 mM EGTA, 10% glycerol, 0.2% Triton X-100, and protease inhibitor cocktail). Levels of H3K27me3 of bead-bound nucleosomes were analyzed by Western blotting. All experiments were performed in triplicates.

### In vitro kinase assay

For in vitro kinase assays, 6×His-tagged EZH2 and DCAF1 were expressed in *E. coli* Rosetta 2 (DE3) pLysS cells and Sf21 insect cells, respectively, and purified with Nickel beads (MilliporeSigma) as detailed previously^42,43^. FLAG-tagged EZH2 was expressed in *E. coli* Rosetta 2 (DE3) pLysS cells and purified with anti-FLAG M2 agarose (Sigma-Aldrich) according to manufacturer’s instructions. In vitro phosphorylation assays were performed with recombinant EZH2 and DCAF1 in kinase buffer (50 mM Tris-HCl [pH 7.5], 20 mM EGTA, 10 mM MgCl_2_, 1 mM DTT, and 1 mM β-glycerophosphate) containing 10 µCi of [γ-^32^P] ATP and 4 mM ATP as recently described^5^. Following incubation at 30 °C for 30 min, EZH2 and DCAF1 proteins from each reaction were separated by 5–20% SDS-PAGE, and phosphorylated EZH2 proteins were visualized by autoradiography, and all assays were conducted in triplicates.

### RT-qPCR

Total RNA was isolated from SW620 and Caco2 colon cancer cells using a RNeasy Mini kit (Qiagen, Hilden, Germany) and converted to first-strand cDNA using the SuperScript III First-Strand System Kit (Thermo Scientific, Waltham, MA, USA). Real-time RT-PCR was carried out with SYBR Green Real-time PCR Master Mixes (Thermo Scientific) using Agilent Aria 1.71 software according to the manufacturer’s protocol. The primers used for RT-qPCR are listed in Supplementary Table 4. All reactions were run in triplicate, and results were normalized to β-actin mRNA levels.

### ChIP-qPCR

Chromatin immunoprecipitation (ChIP) assays with SW620 cells were performed using the ChIP Assay Kit (Millipore) as recently described^44^. After reversing the protein–DNA cross-links, immunoprecipitated DNA was purified and analyzed by quantitative real-time PCR (qPCR) using the primers that amplify the promoter region (P), transcription start site (TSS), and coding (CR) region of *ARL14*, *HOXA2* and *RPL23* genes (Supplementary Table 5). Specificity of amplification was determined by melting curve analysis, and all samples were run in triplicate.

### Circular dichroism

Circular dichroism (CD) measurements were carried out with EZH2 wild type and phosphorylation mimicking T367D mutant proteins at 5 µM concentrations in measurement buffer (20 mM K_2_HPO_4_/KH_2_PO_4_, pH 7.4, 25 mM KCl and 100 µM TCEP) using a JASCO J-810 spectropolarimeter (Tokyo, Japan). A 0.1 cm pathlength quartz cell (Hellma Analytics, Müllheim, Germany) was used to record spectra in the wavelength range of 190–260 nm. The mean residue ellipticity, Ɵ_MRW_, was interpreted in terms of the secondary structure content using the program Contin-LL^45^.

### RNA interference

DNA oligonucleotides encoding shRNAs specific for DCAF1 (CGAGAAACTGAGTCAAATGAA) and EZH2 (TATGATGGTTAACGGTGATCA) coding region were annealed and ligated into the lentiviral expression vector pLKO.1 (Addgene). Lentiviral particles were generated in 293 T cells by transfecting plasmids encoding VSV-G, NL-BH, and the shRNA. Two days after transfection, the soups containing viruses were collected and used to infect SW620 and Caco2 colon cancer cells in the presence of polybrene (8 μg/ml). Cells with stable integration of shRNA constructs were selected for two weeks in the presence of puromycin (2 μg/ml). For rescue experiments, DCAF1 and EZH2-depleted cells were infected with lentiviruses expressing shRNA-resistant DCAF1 and EZH2, and selected for two weeks in the presence of hygromycin (800 μg/ml for SW620 cells and 200 μg/ml for Caco2 cells).

### Cell viability assay

Cell viability was quantified using the WST-1 Cell Proliferation Reagent (Roche Diagnostics, Switzerland) as recently described^6^ and according to the manufacturer’s protocol. In this assay, 10 µl of WST-1 assay solution was added to each well of 96-well plate containing 100 µl of SW620 or Caco2 colon cancer cells in the culture. After 4 h incubation at 37 °C, the relative cell viabilities were given by determining the absorbance at 460 nm at each well using a microplate reader with CLARIOstar 3.41 software. Each experiment was performed in triplicate.

### Protein–protein interactions

For in vitro pull-down assays, His-tagged wild type/mutant EZH2 proteins (1 µg) were immobilized on Ni-NTA beads and incubated with FLAG-SUZ12 (1 µg) and FLAG-EED (1 µg) in 300 µl of binding buffer (20 mM HEPES-KOH, pH 7.9, 0.5 mM EDTA, 200 mM NaCl, 1 mM dithiothreitol, 10% glycerol, and 0.1% Nonidet P-40 and protease inhibitor cocktail) for 16 h at 4 °C with gentle rotation. After washing beads three times with washing buffer (20 mM HEPES-KOH, pH 7.9, 0.5 mM EDTA, 250 mM NaCl, 1 mM dithiothreitol, 10% glycerol, and 0.1% Nonidet P-40 and protease inhibitor cocktail), bound SUZ12/EED proteins were detected by Western blotting with anti-FLAG antibody. For co-immunoprecipitation assays, SW620 colon cancer cells were transfected with HA-tagged wild type/mutant EZH2 expression vectors. Two days after transfection, whole-cell lysates were prepared from cells in lysis buffer (50 mM Tris/HCl, pH 7.4, 150 mM NaCl, 1 mM EDTA, 1% Triton X100, and protease inhibitor cocktail) and subjected to anti-HA immunoprecipitation. The binding of endogenous SUZ12 and EED to ectopic EZH2 was determined by Western blotting and image data were captured using Image Studio 5.x. All experiments were repeated at least three times.

### RNA-seq

Total RNA was extracted from mock-depleted, DCAF1-depleted, or EZH2-depleted SW620 cells using the RNeasy® mini kit (Qiagen Inc., CA, USA). Three biological replicates were performed for each studied condition. The libraries were prepared and sequenced, and RNA-seq reads were mapped to hg38 GENCODE version 29 (PMID: 30357393)^46^ using STAR 2.6.1d as described in our recent study^6^. Aligned reads were quantified at gene levels, and gene counts were normalized using the upper quartile normalization method. After principal components analysis with normalized gene counts, differentially expressed genes were selected by using the Gene Specific Algorithm from Partek® Flow® software (Partek Inc., MO, USA). Fold change >1.5 and false discovery rate cut-off of 0.05 were used to identify genes that have a statistically significant difference in average expression across distinct biological groups. The lists of differentially expressed genes (DEGs) from DCAF1 and EZH2-depleted SW620 cells were analyzed to find the overlapped differentially expressed genes. The quality control (QC) metrics of RNA-seq data were also evaluated for each sample. Gene ontology and pathway analysis of common genes was performed using Ingenuity Pathway Analysis tool (IPA® version 52912811) (Qiagen Inc., CA, USA). The heat map was generated by calculating *Z* score of gene expression levels using the Generalized Minimum Distance R package heatmap.3 function^47^.

### Patient-derived tumor organoids

Colonic tumor tissues were received from consented patients following Institutional Review Board (IRB) approval at the Norris Comprehensive Cancer Center of the University of Southern California, Los Angeles, CA, USA. Colonic tumor specimens were isolated from colon cancer patients and processed mechanically and enzymatically to obtain colon cancer cells without contaminating non-cancer cells as previously described^37^. Patient-derived organoids (PDOs) were generated from these colon cancer cells as described in our recent publication^6^ and treated with B32B3 and Taz individually or in combination for 5 days. At the end of treatment, 100 μl of CellTiter-Glo 3D solution (Promega, WI, USA) was added to each well, and cell viability was analyzed by measuring luminescence with BioTek Synergy Neo2 Multi-Mode Reader (Agilent Technologies, CA, USA). Images of PDOs were also acquired 0 and 5 days after inhibitor treatments using the Operetta CLS high-content analysis system (PerkinElmer, MA, USA) and displayed with maximum intensity projections of 24 z-stacks ranging from 10–470 µm in increments of 20 µm.

### Mice xenograft

8-week-old athymic male nude mice [(Crl:NU(NCr))-Foxn1nu] were obtained from Charles River Laboratories (Wilmington, MA, USA). Mice were acclimatized under a constant light/dark cycle at 22 ± 2 °C (12 h light / dark cycles) and served with a general laboratory diet and water *ad libitum*. All mouse experiments were performed according to protocols approved by the Institutional Animal Care and Use Committee. EGFP expressing SW620 cells (1 × 10^7^) were subcutaneously injected into the left and right hind legs of mice. Four days postinjection, 32 inoculated mice were subdivided into four groups of eight mice each and treated with Taz and B32B3 for 21 days following the procedure illustrated in Supplementary Fig. 20. The first group (*n* = 8) was left untreated (Vehicle), the second group (*n* = 8) was orally administered with Taz (200 mg/kg b.w.) twice daily, the third group (*n* = 8) was intraperitoneally treated with B32B3 (5 mg/kg b.w.) every three days, and the fourth group (*n* = 8) was treated with both Taz and B32B3. Fluorescent signals from xenograft tumors were analyzed at a wavelength of 581 nm with an in vivo imaging system (IVIS) after 1, 11, and 21 days of treatment. The treatment ended once Vehicle group reached the humane endpoint of 15 mm the maximum tumor size at largest diameter. At the end point of treatment, mice were sacrificed by asphyxiation with CO_2_, and SW620 tumor xenografts were excised, photographed, and weighed. To determine the levels of EZH2T367p, DCAF1 and EZH2, lysates were also prepared from the excised xenografts and analyzed by Western blotting. Sections (5 mm) of formalin-fixed and paraffin-embedded SW620 tumor xenografts were also subject to immunofluorescence analysis by treating with blocking reagent (50 mm Tris/HCl, pH 7.5, 150 mm NaCl, 0.3% Triton X-100, and 5% normal goat serum) for 30 min at room temperature and incubating with EZH2 and VprBP antibodies overnight. FITC-tagged (1:2000) and Alexa Fluor 594-tagged (1:500) secondary antibodies were used to detect EZH2 and VprBP antibodies, respectively, in our assays.

### Functional enrichment analysis and visualization

Gene ontology (GO) and Reactome pathway analyses were conducted using clusterProfiler^48^ and ReactomePA^49^ packages in the R software (ver. 4.2.2). A value of *p* < 0.05 was considered as significant criteria. The functional enrichment pathway was visualized by Cytoscape (ver. 3.9.1)^50^ application EnrichmentMap (ver. 3.3.5)^51,52^. The network was annotated by auto-annotation of cluster by Cytoscape plug-in AutoAnnotate (ver. 1.4.0). The list of genes was filtered by an FDR cutoff of <0.05 and *p*-Value threshold of 0.05. The post-analysis function in EnrichmentMap application was used to further access a possible relationship of our functional enrichment map with known apoptotic signature genes. For this analysis, two apoptosis signatures, intrinsic (GO:0097190) and extrinsic apoptosis gene sets (REACT_1059), were obtained from MSigDB^53^, and the overlap with these gene sets were scored using Fisher’s Exact Test (*p*-Value <0.05).

### Statistical analysis

The statistical analysis was performed by KyPlot version 6.0. The IC50 values were calculated by the formula, Y  =  100*A1/(X + A1) where A1  =   IC50, Y  =  response (Y  =  100% when X  =  0), X  =  inhibitory concentration. The IC50 values were compared by one-way analysis of variance (ANOVA) with post-hoc Tukey’s test for multiple comparison. All data were presented as mean ± standard error of the mean (SEM). *P* < 0.05 was considered significant.

### Reporting summary

Further information on research design is available in the Nature Portfolio Reporting Summary linked to this article.

## Supplementary information

Supplementary Information

Reporting Summary

## Data Availability

The data that support the findings of this study are available in NCBI’s Gene Expression Omnibus and are accessible through following links: https://www.ncbi.nlm.nih.gov/geo/query/acc.cgi?acc=GSE180282, GEO Series accession number [GSE180282] for RNA-seq study performed in Ctrl sh and DCAF1 sh SW620 cells and https://www.ncbi.nlm.nih.gov/geo/query/acc.cgi?acc=GSE192489, GEO Series accession number [GSE192489] for RNA-seq study performed in EZH2 sh SW620 cells. The human reference genome hg38 GENCODE version 29 (PMID: 30357393) used in this study is available to download from https://www.gencodegenes.org/human/release_29.html. The remaining data are available in the Article, Supplementary Information and Source data. Source data are provided with this paper.

## Source data

Source Data
