# Supplementary material for: Phosphorylation and stabilization of EZH2 by DCAF1/VprBP trigger aberrant gene silencing in colon cancer
Source: Nat Commun. 2023 Apr 17;14:2140. doi: 10.1038/s41467-023-37883-1 (PMC10110550; doi:10.1038/s41467-023-37883-1)
Supplement: Supplementary file 1 — Supplementary Information [file 41467_2023_37883_MOESM1_ESM.pdf]

SUPPLEMENTARY INFORMATION

Phosphorylation and stabilization of EZH2 by DCAF1/VprBP trigger aberrant gene silencing in colon cancer

Nikhil B. Ghate[1,5], Sungmin Kim[1,5], Yonghwan Shin[1], Jinman Kim[1], Michael Doche[2], Scott Valena[2], Alan Situ[3], Sangnam Kim[1], Suhn K. Rhie[1], Heinz-Josef Lenz[4], Tobias S. Ulmer[3], Shannon M. Mumenthaler[2], and Woojin An[1]*

[1]Department of Biochemistry and Molecular Medicine, Norris Comprehensive Cancer Center, University of Southern California, Los Angeles, CA 90033, USA.
[2]Lawrence J. Ellison Institute for Transformative Medicine, University of Southern California, Los Angeles, CA 90064, USA.
[3]Department of Biochemistry and Molecular Medicine, Zilkha Neurogenetic Institute, University of Southern California, Los Angeles, CA 90033, USA.
[4]Division of Medical Oncology, Norris Comprehensive Cancer Center, University of Southern California, Los Angeles, CA 90033, USA.
[5]These authors contributed equally: Nikhil B. Ghate, Sungmin Kim.
*Correspondence : woojinan@usc.edu

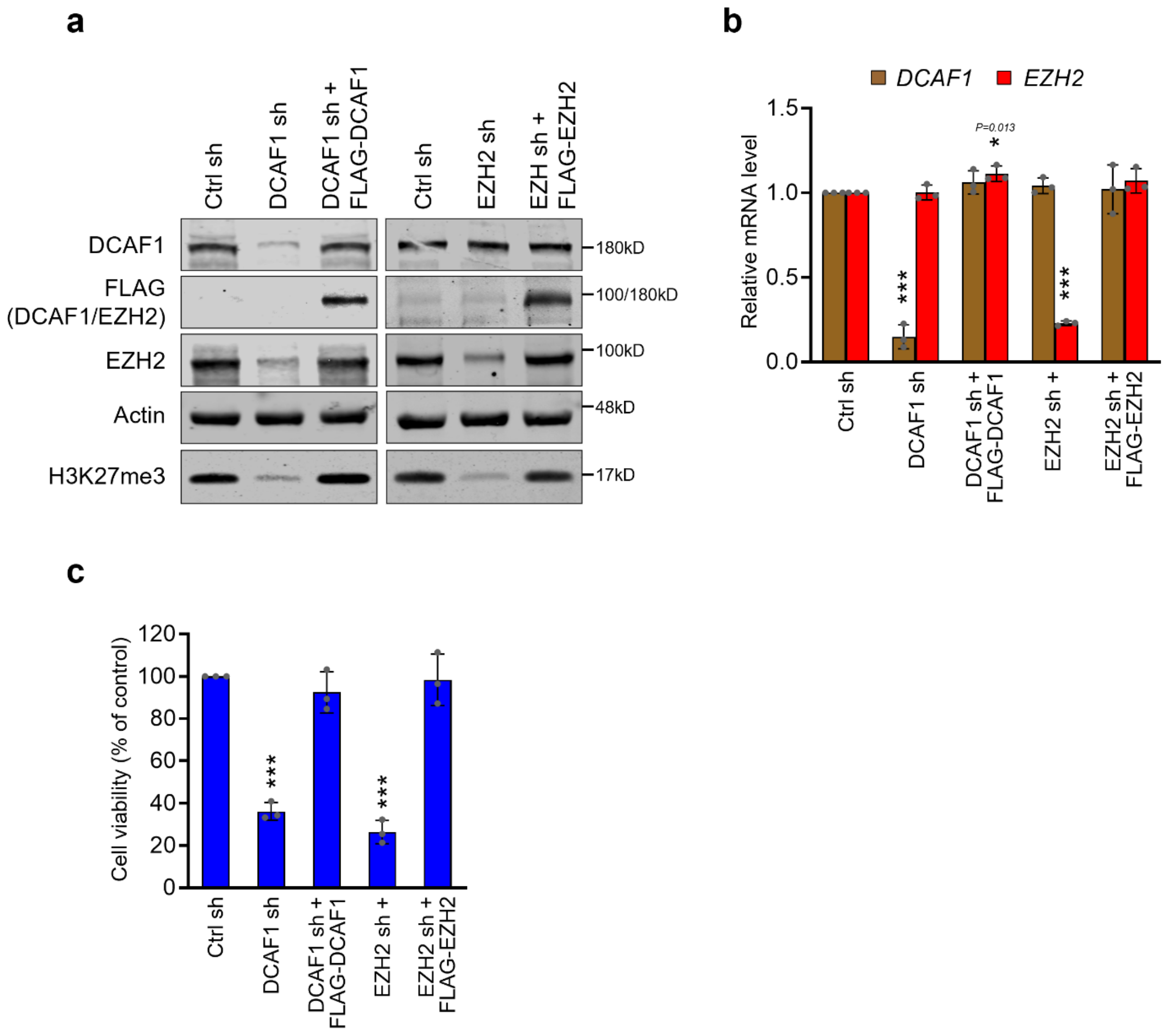

Supplementary Fig. 1 Effects of DCAF1 and EZH2 knockdown in Caco2 cells. a, Whole cell lysates were prepared from Caco2 cells depleted of DCAF1 or EZH2 and analyzed by Western blotting with the antibodies indicated on the left. The effects of DCAF1 and EZH2 knockdown were also rescued by expression of 3×FLAG-DCAF1 and 3×FLAG-EZH2. Shown are the representative results of three independent immunoblot experiments. **b,** Caco2 cells were depleted and transfected as in (a), and total RNA was extracted and subjected to RT-qPCR analysis. Data are represented as mean ± standard error of the mean (SEM) (n = 3 biologically independent experiments). *P* values were calculated using two-way analysis of variance (ANOVA). *$P < 0.05$ and ***$P < 0.001$ versus Ctrl sh. **c,** DCAF1- or EZH2-depleted Caco2 cells were rescued as in (a), and their growth rates were quantified after 72 h culture using the cell proliferation reagent WST-1 reagent. Data are shown as mean ± SEM (n = 3 biologically independent experiments). *P* values were calculated using one-way ANOVA test. ***$P < 0.001$ versus Ctrl sh. Source data are provided as a Source Data file.

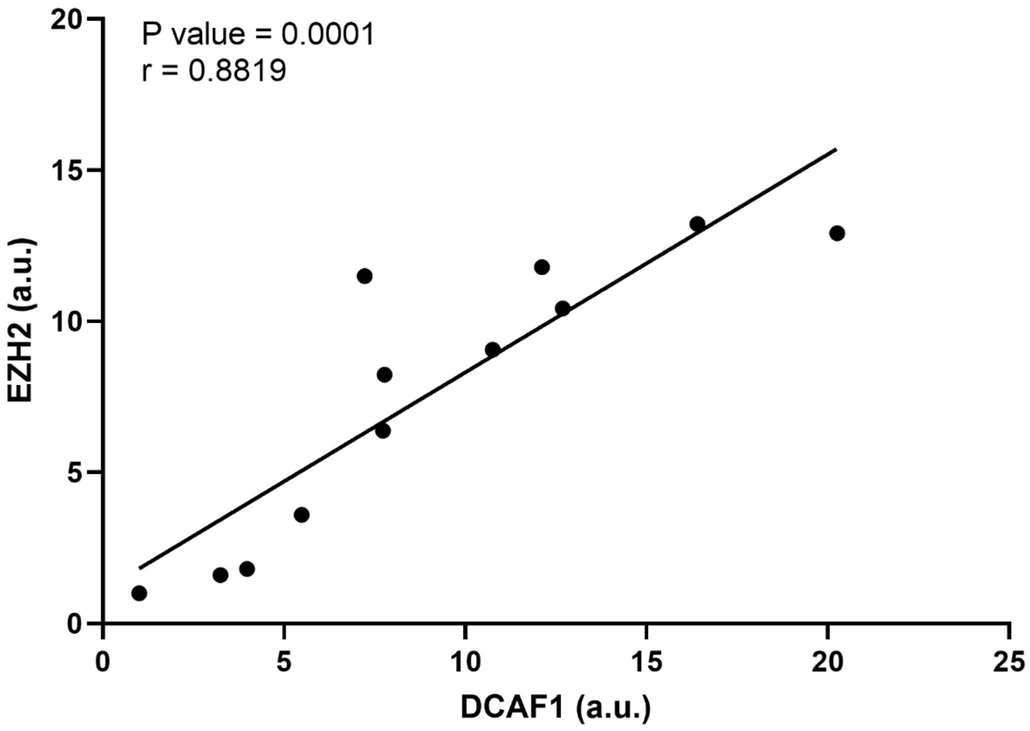

Supplementary Fig. 2 Correlation between EZH2 and DCAF1 protein levels in colon cancer cell lines. Pearson's correlation coefficients (r) were calculated between EZH2 and DCAF1 protein levels shown in Figure 1E, and a dot of scatter plot represents the individual score of each of protein abundance. P value is determined by two-sided Pearson's correlation test using GraphPad Prism 9 software. Source data are provided as a Source Data file.

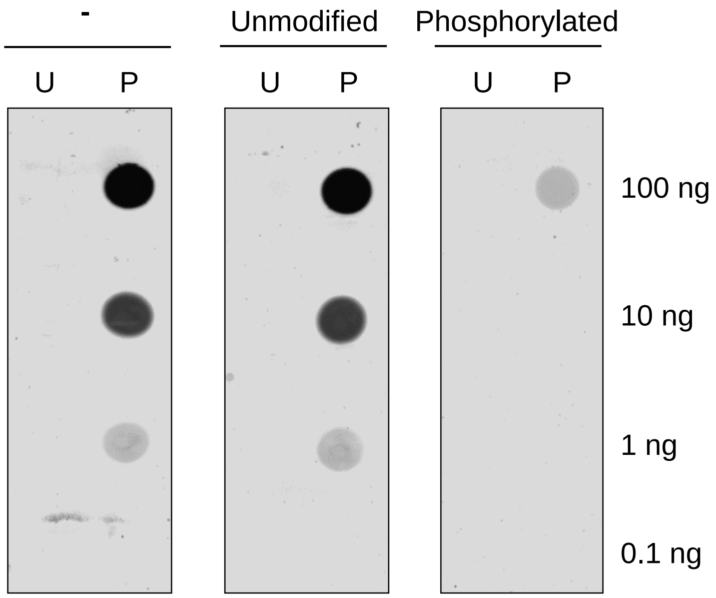

Supplementary Fig. 3 Generation of EZH2 T367p-specific antibody. Dot blotting of unmodified (U) and phosphorylated (P) peptides was performed in the presence of either unmodified or phosphorylated peptides during the antibody binding step. Peptide concentration is shown on the right. Shown are the representative results of three independent dot blot experiments. Source data are provided as a Source Data file.

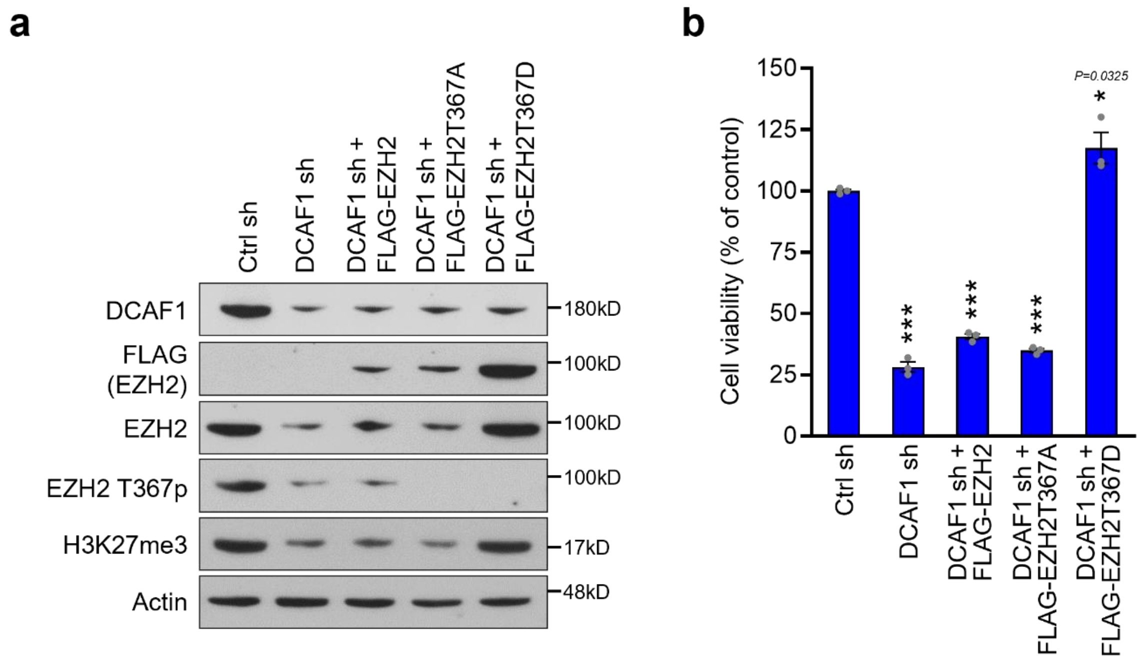

Supplementary Fig. 4 Colon cancer cell growth stimulated by DCAF1-mediated EZH2T367p. a, DCAF1-depleted SW620 cells were infected with lentiviruses expressing 3×FLAG-EZH2 wild type, T367A phospho-blocking mutant, or T367D phospho-mimicking mutant as indicated on the top. Cell lysates were prepared and analyzed by Western blot blotting with the indicated antibodies. Shown are the representative results of three independent immunoblot experiments. **b,** DCAF1-depleted SW620 cells were rescued as in (a), and their viability was measured by WST-1 assays. Data are represented as mean ± SEM (n=3 biologically independent experiments). P values were calculated using one-way ANOVA. *P < 0.05 and ***P < 0.001 versus Ctrl sh. Source data are provided as a Source Data file.

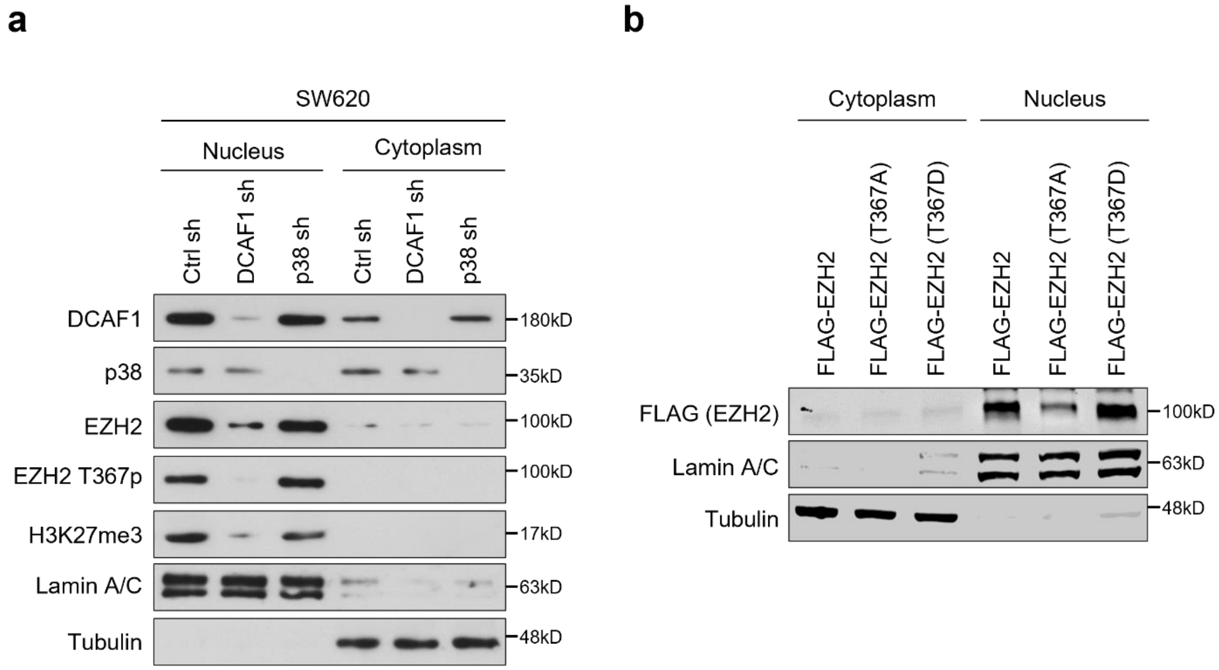

Supplementary Fig. 5 EZH2 phosphorylation and nuclear enrichment regulated by nuclear DCAF1. a, Nuclear and cytoplasmic extracts were fractionated from total lysates of control, DCAF1-depleted, or p38-depleted SW620 cells and analyzed by Western blotting with the indicated antibodies. Shown are the representative results of three independent immunoblot experiments. **b,** Nuclear and cytoplasmic fractionations were prepared from SW620 cells expressing 3×FLAG-EZH2 wild type, T367A phospho-blocking mutant, or T367D phospho-mimicking mutant as indicated on the top, and subjected to Western blotting with anti-FLAG antibody for the detection of ectopic EZH2 proteins. Shown are the representative results of three independent immunoblot experiments. Source data are provided as a Source Data file.

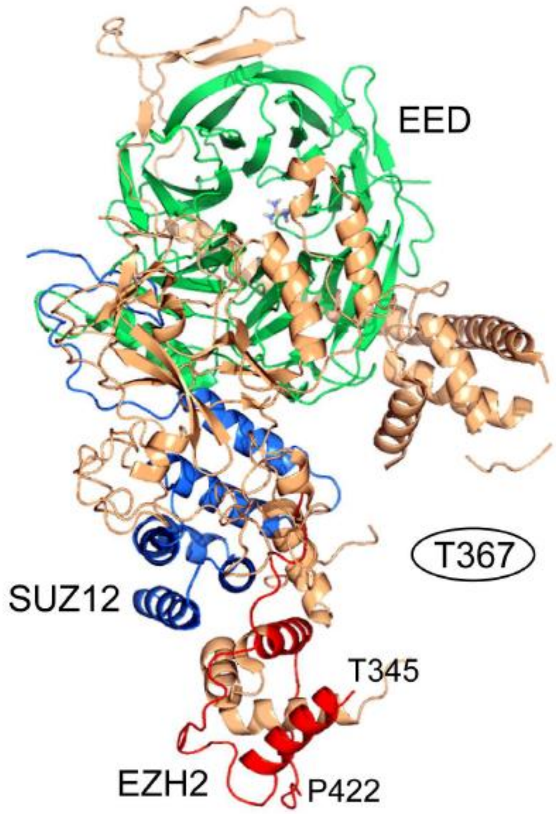

Supplementary Fig. 6 Structure of EZH2 in complex with EED and SUZ12 (PDB ID 5hyn). EZH2 is shown in light red with residues 299-445 depicted in dark red. T367 is not visible in the crystal structure, likely residing in a dynamic loop connecting T345 with P422.

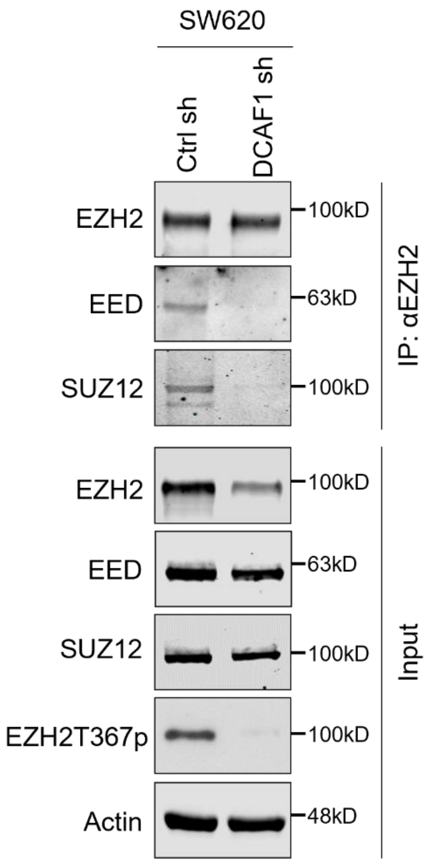

Supplementary Fig. 7 DCAF1-dependent interaction between EZH2 and EED/SUZ12 in colon cancer cells. Whole cell lysates were prepared from control or DCAF1-depleted SW620 cells, immunoprecipitated with EZH2 antibody, and analyzed by Western blotting with EED and SUZ12 antibodies. Shown are the representative results of three independent experiments. Source data are provided as a Source Data file.

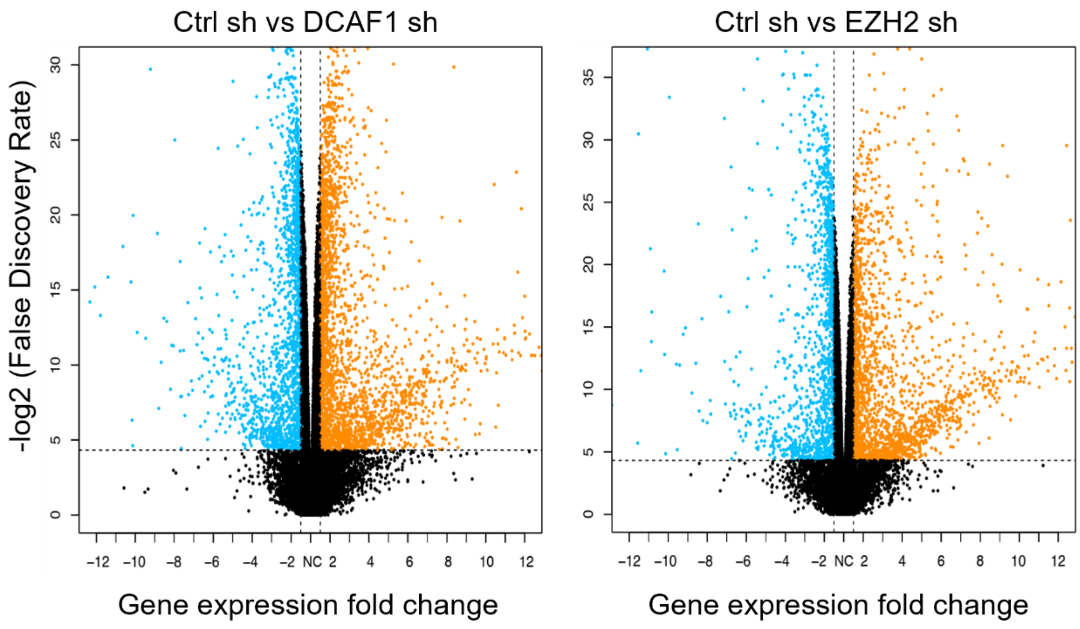

Supplementary Fig. 8 Volcano plot of RNA-seq data. Volcano plots were generated by using the Gene Specific Algorithm from Partek® Flow® software (Partek Inc., MO, USA) and display the global transcriptional changes in DCAF1-depleted or EZH2-depleted SW620 cells compared to mock-depleted control cells. Fold change > 1.5 and false discovery rate cut-off of 0.05 were used to identify genes that have a statistically significant difference in average expression across distinct biological groups. Each dot represents one gene. The X-axis is expression fold change, and the Y-axis is the FDR which is an adjusted *P* values of multiple comparisons.

Sample name	Total reads	Alignment	Unique paired	Genomic coverage	Avg. coverage depth in covered regions	Avg. base quality score	%GC
Control sh1	31,017,805	96.95%	94.46%	5.88%	51.31	36.11	53.15%
Control sh2	31,176,955	97.02%	94.55%	5.90%	51.42	36.07	52.93%
Control sh3	26,944,363	97.63%	95.17%	5.46%	48.43	36.21	51.82%
DCAF1 sh1	25,164,855	97.01%	85.88%	8.48%	34.97	36.05	51.58%
DCAF1 sh2	28,127,814	97.25%	87.21%	11.23%	28.86	36.04	51.46%
DCAF1 sh3	27,856,938	97.16%	91.08%	8.99%	32.90	36.09	51.51%
EZH2 sh1	30,780,217	97.88%	95.50%	7.12%	42.48	36.17	52.08%
EZH2 sh2	33,947,501	97.84%	95.50%	7.31%	45.53	36.12	52.22%
EZH2 sh3	35,410,952	97.80%	95.36%	7.45%	46.70	36.16	51.68%

Supplementary Fig. 9 RNA-seq library quality control metrics.

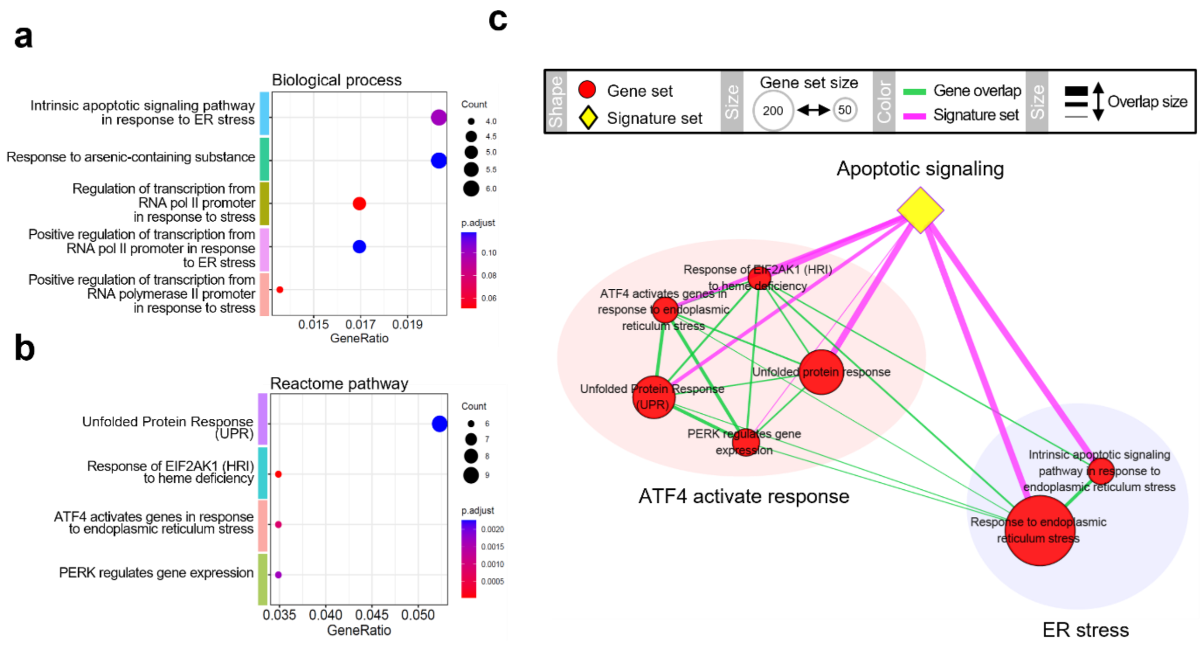

Supplementary Fig. 10 Enrichment map for DCAF1-mediated EZH2 pathway and overlap with known genes related to apoptotic signaling pathway. a, b, Dot plot represents enriched GO term ($p < 0.05$) (a) and Reactome pathway ($p < 0.05$) (b) for the common up-regulated genes shown in Figure 4b. The size of dot is based on the enriched gene count, and the color of the dot indicate the enrichment significance in the pathway. The data were filtered with a Benjamini-Hochberg method by a function implemented in the R package clusterProfiler 4.6.2. (See also Supplementary Table 1.) **c,** The gene list used in (a) and (b) were analyzed to determine the pathway that are statistically enriched in the experiment using g:Profiler (https://biit.cs.ut.ee/gprofiler). The g:Profiler enrichment result was used to generate the enrichment map using a network visualization application EnrichmentMap (ver. 3.3.5) in Cytoscape software (ver. 3.9.1), and the network clusters of gene set (red dots) was annotated by AutoAnnotate application (ver. 1.4.0) in Cytoscape. Overlap between each gene set was indicated with green solid lines. To identify the gene set that have known apoptosis association, the signature analysis using the post-analysis feature was performed in Cytoscape. The yellow diamond represents the gene set of known intrinsic apoptotic signaling from the Molecular Signatures Database (MSigDB). Pink solid lines indicate overlap between the apoptotic signature genes and enriched gene sets and thickness represents significance. The data were scored using one-sided Fisher's Exact Test and overlaps passing the significance thresholds (nominal $p < 0.05$ and FDR < 0.05) were selected, and displayed as pink solid lines. Overlap coefficient was set to 0.5. (See also Supplementary Table 2.)

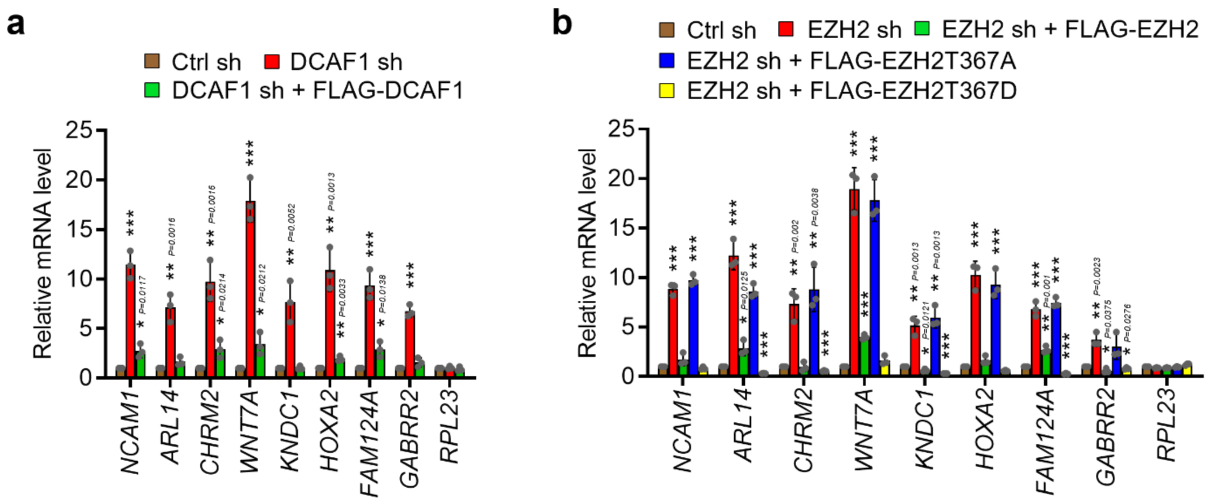

Supplementary Fig. 11 RT-qPCR analysis of EZH2 and DCAF1 targeted genes in Caco2 cells. a, RNA-seq data were validated by RT-qPCR using primers specific for the 8 genes that were upregulated and the 1 gene that was unaffected following DCAF1 knockdown. Also included in this analysis was mRNA extracted from DCAF1-rescued Caco2 cells. The values are expressed as fold changes from the mRNA levels in mock-depleted control cells. Primer sequences are listed in Table S1. Results represent the mean ± SEM (n = 3 biologically independent experiments). *P* values were calculated using two-way ANOVA tests. **P* < 0.05, ***P* < 0.01 and ****P* < 0.001 versus Ctrl sh. **b,** RT-qPCR assays were carried out as in (a), but using EZH2-depleted Caco2 cells. For rescue experiments, EZH2-depleted cells were transfected with shRNA-resistant EZH2 wild type, T367A mutant, or T367D mutant. Results represent the mean ± SEM (n = 3 biologically independent experiments). *P* values were calculated using two-way ANOVA tests. **P* < 0.05, ***P* < 0.01 and ****P* < 0.001 versus Ctrl sh. Source data are provided as a Source Data file.

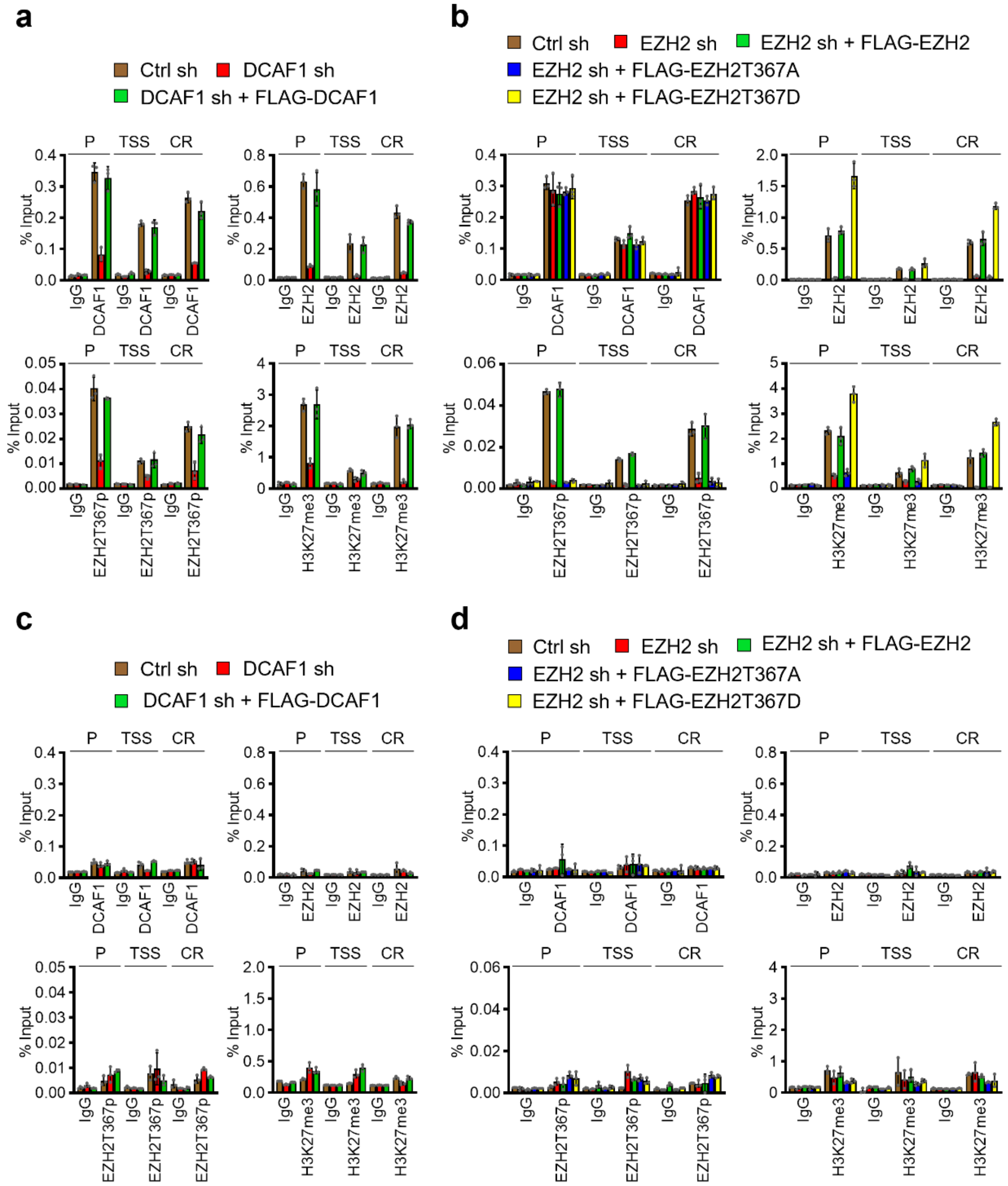

Supplementary Fig. 12 ChIP analysis of HOXA2 and RPL23 loci. a, DCAF1-depleted SW620 cells were transfected with DCAF1, and ChIP assays of HOXA2 gene were performed using DCAF1, EZH2, EZH2T367p, and H3K27me3 antibodies. Data represent the mean ± SEM (n = 3 biologically independent experiments). **b**, ChIP assays were performed as in (a) but using EZH2-depleted SW620 cells transfected with EZH2 wild type and mutants. Data represent the mean ± SEM (n = 3 biologically independent experiments). **c, d,** ChIP assays were performed as in (a) and (b), but on RPL23 gene whose expression is not responsive to DCAF1 and EZH2 knockdown. Data represent the mean ± SEM (n = 3 biologically independent experiments). Source data are provided as a Source Data file.

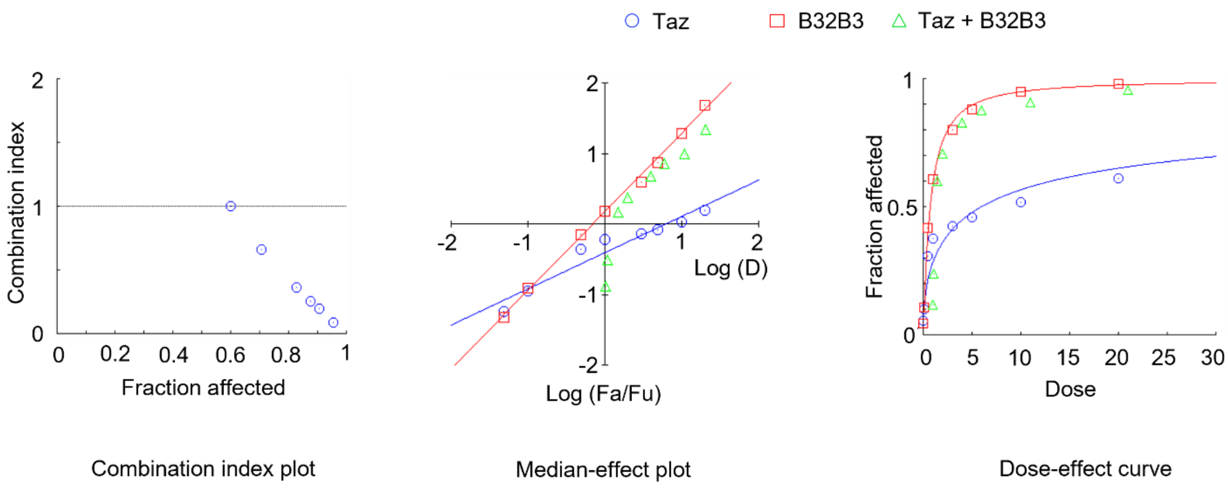

Supplementary Fig. 13 Analysis of inhibitory activity of Taz and B32B3. The fraction affected (Fa) and combination index (CI) were calculated from the cell viability data presented in Fig. 5d using Compusyn software (ComboSyn, Inc., Paramus, NJ, USA). The values obtained from these analyses are shown in Supplementary Table 1, and were used for further calculations of Taz/B32B3 combination index plot, median-effect plot and dose-effect curves. Source data are provided as a Source Data file.

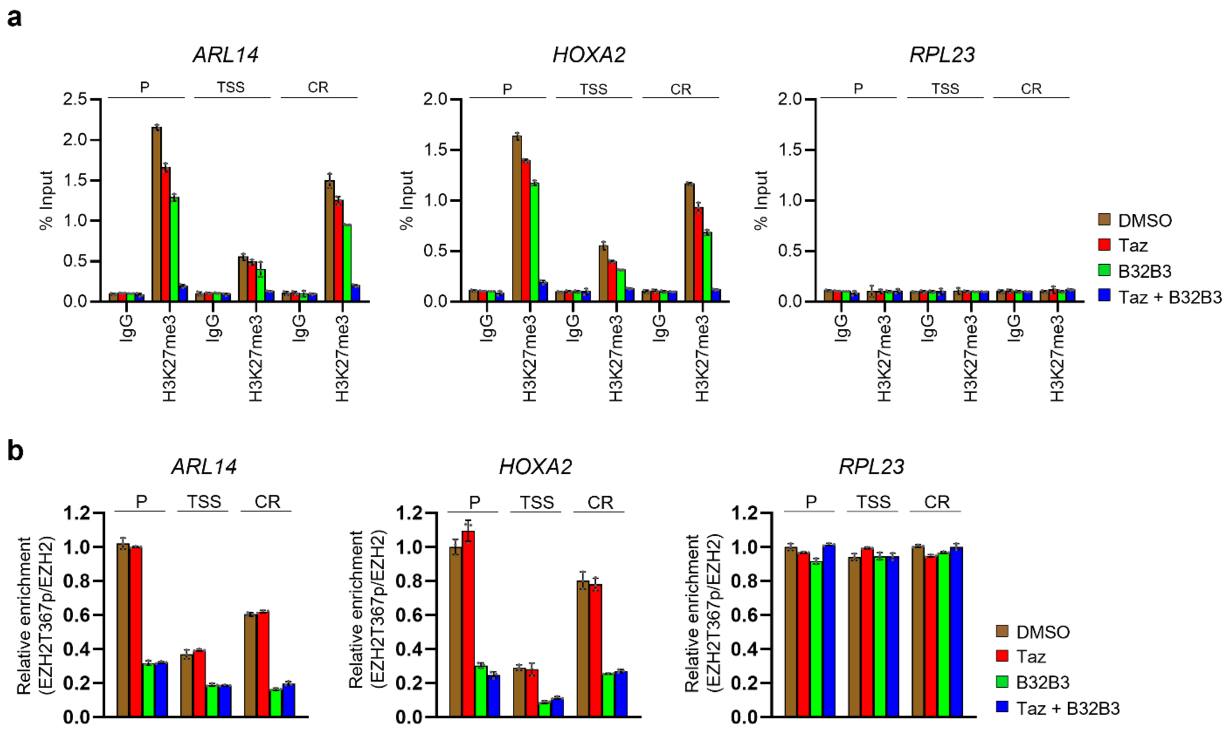

Supplementary Fig. 14 DCAF1 and EZH2 inhibition effects on H3K27me3 and EZH2T367p levels at target genes. a, b, SW620 cells were treated with DCAF1 inhibitor B32B3 (0.9 µM) and/or EZH2 inhibitor Taz (15 µM) for 72 h, and ChIP assays were performed using H3K27me3 (a) and EZH2T367p (b) antibodies. EZH2T367p was normalized with total EZH2 level, and the relative enrichments were represented. Data are represented as mean ± SEM of three independent experiments. Source data are provided as a Source Data file.

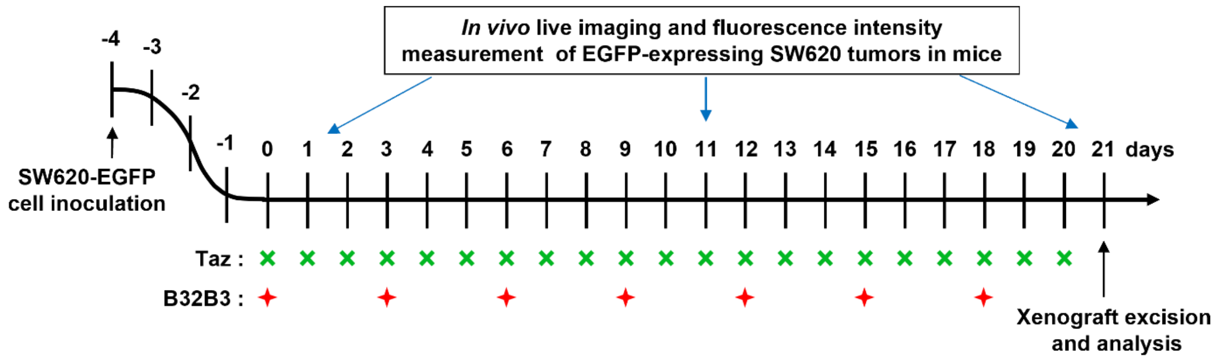

Supplementary Fig. 15 Schematic overview of SW620-EGFP xenograft establishment, inhibitor treatment, and analysis procedures.

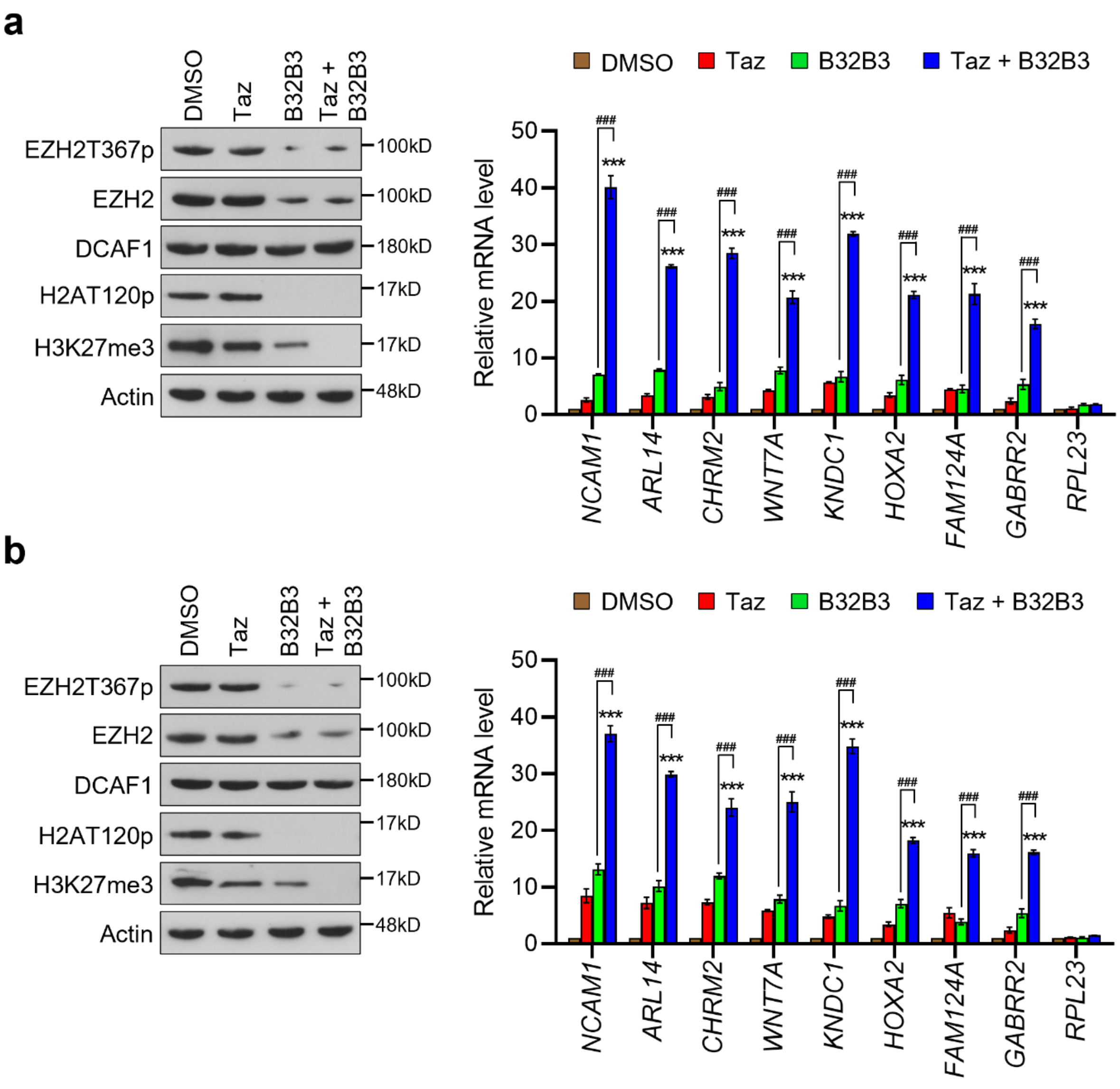

Supplementary Fig. 16 Altered EZH2 protein levels and target gene expression in inhibitor-treated xenografts. After 21-day treatments with Taz and/or B32B3, SW620 xenograft tumors were excised from mice, and two tumor samples (a and b) were subjected to Western blot (left) and RT-qPCR (right) analyses as detailed in our recent studies (Gate et al. 2021 Molecular Oncology 15(10):2801-2817). Data represent the means ± SEM of three independent experiments. *P* values were calculated using one-way ANOVA. ***$P < 0.001$ versus DMSO; ###$P < 0.001$ versus B32B3. Source data are provided as a Source Data file.

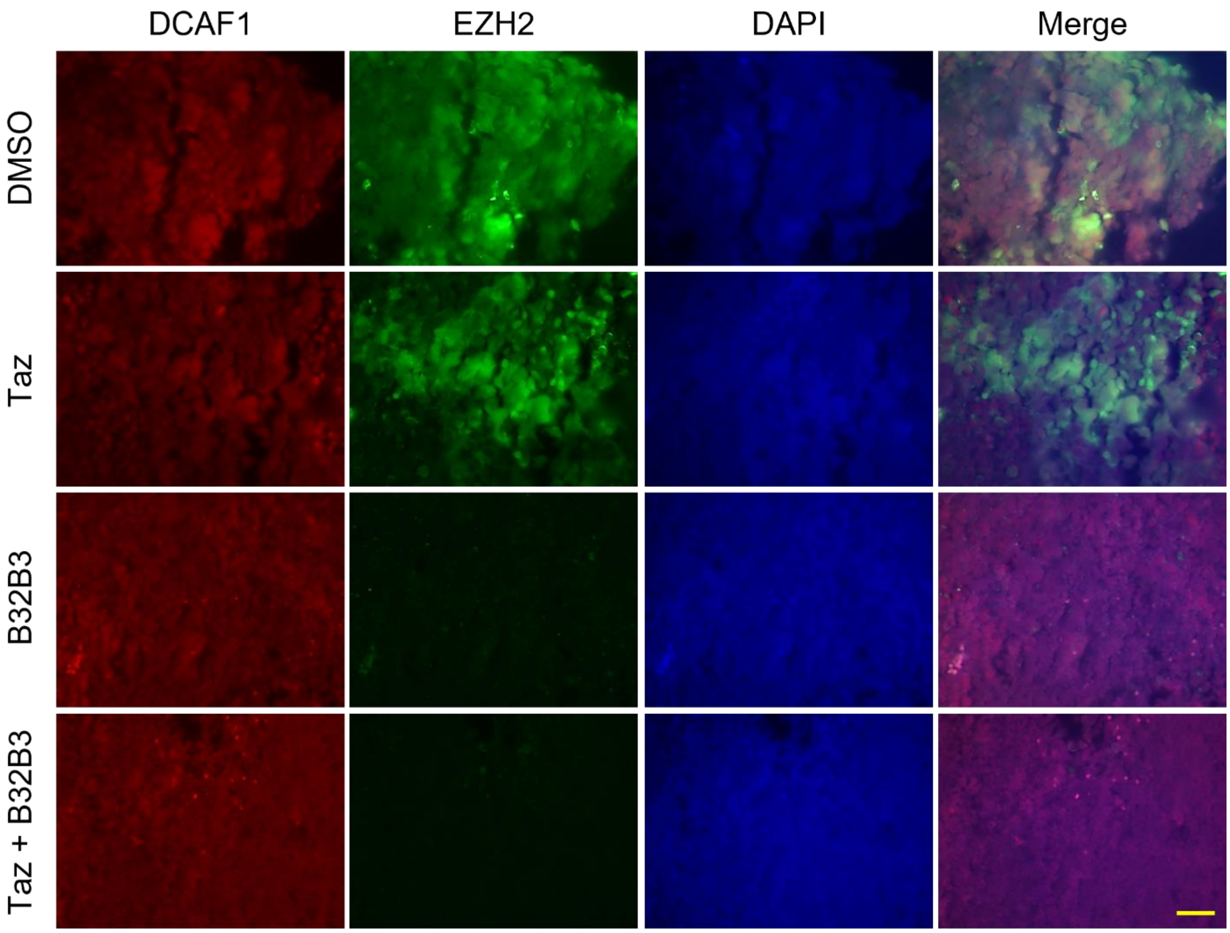

Supplementary Fig. 17 Immunostaining analysis of inhibitor-treated xenograft mice. SW620 tumor xenografts were excised from mice after 21-day treatment with Taz and/or B32B3 and subjected to immunofluorescence staining with EZH2 and DCAF1 antibodies. High-power magnifications are shown for representative immunostaining samples. Shown are the representative results of three independent immunoblot experiments. Scale bar, 10 µm.

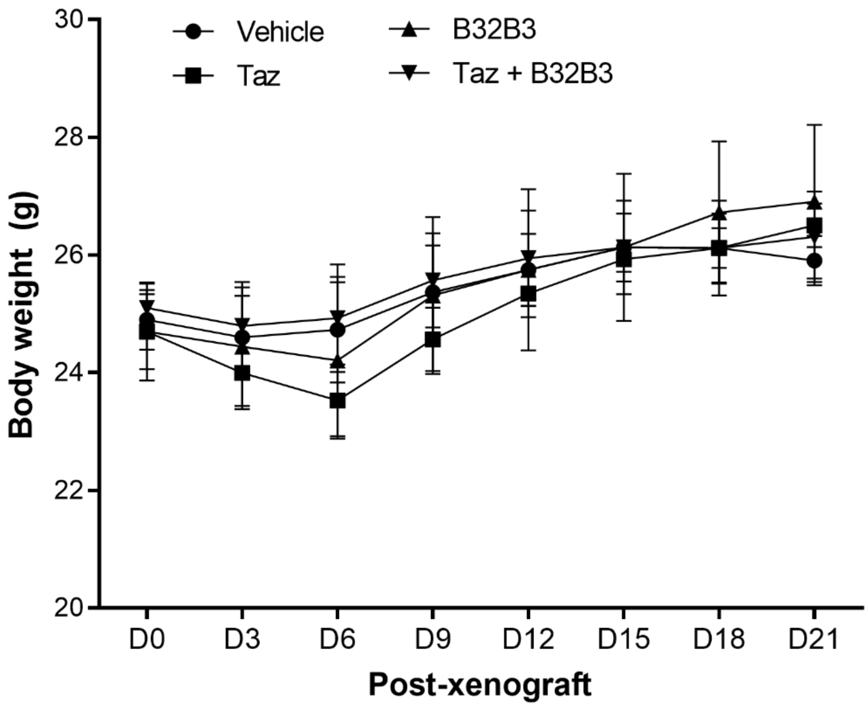

Supplementary Fig. 18 Body weights of inhibitor-treated xenograft mice. Body weights of mice treated with B32B3 and/or Taz were compared with those of vehicle-only treated mice at the indicated time points. Mean body weights (g) ± SEM are shown (n = 8). Source data are provided as a Source Data file.

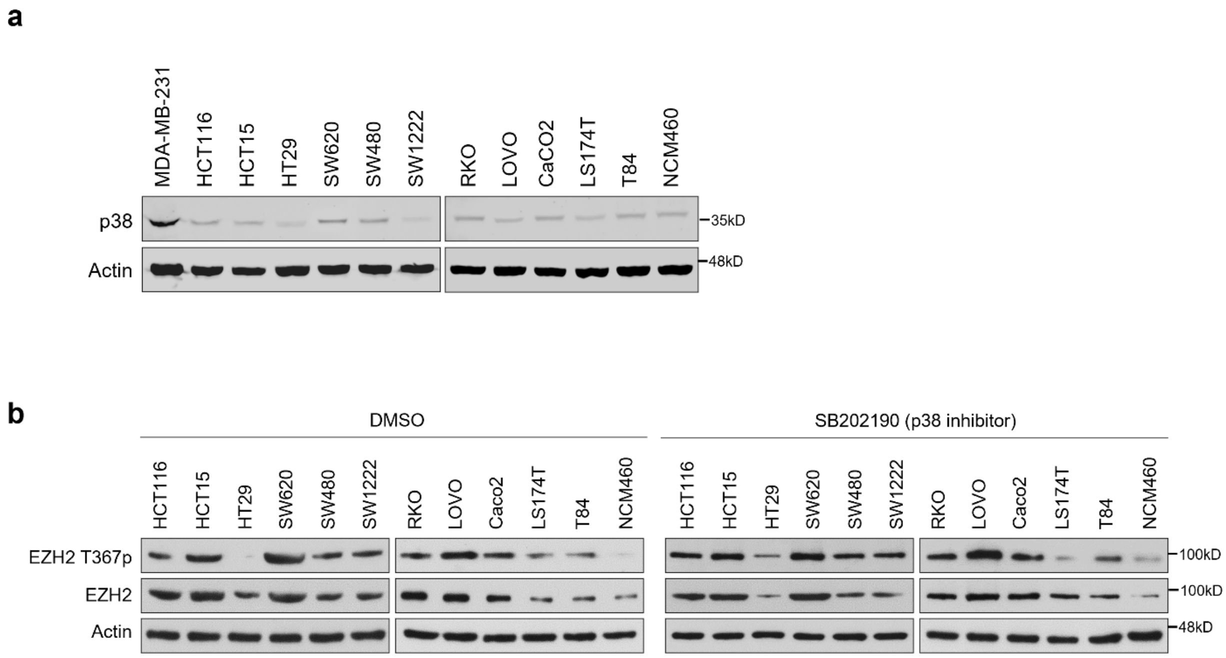

Supplementary Fig. 19 Analysis of p38 protein levels and p38 inhibition effects. a, Cell lysates were prepared from normal colon (NCM460), colon cancer (HCT116, HCT15, HT29, SW620, SW480, SW1222, RKO, LOVO, Caco2, LS174T, and T48) and breast cancer (MDA-MB-231) cell lines and analyzed by Western blotting using p38 antibody. Shown are the representative results of three independent immunoblot experiments. **b,** The cell lines used in (a) were treated with DMSO or the p38 specific inhibitor SB202190 for 24 h. Whole cell lysates were then prepared and subjected to Western blot analysis using EZH2 and EZH2T367p antibodies. Shown are the representative results of three independent immunoblot experiments. Source data are provided as a Source Data file.

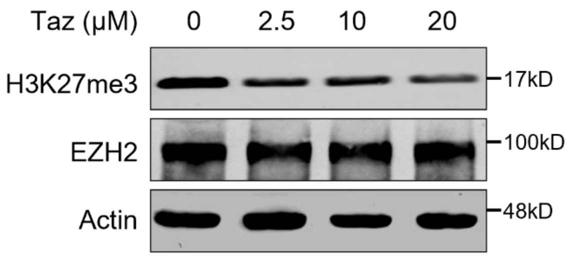

Supplementary Fig. 20 Effects of Taz treatment on H3K27me3 and EZH2 levels. SW620 colon cancer cells were treated with the indicated concentrations of Taz for 72 h. Whole-cell lysates were then prepared and analyzed by Western blotting with H3K27me3 and EZH2 antibodies. Data are representative of three independent experiments. Source data are provided as a Source Data file.

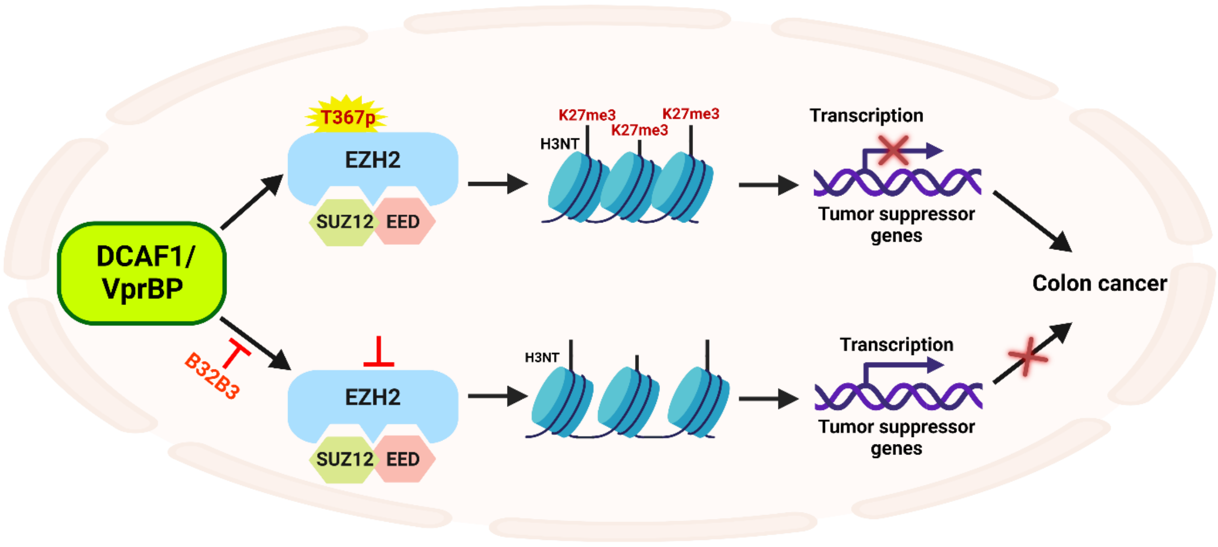

Supplementary Fig. 21 Model for gene silencing by DCAF1-EZH2T367p in colon cancer. Diagram was created with BioRender.com.

Supplementary Table 1. List of enriched GO terms and Reactome pathways for the up-regulated genes from DCAF1 and EZH2 knockdown cells.

GO Biological Process

ID	Description	p-Value	p.adjust	q-Value
GO:1990440	Positive regulation of transcription from RNA polymerase II promoter in response to endoplasmic reticulum stress	0.000018	0.051423	0.049411
GO:0036003	Positive regulation of transcription from RNA polymerase II promoter in response to stress	0.000031	0.051423	0.049411
GO:0043618	Regulation of transcription from RNA polymerase II promoter in response to stress	0.000089	0.099371	0.095482
GO:0046685	Pesponse to arsenic-containing substance	0.000152	0.117824	0.113213
GO:0043620	Pegulation of DNA-templated transcription in response to stress	0.000175	0.117824	0.113213
GO:0071243	Cellular response to arsenic-containing substance	0.000288	0.161307	0.154995
GO:0070059	Intrinsic apoptotic signaling pathway in response to endoplasmic reticulum stress	0.000455	0.218509	0.209958
GO:0001709	Cell fate determination	0.000543	0.228220	0.219289
GO:0034976	Response to endoplasmic reticulum stress	0.000787	0.267682	0.257207
GO:0140013	Meiotic nuclear division	0.000797	0.267682	0.257207

Reactome

ID	Description	p-Value	p.adjust	q-Value
R-HSA-9648895	Response of EIF2AK1 (HRI) to heme deficiency	0.00000006	0.00003435	0.00003323
R-HSA-380994	ATF4 activates genes in response to endoplasmic reticulum stress	0.00000320	0.00086775	0.00083940
R-HSA-381042	PERK regulates gene expression	0.00000916	0.00165874	0.00160456
R-HSA-381119	Unfolded Protein Response (UPR)	0.00001668	0.00226477	0.00219079

Supplementary Table 2. Result of enrichment analysis for the up-regulated genes.

ID	Description	p-Value	FDR
R-HAS-381042	PERK regulates gene expression	0.0325498798	0.0325498798
GO:0070059	Intrinsic apoptotic signaling pathway in response to endoplasmic reticulum stress	0.0253239595	0.0253239595
R-HSA-381119.2	Unfolded Protein Response (UPR)	0.0253239595	0.0253239595
R-HSA-381119	Unfolded protein response	0.0333057013	0.0333057013
R-HAS-380994	ATF4 activates genes in response to endoplasmic reticulum stress	0.0242453056	0.0242453056
R-HAS-9648895	Response of EIF2AK1 (HRI) to heme deficiency	0.0070350246	0.0070350246
GO:0034976	Response to endoplasmic reticulum stress	0.0458366644	0.0458366644

Supplementary Table 3. The fraction affected (Fa) and combination index (CI) values at the indicated doses of EPZ6438 and B32B3.

Graph points	EPZ6438 (μM)	B32B3 (μM)	Fa	CI
1	0.05	0.9	0.118453	8.88907
2	0.10	0.9	0.238464	4.12910
3	0.50	0.9	0.601425	1.00706
4	1.00	0.9	0.708454	0.66232
5	3.00	0.9	0.828454	0.36532
6	5.00	0.9	0.878454	0.25604
7	10.00	0.9	0.908343	0.19885
8	20.00	0.9	0.957343	0.09415

Supplementary Table 4. List of the primers used in RT-qPCR.

**Gene Name**	**Forward (5'-3')**	**Reverse primer (5'-3')**
DCAF1	TGAGGGTGGCATTCTTGTCC	TCCCAATATAGCTGCGCTGG
EZH2	GTGGAGAGATTATTTCTCAAGATG	CCGACATACTTCAGGGCATCAGCC
NCAM1	AGAAGCAAGAGACTCTGGATGG	CCCTGTAGCTTTGGGGCATA
ARL14	TGACCGGAACTGGTATGTGC	ACGCCAAAGTGTCTCCTCTT
CHRM2	CCCTGGGCCATTCCAAAGAT	TCTTCACAATCTTGCGGGCT
WNT7A	CATGGTCTACCTCCGGATCG	AAACTGACACTCGTCCAGGC
KNDC1	GATTGTGACCAGCCACACCT	GAATTCTCCAGGCAGGGGAC
HOXA2	GCTTGTCAACAATGGGCCAG	AAAGCTGCAGGCAGGAATCT
FAM124A	CTCCACGAGCAGTGAGCTTT	GCCAGGACGTTGTCGATGG
GABRR2	GGAAATGCCCAAGCCAAGTC	GAAGTCCATGTCCACCTCGG
RPL23	GTGAAGGGGATCAAGGGACG	GTCGAATGACCACTGCTGGA
β-actin	GTGGGGCGCCCCAGGCACCA	CTCCTTAATGTCACGCACGATTTC

Supplementary Table 5. List of the primers used in ChIP-qPCR.

Gene Name	Forward (5'-3')	Reverse primer (5'-3')
ARL14 (P)	TTTGGCTTCTTGTTACCTTCTGC	AGTGCTGATGCAGAGGAGATAG
ARL14 (TSS)	AGATCCCTCAGGATTGGCTG	GAAAGCAAATTCTTCATGCTGT
ARL14 (CR)	GAGCTCTGACTGCTGAGGAC	GCACATACCAGTTCCGGTCA
HOXA2 (P)	GAAATACTCACCGCACCCGA	TGTGTCCAAATCCCCGTAGC
HOXA2 (TSS)	GGGCAGAAGCAATCATGTGAC	ATTACAGCCGTATGGGGACC
HOXA2 (CR)	TTGCTCCCGGATGAAGAGGT	GCTATTGTGCTGCCTTTCCTG
RPL23 (P)	ACGGGTCAATAAATAGAGCAATACT	ATACAGTTGAGAAGTGCTGCAGAT
RPL23 (TSS)	TAATAAGGCAGCGCCCAGAG	CTTCGACATCTTGAACGCCG
RPL23 (CR)	TACTGATGGAACGGCCTGATG	CCTTGATCCCCTTCACGGAG
