# Supplementary material for: Phosphorylation and stabilization of EZH2 by DCAF1/VprBP trigger aberrant gene silencing in colon cancer
Source: Nat Commun. 2023 Apr 17;14:2140. doi: 10.1038/s41467-023-37883-1 (PMC10110550; doi:10.1038/s41467-023-37883-1)
Supplement: Supplementary file 2 — Reporting Summary [file 41467_2023_37883_MOESM2_ESM.pdf]

nature portfolio

Corresponding author(s): NCOMMS-22-00400B

Last updated by author(s): Mar 28, 2023

Reporting Summary

Nature Portfolio wishes to improve the reproducibility of the work that we publish. This form provides structure for consistency and transparency in reporting. For further information on Nature Portfolio policies, see our Editorial Policies and the Editorial Policy Checklist.

Statistics

For all statistical analyses, confirm that the following items are present in the figure legend, table legend, main text, or Methods section.

n/a	Confirmed	
☐	☒	The exact sample size (*n*) for each experimental group/condition, given as a discrete number and unit of measurement
☐	☒	A statement on whether measurements were taken from distinct samples or whether the same sample was measured repeatedly
☐	☒	The statistical test(s) used AND whether they are one- or two-sided<br>*Only common tests should be described solely by name; describe more complex techniques in the Methods section.*
☒	☐	A description of all covariates tested
☒	☐	A description of any assumptions or corrections, such as tests of normality and adjustment for multiple comparisons
☐	☒	A full description of the statistical parameters including central tendency (e.g. means) or other basic estimates (e.g. regression coefficient) AND variation (e.g. standard deviation) or associated estimates of uncertainty (e.g. confidence intervals)
☐	☒	For null hypothesis testing, the test statistic (e.g. *F*, *t*, *r*) with confidence intervals, effect sizes, degrees of freedom and *P* value noted<br>*Give P values as exact values whenever suitable.*
☒	☐	For Bayesian analysis, information on the choice of priors and Markov chain Monte Carlo settings
☒	☐	For hierarchical and complex designs, identification of the appropriate level for tests and full reporting of outcomes
☐	☒	Estimates of effect sizes (e.g. Cohen's *d*, Pearson's *r*), indicating how they were calculated

Our web collection on statistics for biologists contains articles on many of the points above.

Software and code

Policy information about availability of computer code

Data collection	Cell viability data were collected using CLARIOstar 3.41 software. Western blot data were collected using Image Studio 5.x. RT-qPCR data were collected by Agilent Aria 1.71 software.
Data analysis	All statistical data analyses were performed using Graphpad Prism 9 software.<br>For RNA-seq analysis, RNA-seq reads were mapped to hg38 GENCODE version 29 (PMID: 30357393) using STAR 2.6.1d and differentially expressed genes were selected using the Gene Specific Algorithm from Partek® Flow® software.<br>Gene ontology (GO) and Reactome pathways were analyzed using clusterProfiler 4.6.2 and ReactomePA 1.42 packages in the R software 4.2.2.<br>Functional enrichments were analyzed using the Cytoscape 3.9.1 plug-in EnrichmentMap 3.3.5 and AutoAnnotate 1.4.0.

For manuscripts utilizing custom algorithms or software that are central to the research but not yet described in published literature, software must be made available to editors and reviewers. We strongly encourage code deposition in a community repository (e.g. GitHub). See the Nature Portfolio guidelines for submitting code & software for further information.

Data

Policy information about availability of data

All manuscripts must include a data availability statement. This statement should provide the following information, where applicable:

- Accession codes, unique identifiers, or web links for publicly available datasets
- A description of any restrictions on data availability
- For clinical datasets or third party data, please ensure that the statement adheres to our policy

The data that support the findings of this study are available in NCBI's Gene Expression Omnibus and are accessible through the following links: https://www.ncbi.nlm.nih.gov/geo/query/acc.cgi?acc=GSE180282, GEO Series accession number [GSE180282] for RNA-seq study performed in DCAF1 sh SW620 cells, and https://www.ncbi.nlm.nih.gov/geo/query/acc.cgi?acc=GSE192489, GEO Series accession number [GSE192489] for RNA-seq study performed in EZH2 sh SW620 cells. The human reference genome hg38 GENCODE version 29 (PMID: 30357393) used in this study is available to download from https://www.gencodegenes.org/human/release_29.html.

Human research participants

Policy information about studies involving human research participants and Sex and Gender in Research.

Reporting on sex and gender	N/A
Population characteristics	N/A
Recruitment	N/A
Ethics oversight	N/A

Note that full information on the approval of the study protocol must also be provided in the manuscript.

Field-specific reporting

Please select the one below that is the best fit for your research. If you are not sure, read the appropriate sections before making your selection.

☒ Life sciences ☐ Behavioural & social sciences ☐ Ecological, evolutionary & environmental sciences

For a reference copy of the document with all sections, see nature.com/documents/nr-reporting-summary-flat.pdf

Life sciences study design

All studies must disclose on these points even when the disclosure is negative.

Sample size	For in vitro study, no samples size calculation was performed. All the experiments were repeated at least three independent experiments that are commonly used to estimate the effect of the treatment in this fields. For animal study, sample size was determined to be adequate based on the magnitude and consistency of measurable differences between groups.
Data exclusions	Data were not excluded from analysis.
Replication	All in vitro experiments were repeated at least three independent times with triplicates. All replication attempts were successful with reproducible results. For animal study, 8 mice were used for each group.
Randomization	For in vitro study using cell lines and PDOs, samples were analyzed equally with no sub-sampling and thus there was no requirement for randomization. In in vivo experiments, the study included 32 mice were randomly allocated to four intervention groups.
Blinding	For animal study, the investigators were blinded for the animal allocation to the experimental groups. All data collection and analyses were performed in a blind manner. For in vitro study, blinding was not applicable because the investigator in charge of an experiment was responsible for cell culture, treatment, sample collection, and data analysis.

Reporting for specific materials, systems and methods

We require information from authors about some types of materials, experimental systems and methods used in many studies. Here, indicate whether each material, system or method listed is relevant to your study. If you are not sure if a list item applies to your research, read the appropriate section before selecting a response.

Materials & experimental systems

n/a	Involved in the study
☐	☒ Antibodies
☐	☒ Eukaryotic cell lines
☒	☐ Palaeontology and archaeology
☐	☒ Animals and other organisms
☒	☐ Clinical data
☒	☐ Dual use research of concern

Methods

n/a	Involved in the study
☒	☐ ChIP-seq
☒	☐ Flow cytometry
☒	☐ MRI-based neuroimaging

Antibodies

Antibodies used

Anti-Actin (mouse monoclonal, clone 2D4H5); Proteintech; Cat #66009-1-Ig; Lot# 10004156; 1:1000 for WB
Anti-HA tag (rabbit polyclonal); Proteintech; Cat# 51064-2-AP; Lot# 00088683
Anti-His tag (mouse monoclonal, clone 1B7G5); Proteintech; Cat# 66005-1-Ig; Lot# 10004365
Anti-VprBP (rabbit polyclonal); Proteintech; Cat# 11612-1-AP; Lot# 00055511
Anti-VprBP (mouse monoclonal, clone 1A7A8); Proteintech; Cat# 66392-1-Ig; Lot# 10005116
Anti-EZH2 (rabbit polyclonal); Proteintech; Cat# 21800-1-AP; Lot# 00048858
Anti-EED (rabbit polyclonal); Proteintech; Cat# 16818-1-AP; Lot# 00047636
Anti-SUZ12 (rabbit polyclonal); Proteintech; Cat# 20366-1-AP; Lot# 00020297
Anti-H2AT120p (rabbit polyclonal); Active Motif; Cat# 39391; Lot# 30508001
Anti-H3K4me3 (rabbit polyclonal); Active Motif; Cat# 39915; Lot# 14013006
Anti-H3K9me3 (rabbit polyclonal); Abcam; Cat# ab8898; Lot# 733946
Anti-H3K36me3 (rabbit polyclonal); Abcam; Cat# ab9050; Lot# 134026
Anti-H3Y41p (rabbit polyclonal); Abcam; Cat# ab26310 discontinued; Not Available
Anti-H3K79me3 (rabbit polyclonal); Abcam; Cat# ab2621; Lot# GR65540-1
Anti-H3S10p (mouse monoclonal, clone RR002); Millipore Sigma; Cat# 05-598; Lot# 22980
Anti-H2BS14p (rabbit polyclonal); Millipore Sigma; Cat# 07-191; Lot# 20373
Anti-FLAG® M2 (mouse monoclonal, clone M2); Millipore Sigma; Cat# F3165; Lot# SLBT6752
Anti-H3ac (rabbit polyclonal); Millipore Sigma; Cat# 06-599; Lot# JBC1893961
Anti-H4ac (rabbit polyclonal); Millipore Sigma; Cat# 06-598; Lot# DAM1748593
Anti-H3K27me3 (rabbit polyclonal); Millipore Sigma; Cat# 07-449; Lot# 2475696
Anti-Rabbit IgG (goat polyclonal); Thermo Fisher Scientific; Cat# 31460; Lot# VJ313046
Anti-Mouse IgG (goat polyclonal); Thermo Fisher Scientific; Cat# G21040; Lot# 1556183
Anti-Rabbit IgG, Fluorescein (FITC)-AffiniPure Fab Fragment (goat polyclonal); Jackson ImmunoResearch Labs; Cat# 115-097-003; Lot# 154785
Anti-Mouse IgG, Alexa Fluor 594 (goat polyclonal); Thermo Fisher Scientific; Cat# A-11032; Lot# 1887003

Validation

Anti-Actin (mouse monoclonal); RRID: AB_2687938, Validated in human, pig, rabbit, rat, mouse, chicken by WB, IP, IHC, IF, and FC on manufacture's website (https://www.ptglab.com/products/Pan-Actin-Antibody-66009-1-Ig.htm)
Anti-HA tag (rabbit polyclonal); RRID: AB_11042321, Validated in human by WB, IP, and IF on manufacture's website (https://www.ptglab.com/products/HA-tag-Antibody-51064-2-AP.htm)
Anti-His tag (mouse monoclonal); RRID: AB_11232599, Validated in human by WB, IP, and IF on manufacture's website (https://www.ptglab.com/products/His-Tag-Antibody-66005-1-Ig.htm)
Anti-VprBP (rabbit polyclonal); RRID: AB_2216933, Validated in human by WB, IP, IHC, IF, and FC on manufacture's website (https://www.ptglab.com/products/VPRBP-Antibody-11612-1-AP.htm)
Anti-VprBP (mouse monoclonal); RRID: AB_2881768 ,Validated in human by WB, HIC, and IF on manufacture's website (https://www.ptglab.com/products/VPRBP-Antibody-66392-1-Ig.htm)
Anti-EZH2 (rabbit polyclonal); RRID: AB_10858790, Validated in human by IP, IF, and FC on manufacture's website (https://www.ptglab.com/products/EZH2-Antibody-21800-1-AP.htm)
Anti-EED (rabbit polyclonal); RRID: AB_2262065, Validated in human by WB, IP, IF, and IHC on manufacture's website (https://www.ptglab.com/products/EED-Antibody-16818-1-AP.htm)
Anti-SUZ12 (rabbit polyclonal); RRID: AB_10694152, Validated in human by WB, and DB on manufacture's website (https://www.ptglab.com/products/SUZ12-Antibody-20366-1-AP.htm)
Anti-H2AT120p (rabbit polyclonal); RRID: AB_2744670, Validated in human by WB, IP, and IHC on manufacture's website (https://www.activemotif.com/catalog/details/39391/histone-h2a-phospho-thr120-antibody-pab)
Anti-H3K4me3 (rabbit polyclonal); RRID: AB_2687512, Validated in human and budding yeast by ChIP, ChIP-seq, WB, IF, DB, and ICC on manufacture's website (https://www.activemotif.com/catalog/details/39915/histone-h3-trimethyl-lys4-antibody-pab-1)
Anti-H3K9me3 (rabbit polyclonal); RRID: AB_306848, Validated in human, mouse, and cow by ChIP, WB, IHC, IF, and ICC on manufacture's website (https://www.abcam.com/products/primary-antibodies/histone-h3-tri-methyl-k9-antibody-chip-grade-ab8898.html)
Anti-H3K36me3 (rabbit polyclonal); RRID: AB_306966, Validated in human and cow by ChIP, WB, IF, and ICC on manufacture's website (https://www.abcam.com/products/primary-antibodies/histone-h3-tri-methyl-k36-antibody-chip-grade-ab9050.html)
Anti-H3Y41p (rabbit polyclonal); RRID: AB_1951940, Validated in human by WB and ELISA (discontinued, download archived datasheet at https://www.abcam.com/histone-h3-phospho-y41-antibody-ab26310.html)
Anti-H3K79me3 (rabbit polyclonal); RRID: AB_303215, Validated in human and cow by ChIP and WB on manufacture's website (https://www.abcam.com/products/primary-antibodies/histone-h3-tri-methyl-k79-antibody-chip-grade-ab2621.html)
Anti-H3S10p (mouse monoclonal); RRID: AB_309832, Validated in human by WB, IHC and ICC on manufacture's website (https://www.sigmaaldrich.com/US/en/product/mm/05598)
Anti-H2BS14p (rabbit polyclonal); RRID: AB_310420, Validated in human by WB, ICC, IHC, and ELISA on manufacture's website (https://www.sigmaaldrich.com/US/en/product/mm/07191)

	Anti-FLAG® M2 (mouse monoclonal); RRID: AB_259529, Validated by WB on manufacture's website (https://www.sigmaaldrich.com/US/en/product/sigma/f3165)<br>Anti-H3ac (rabbit polyclonal); RRID: AB_2115283, Validated in human, mouse, and rabbit by WB, ChIP, ChIP-seq, and ICC on manufacture's website (https://www.emdmillipore.com/US/en/product/Anti-acetyl-Histone-H3-Antibody,MM_NF-06-599?ReferrerURL=https%3A%2F%2Fwww.google.com%2F)<br>Anti-H4ac (rabbit polyclonal); RRID: AB_2295074, Validated in human by WB, ChIP, IP, and ICC on manufacture's website (https://www.emdmillipore.com/US/en/product/Anti-acetyl-Histone-H4-Antibody,MM_NF-06-598?ReferrerURL=https%3A%2F%2Fwww.google.com%2F)<br>Anti-H3K27me3 (rabbit polyclonal); RRID: AB_310624, Validated in human and mouse by WB, IP, IHC, and ICC on manufacture's website (https://www.emdmillipore.com/US/en/product/Anti-trimethyl-Histone-H3-Lys27-Antibody,MM_NF-07-449?ReferrerURL=https%3A%2F%2Fwww.google.com%2F)<br>Anti-Rabbit IgG (goat polyclonal); RRID_AB228341, Validated in rabbit IgG by WB, IP, IHC, and ELISA on manufacture's website (https://www.thermofisher.com/antibody/product/Goat-anti-Rabbit-IgG-H-L-Secondary-Antibody-Polyclonal/31460)<br>Anti-Mouse IgG (goat polyclonal); RRID:AB_2536527, Validated in mouse IgG by WB, IHC, and ELISA (https://www.thermofisher.com/antibody/product/Goat-anti-Mouse-IgG-H-L-Cross-Adsorbed-Secondary-Antibody-Polyclonal/G-21040)<br>Anti-Rabbit IgG, Fluorescein (FITC)-AffiniPure Fab Fragment (goat polyclonal); RRID:AB_2337984, Validated in previous publications (https://doi.org/10.1038/s41467-019-11820-7, https://doi.org/10.1038/s41596-021-00635-w)<br>Anti-Mouse IgG, Alexa Fluor 594 (goat polyclonal); RRID:AB_2534091, Validated in mouse IgG by WB, ICC, IF and FACS on manufacture's website (https://www.thermofisher.com/antibody/product/Goat-anti-Mouse-IgG-H-L-Highly-Cross-Adsorbed-Secondary-Antibody-Polyclonal/A-11032)

Eukaryotic cell lines

Policy information about cell lines and Sex and Gender in Research

Cell line source(s)	HCT116, HCT15, HT29, RKO, LOVO; Source: Dr. Gangning Liang, University of Southern California.<br>SW1222, LS174T; Source: Dr. Steven M. Larson, Memorial Sloan Kettering Cancer Center .<br>T84, SW480; Source: Dr. Adam E. Snook, Thomas Jefferson University.<br>293T, SW620, Caco2; Source: ATCC<br>NCM460; Source: Dr. Charalabos Pothoulakis, UCLA Center for Inflammatory Bowel Diseases.<br>All originally purchased from ATCC (American Type Culture Collection, Manassas, VA, USA) except SW1222 that obtained from the Ludwig Center for Cancer Immunotherapy (New York, NY, USA).
Authentication	Authentication was not required from our end, as all the cell lines were obtained from ATCC which comprehensively performs authentication and quality control tests on all distribution lots of cell lines.
Mycoplasma contamination	Since all the cell lines were from ATCC, and since ATCC tested them for a mycoplasma contamination, it was not necessary to check for mycoplasma contamination in our cell culture experiments.
Commonly misidentified lines<br>(See ICLAC register)	No commonly misidentified cell lines were used.

Animals and other research organisms

Policy information about studies involving animals; ARRIVE guidelines recommended for reporting animal research, and Sex and Gender in Research

Laboratory animals	8-week-old athymic nude mice [(Crl:NU(NCr)-Foxn1nu]
Wild animals	No wild animals were used in this study.
Reporting on sex	Used both sexes for xenograft model in this study.
Field-collected samples	No field-collected samples were used in this study.
Ethics oversight	All mouse experiments were performed according to protocols approved by the Institutional Animal Care and Use Committee.

Note that full information on the approval of the study protocol must also be provided in the manuscript.
