# Supplementary material for: Phosphorylation and stabilization of EZH2 by DCAF1/VprBP trigger aberrant gene silencing in colon cancer
Source: Nat Commun. 2023 Apr 17;14:2140. doi: 10.1038/s41467-023-37883-1 (PMC10110550; doi:10.1038/s41467-023-37883-1)

Figure 1a

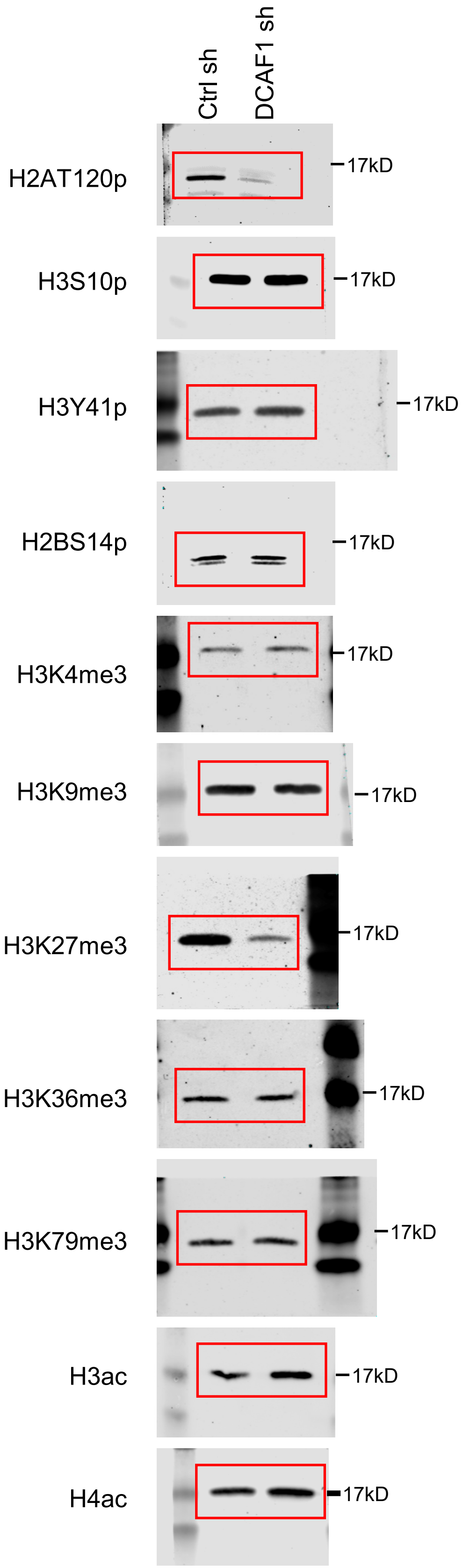

Figure 1a

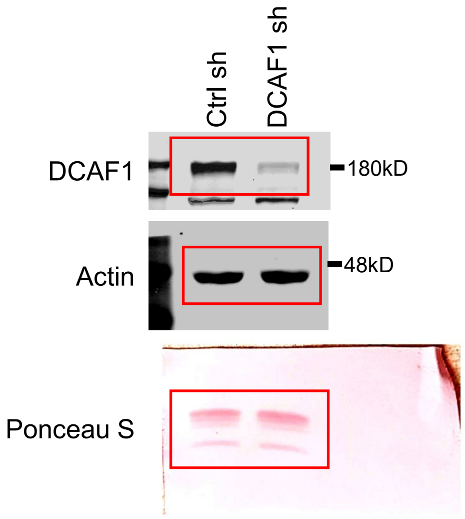

Figure 1B

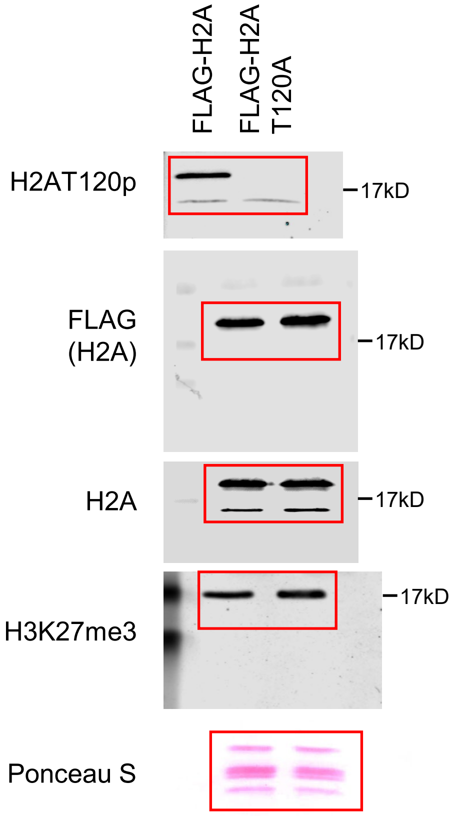

Figure 1c

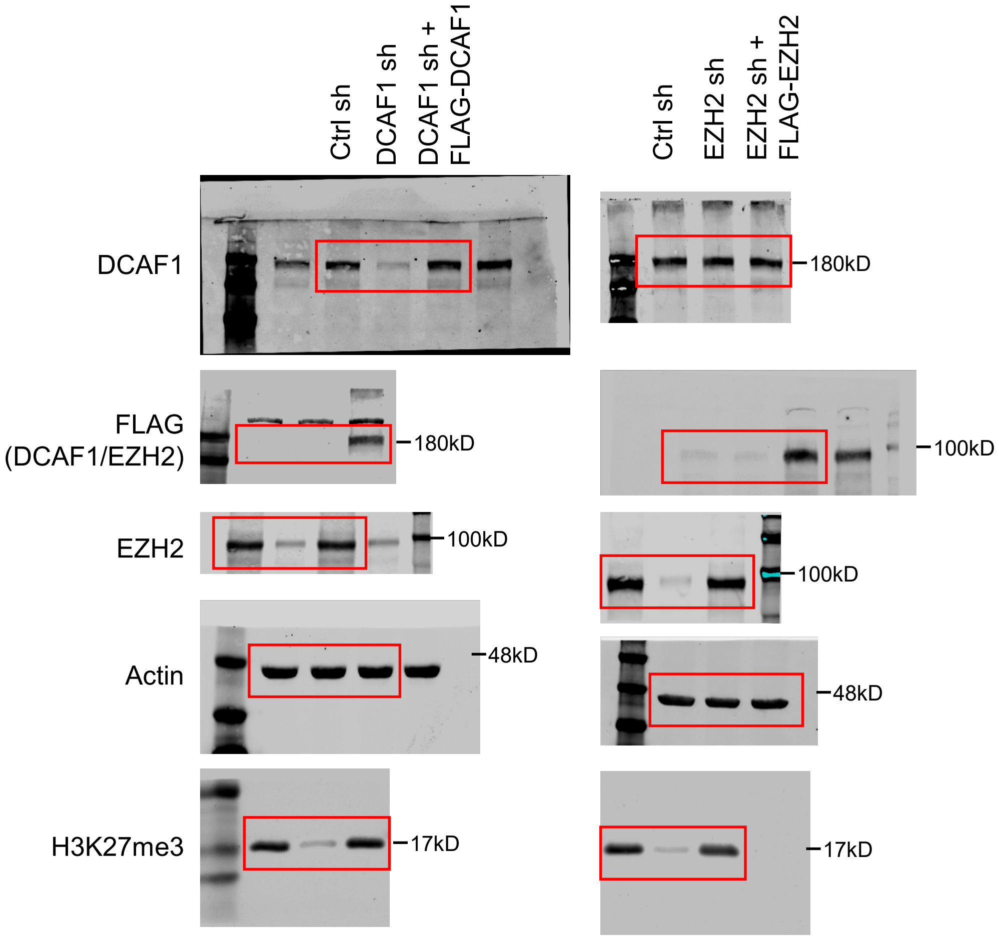

Figure 1e

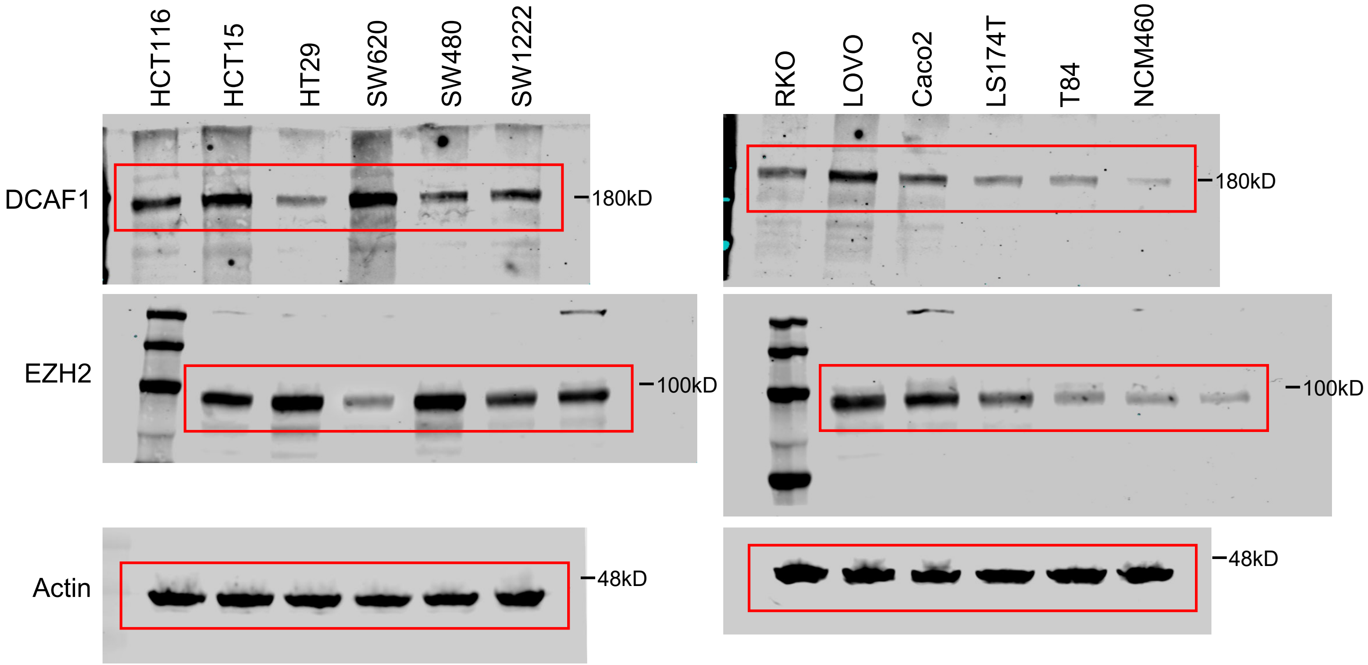

Figure 1f

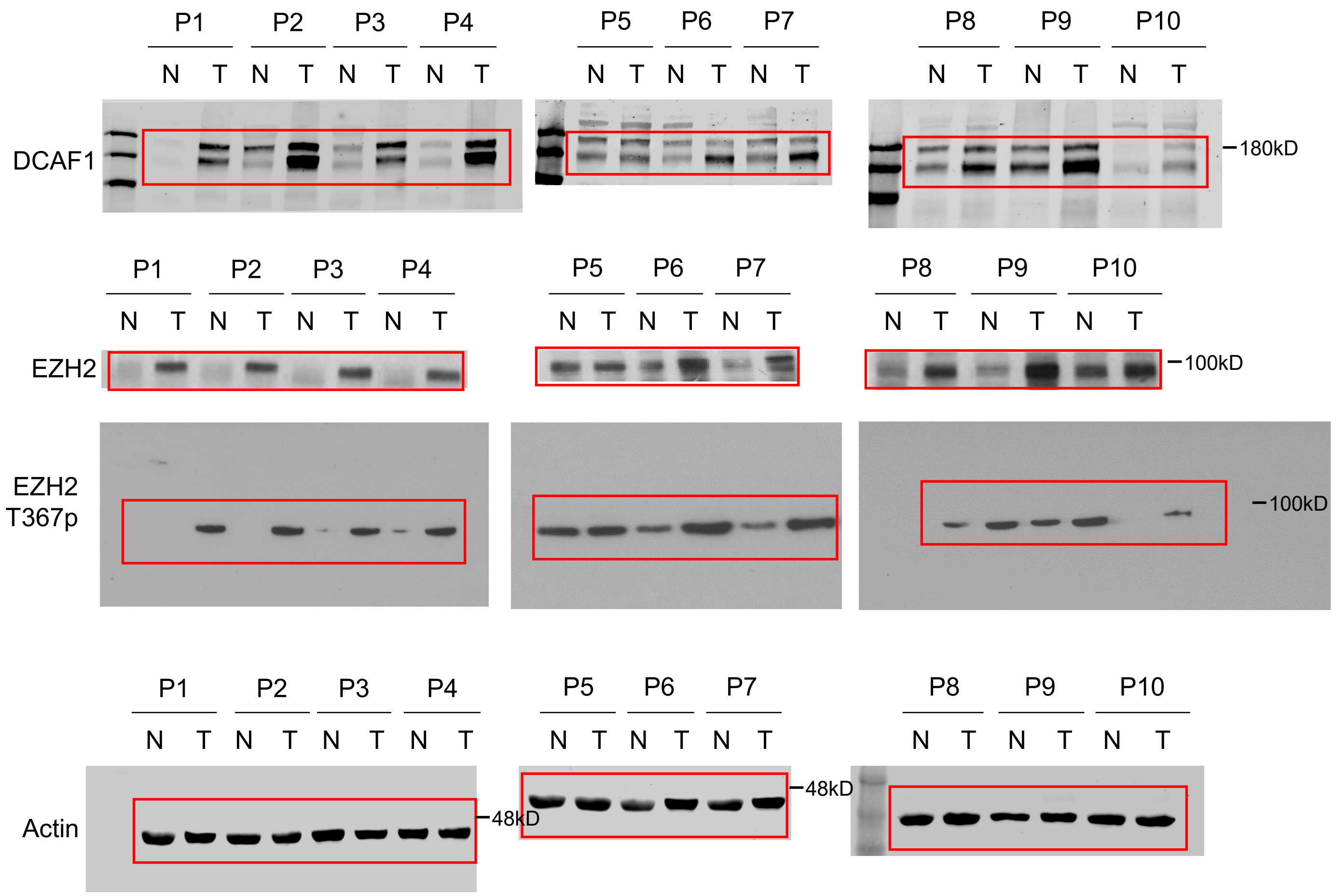

Figure 2a

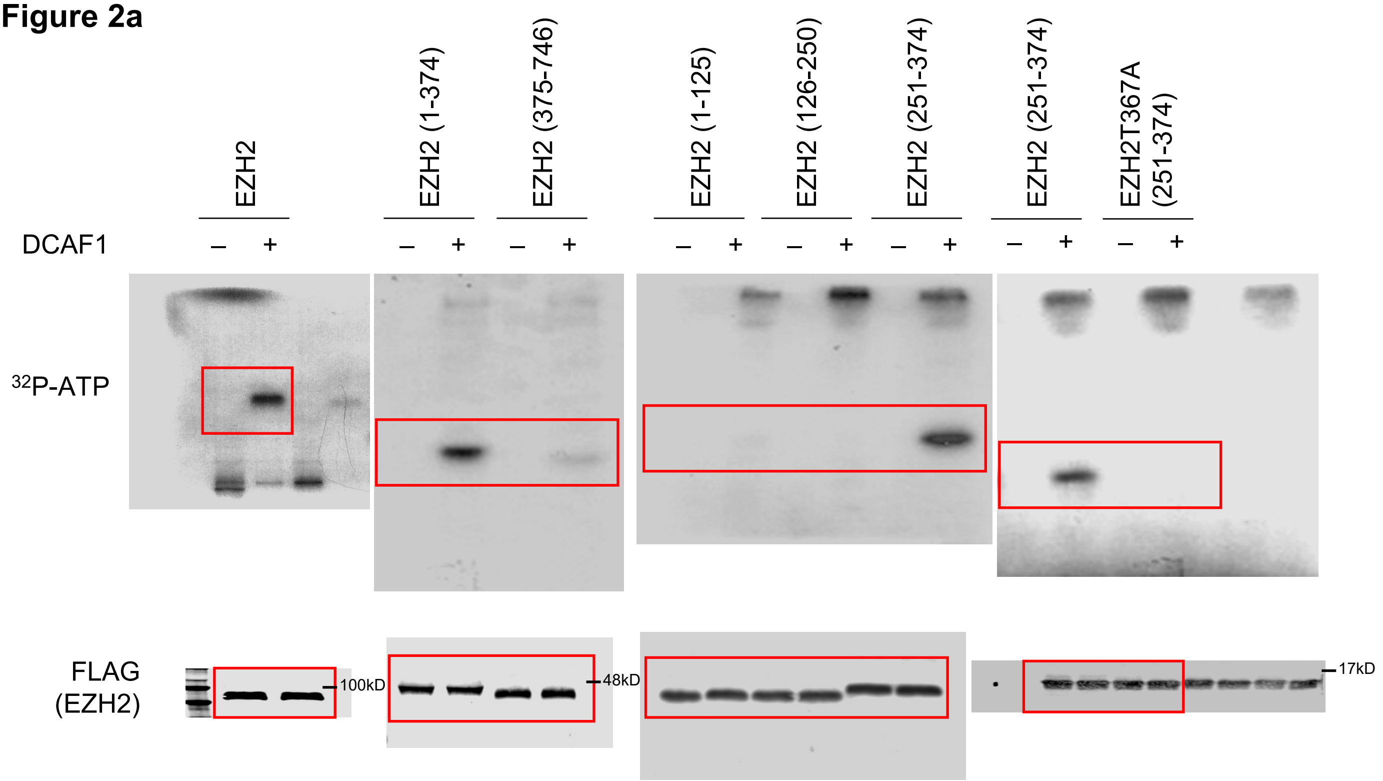

Figure 2c

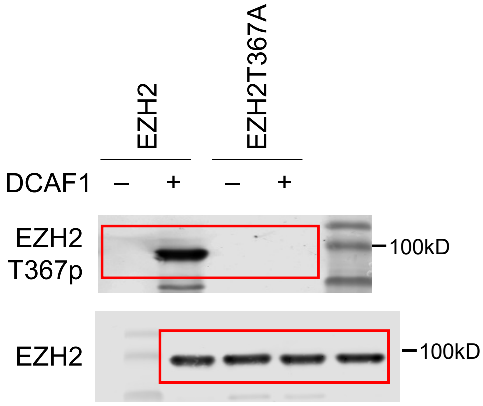

Figure 2d

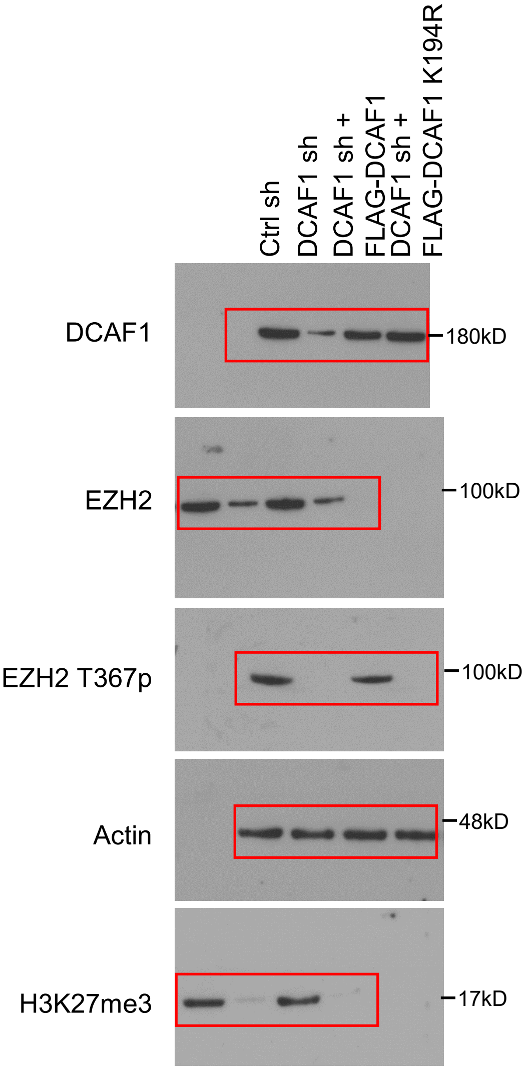

Figure 2e

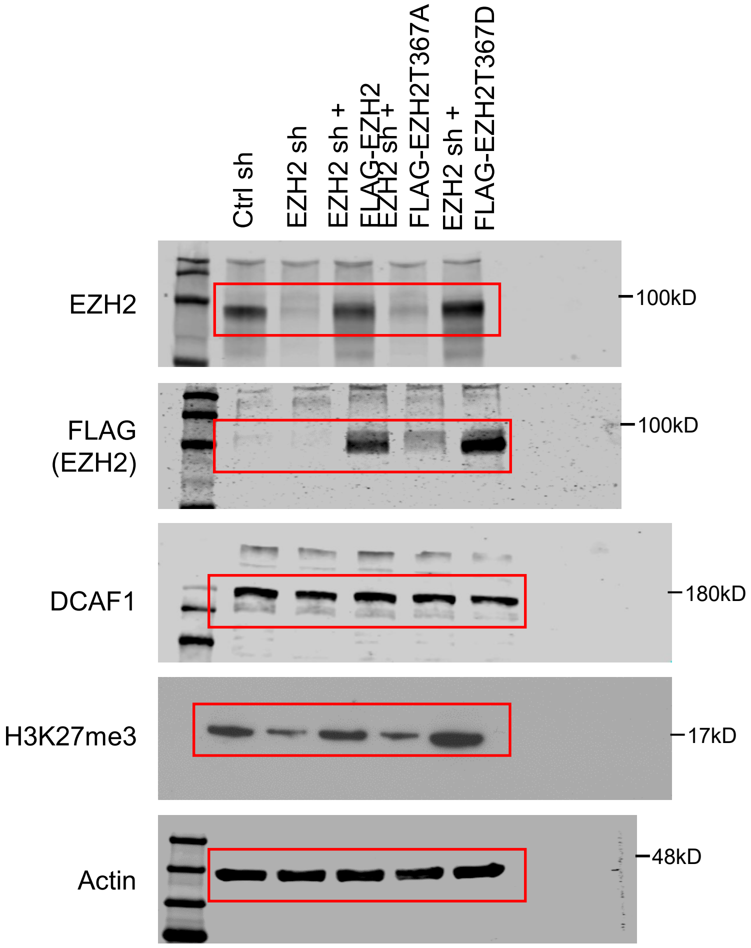

Figure 3a, left panel

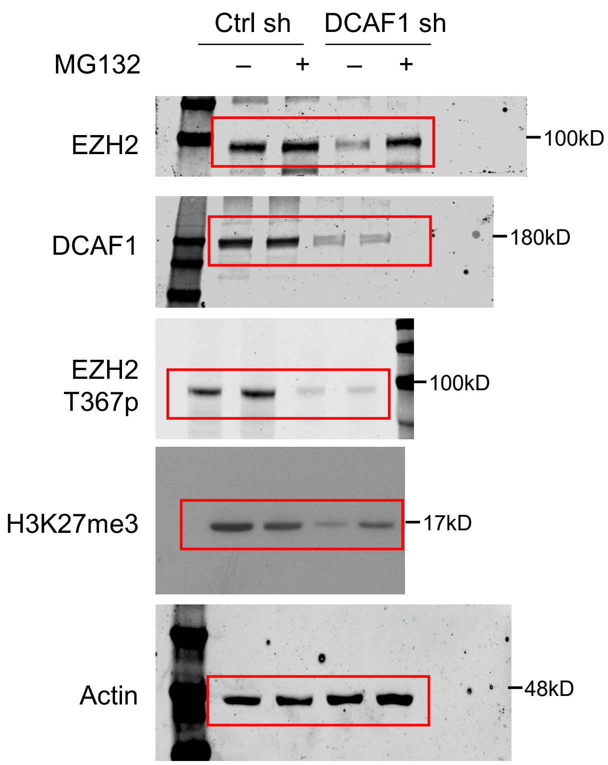

Figure 3a, right panel

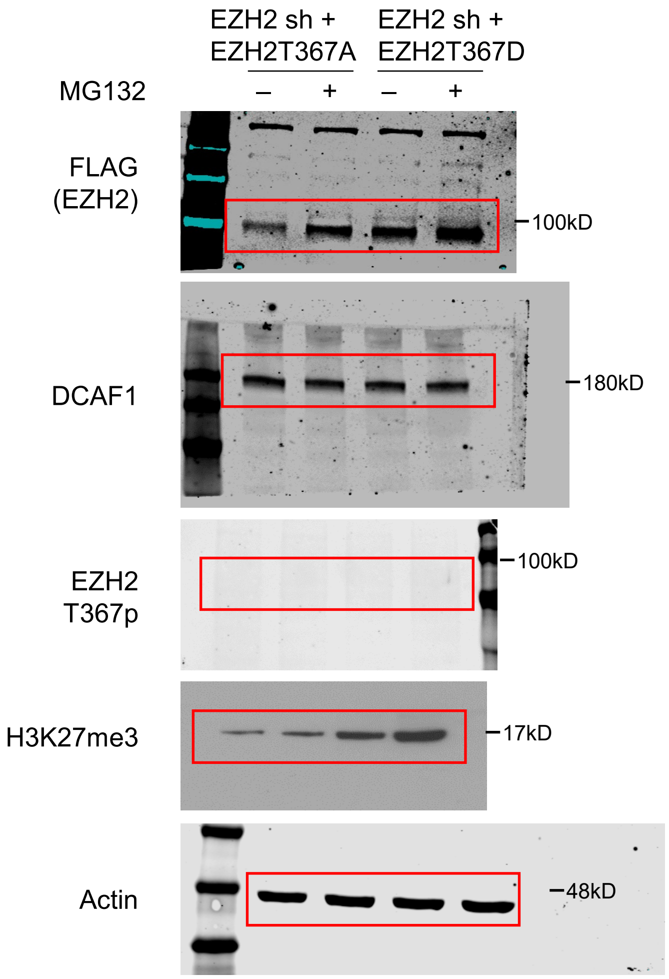

Figure 3b, left panel

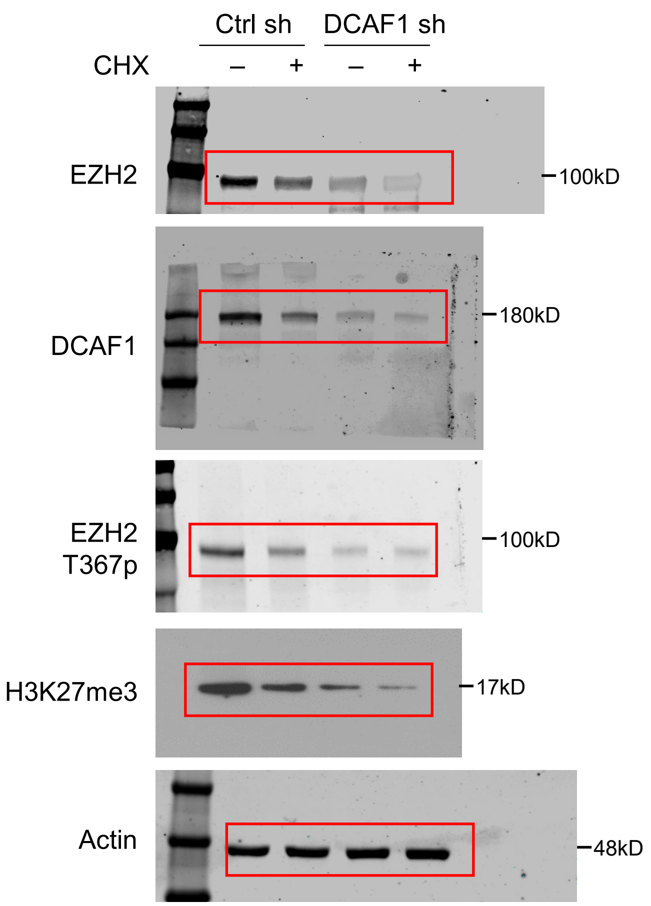

Figure 3b, right panel

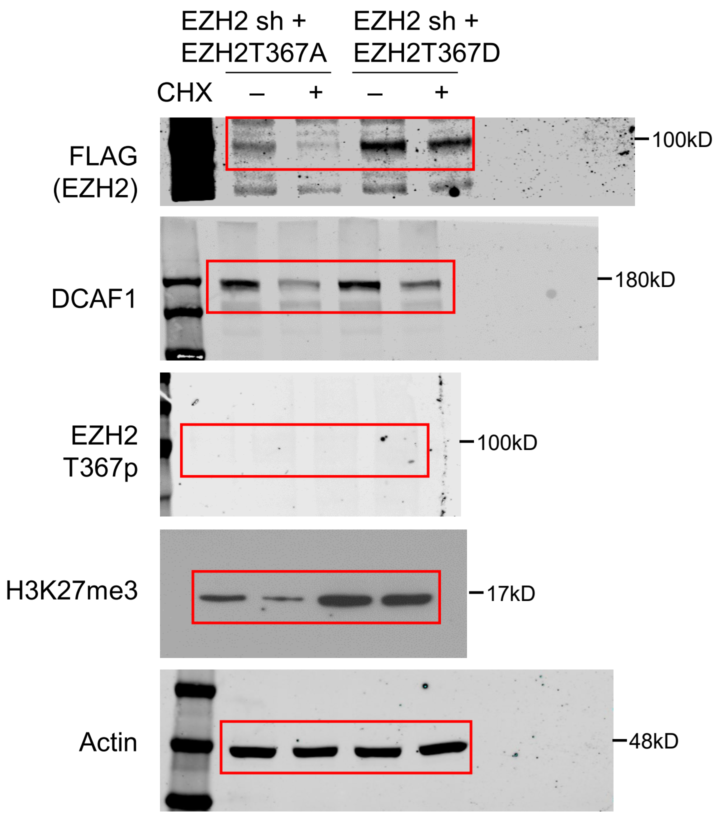

Figure 3d, left panel

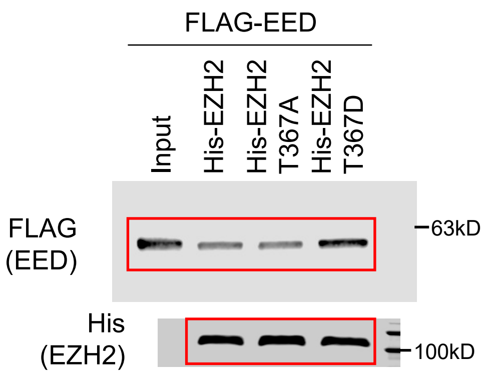

Figure 3d, middle panel

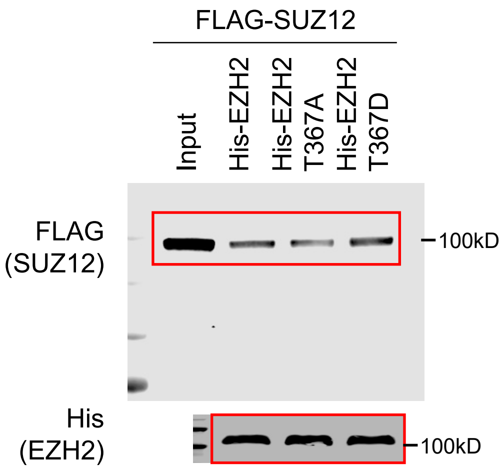

Figure 3d, right panel

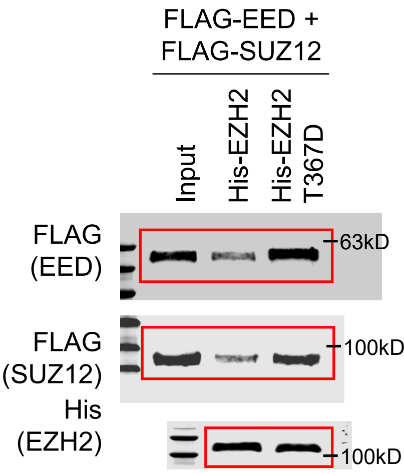

Figure 3e

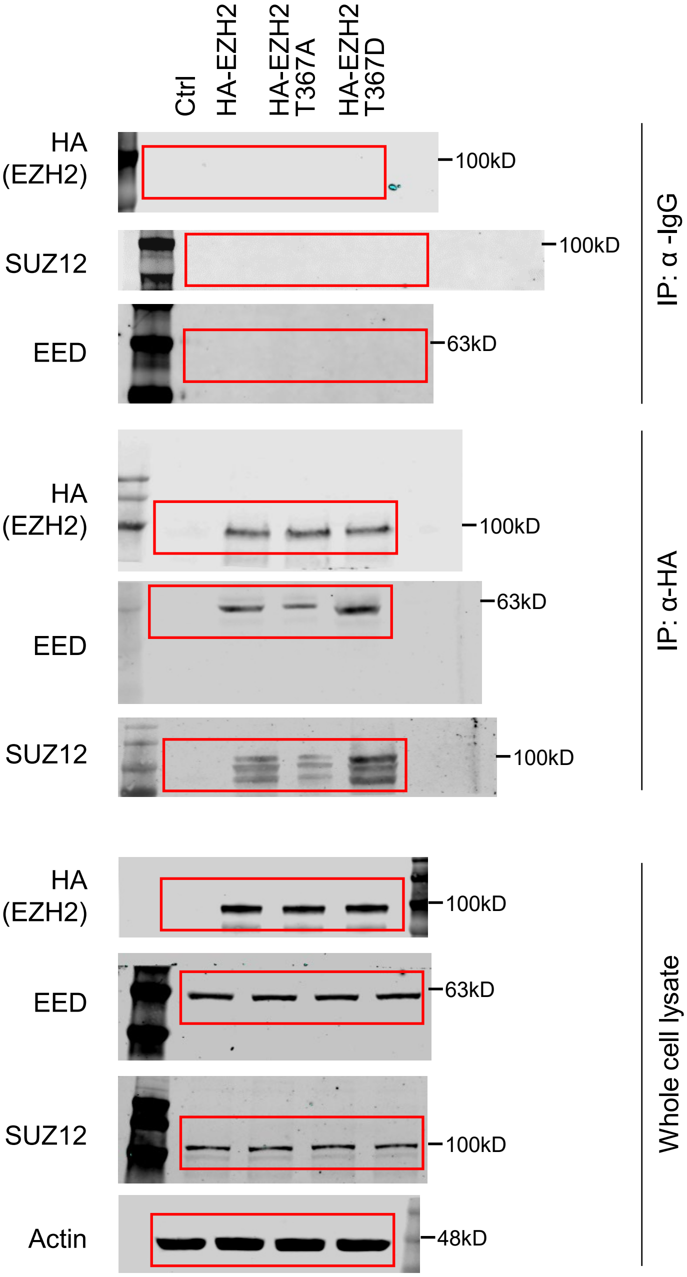
Ctrl
HA-EZH2
HA-EZH2 T367A
HA-EZH2 T367D
HA (EZH2)
100kD
SUZ12
100kD
EED
63kD
IP: α -IgG
HA (EZH2)
100kD
EED
63kD
SUZ12
100kD
IP: α-HA
HA (EZH2)
100kD
EED
63kD
SUZ12
100kD
Actin
48kD
Whole cell lysate

Figure 5c

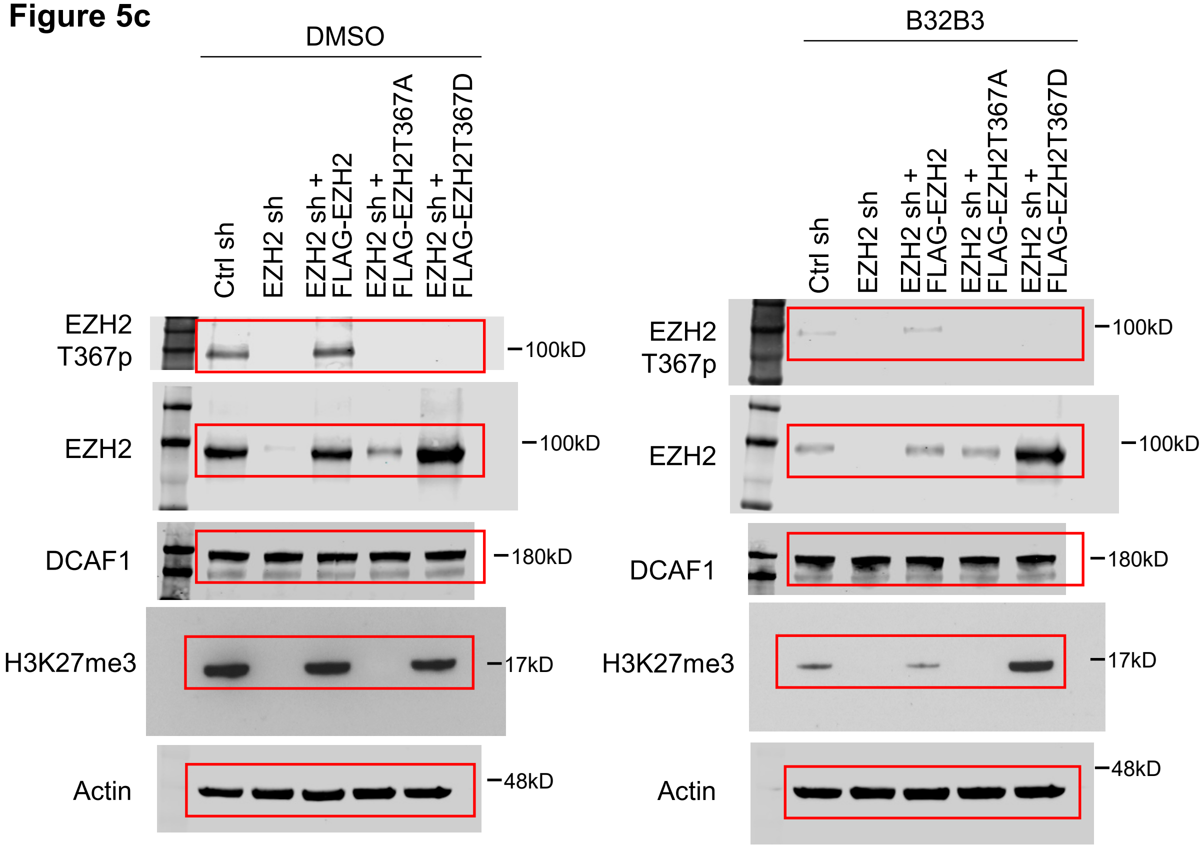

Figure 5f

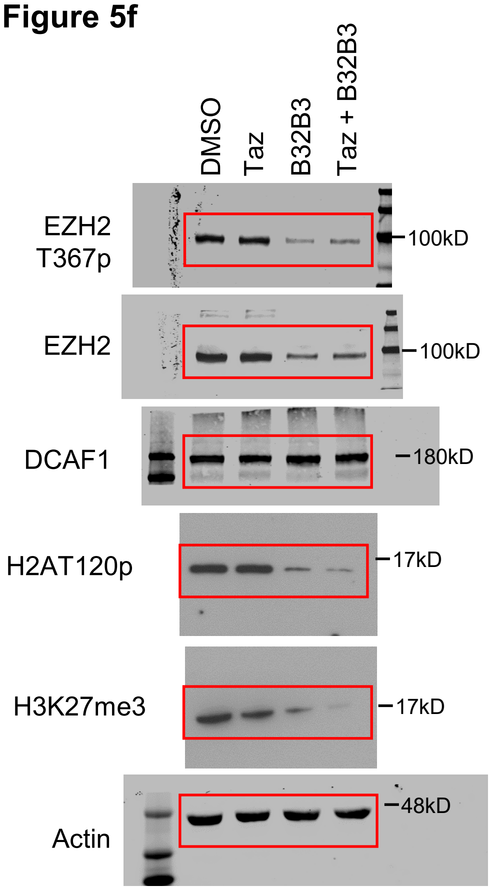

Figure 7d

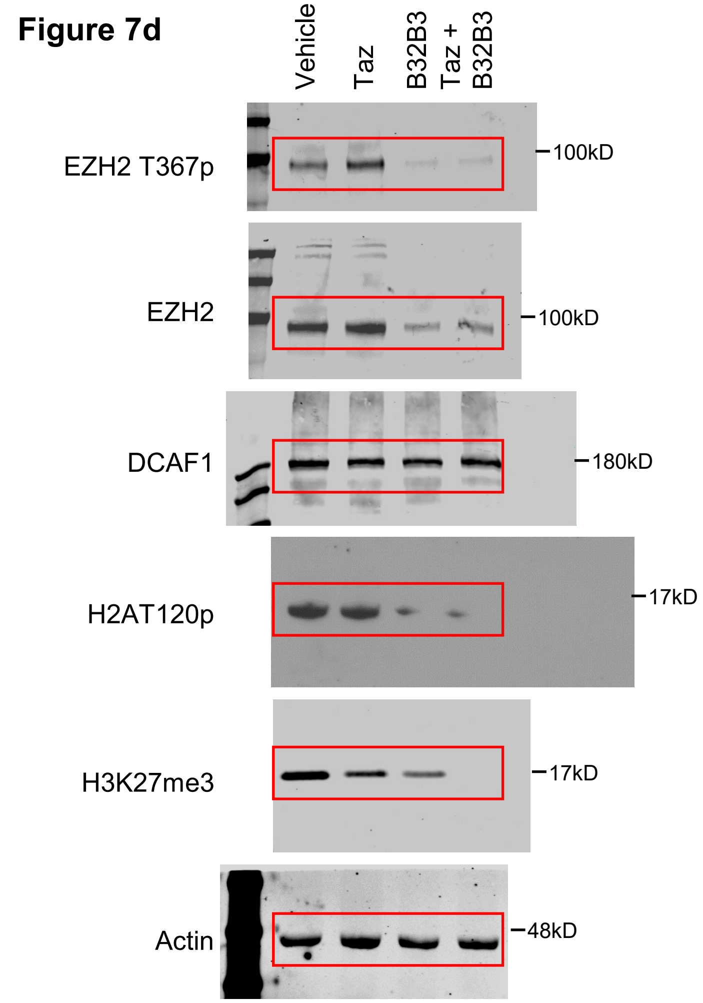

Supplementary Fig. 1a

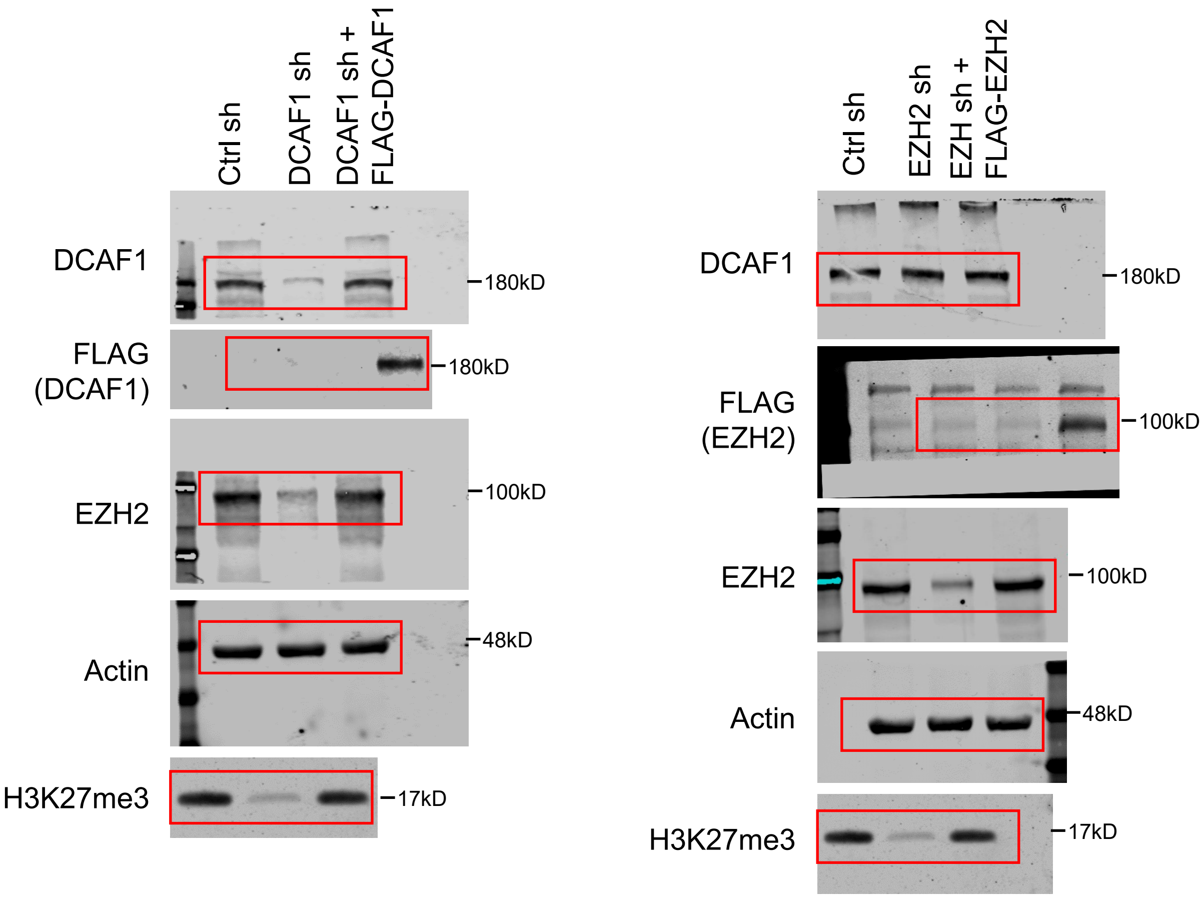

Supplementary Fig. 3

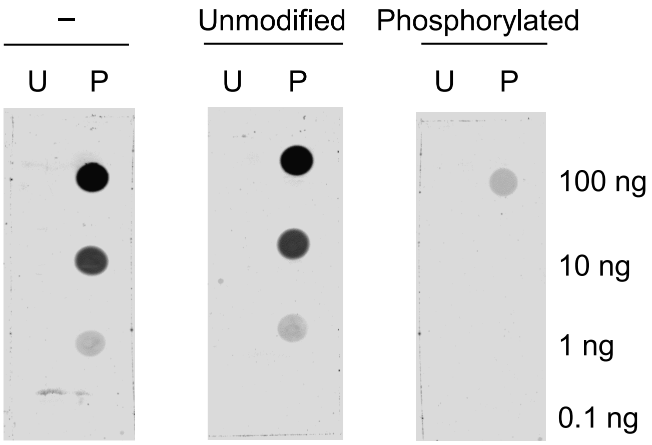

Supplementary Fig. 4

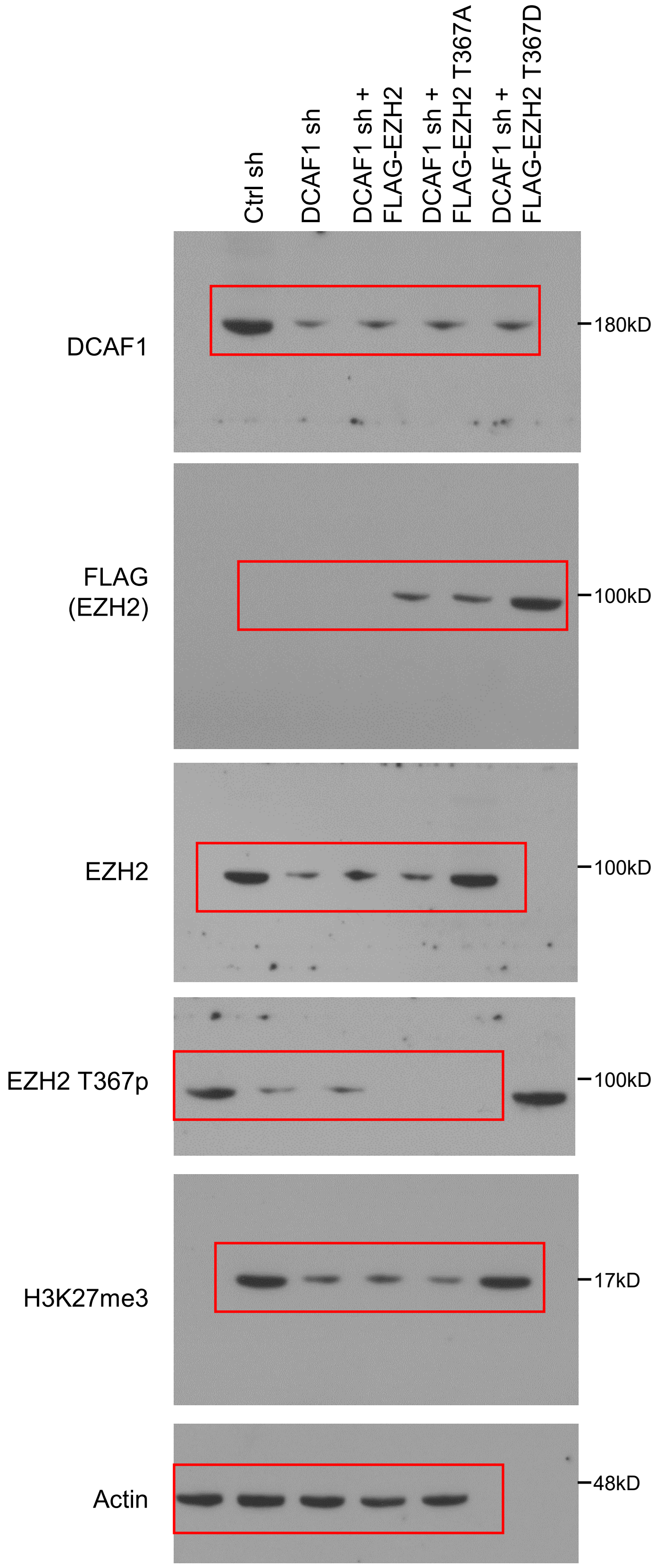

Supplementary Fig. 5a

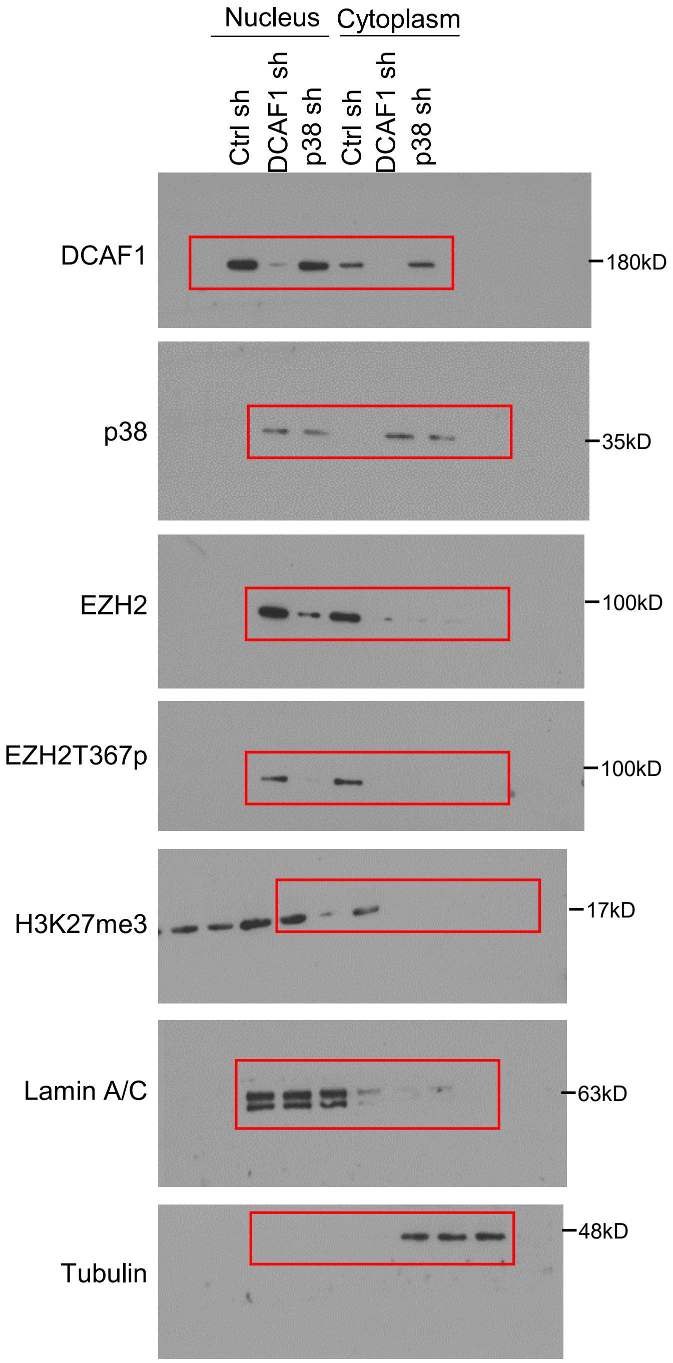

Supplementary Fig. 5b

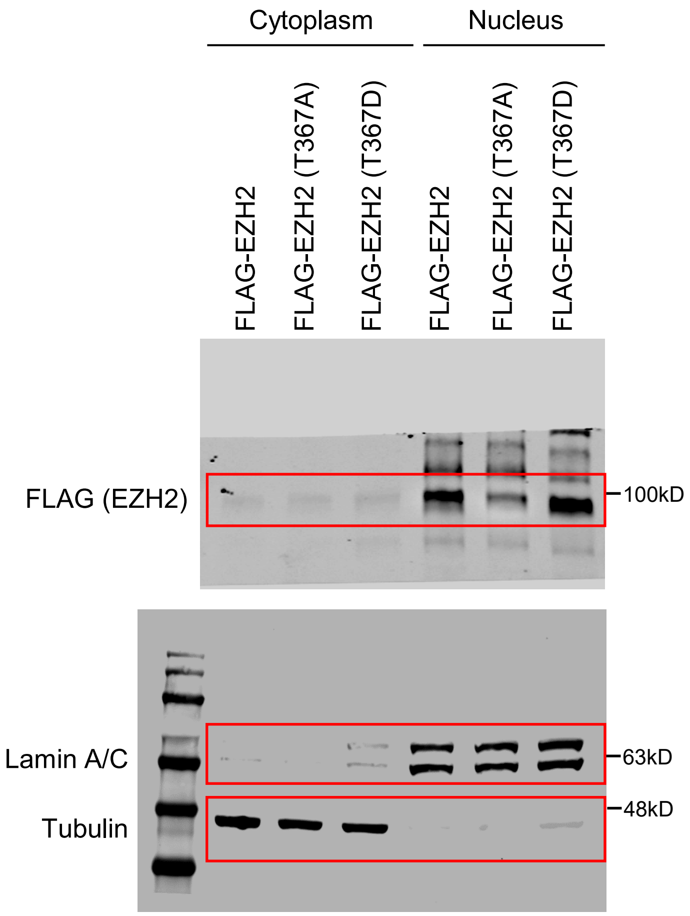

Supplementary Fig. 7

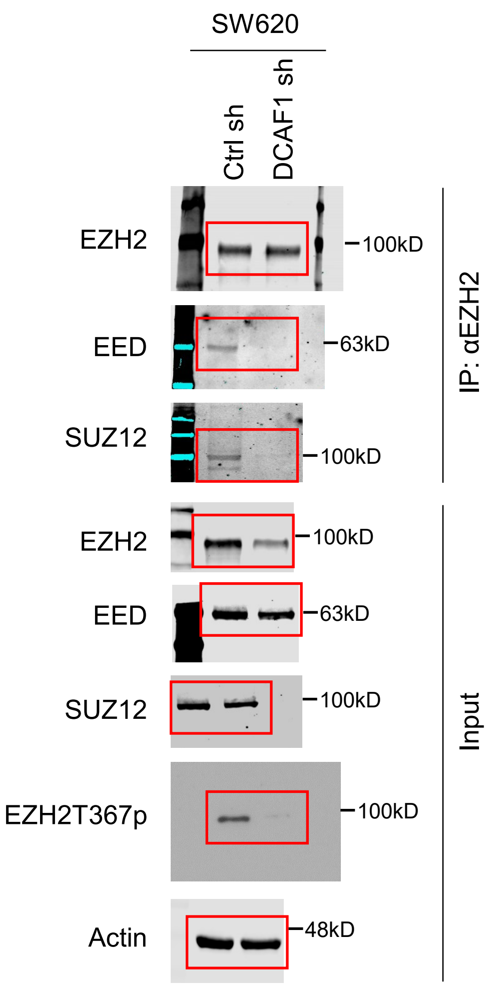

Supplementary Fig. 16

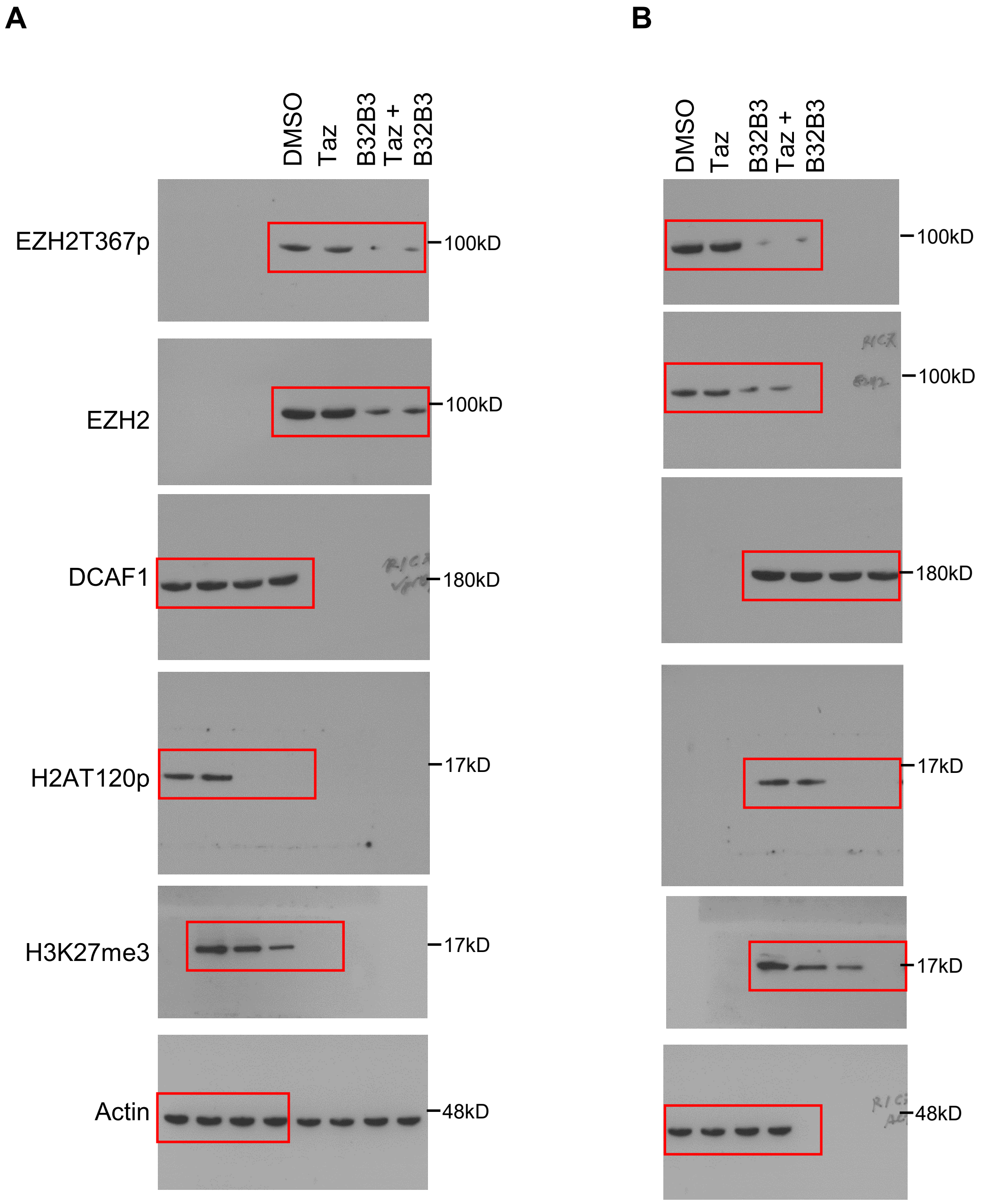

Supplementary Fig. 19a

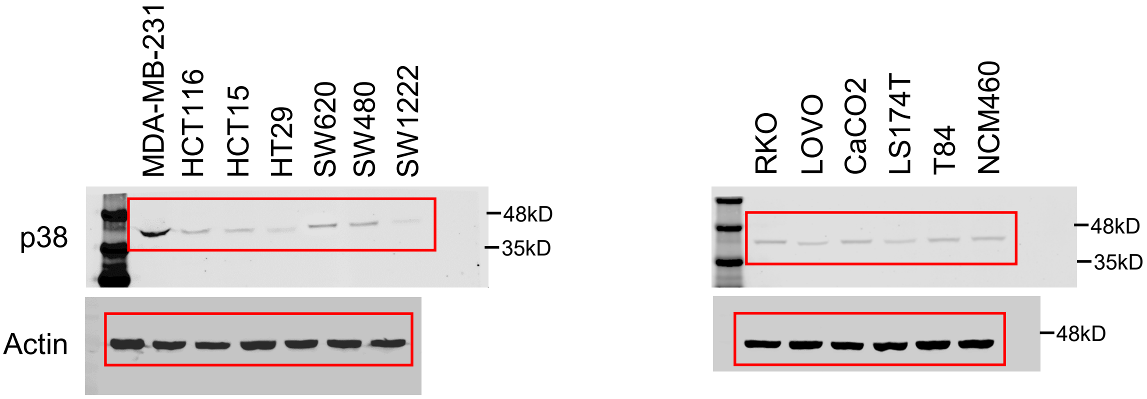

Supplementary Figure S19B

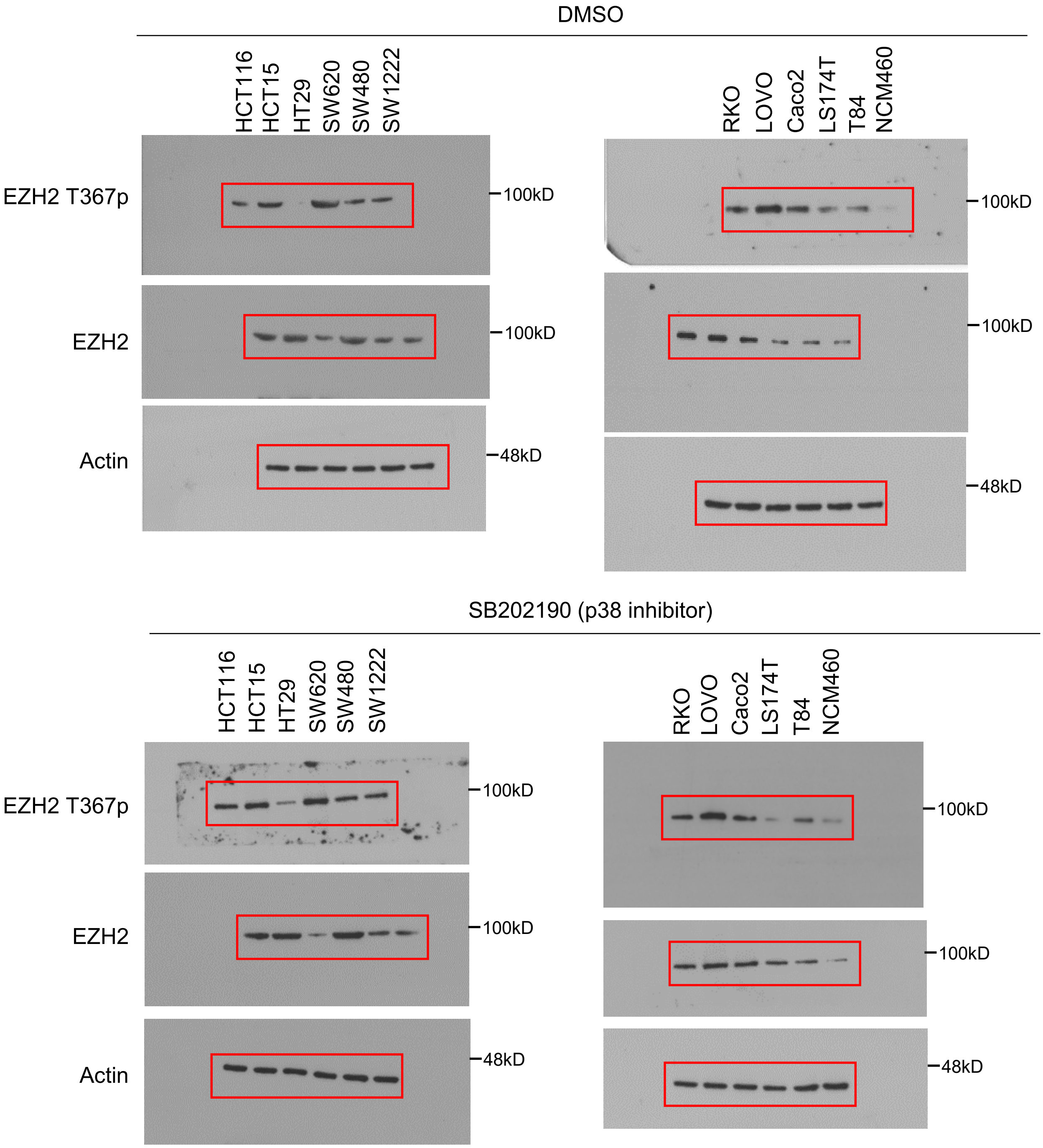

Supplementary Fig. 20

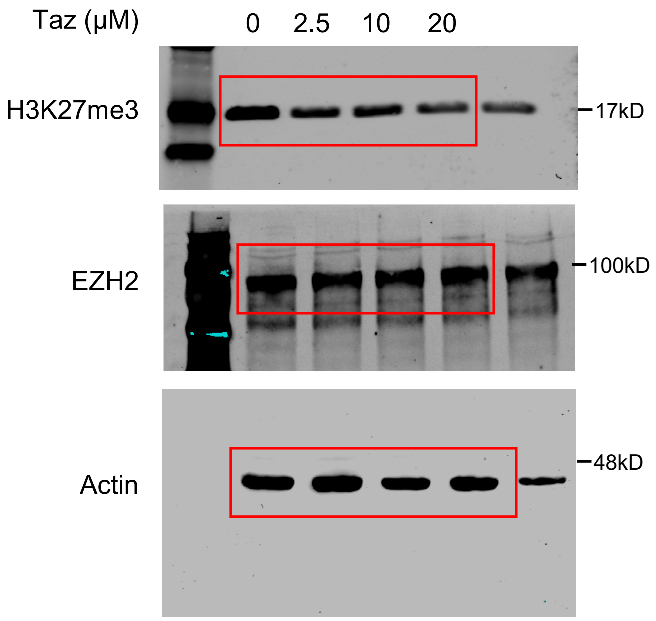

Supplement: Supplementary file 3 — Source Data [file 41467_2023_37883_MOESM3_ESM.zip › Source Data/Raw data for western blots.pdf]
